# Genotype-stratified adjunctive dexamethasone for tuberculous meningitis in HIV-negative adults: a randomized controlled phase 3 trial

**DOI:** 10.1038/s41591-025-04138-z

**Published:** 2026-01-15

**Authors:** Joseph Donovan, Nguyen Duc Bang, Huu Khanh Trinh Dong, Dang Trung Nghia Ho, Thi Anh Thu Nguyen, Thi Thu Hiep Nguyen, Hong Bao Ngoc Lam, Vo Khac Nguyen Phung, Truc Thanh Nguyen, Ho Hong Hanh Nguyen, Kieu Nguyet Oanh Pham, Dang Anh Thu Do, Thi Mai Trang Nguyen, Thi Minh Ha Dang, Huu Lan Nguyen, Van Vinh Chau Nguyen, Thanh Hai Hoang, Dinh Dinh Tran, Khanh Lam Phung, Lalita Ramakrishnan, Thanh Hoang Nhat Le, Thuy Thuong Thuong Nguyen, Marcel Wolbers, Evelyne Kestelyn, Ronald B. Geskus, Hoan Phu Nguyen, Guy E. Thwaites

**Affiliations:** 1 Oxford University Clinical Research Unit, Ho Chi Minh City, Vietnam; 2 Nuffield Department of Medicine, University of Oxford, Oxford, UK; 3 London School of Hygiene and Tropical Medicine, London, UK; 4 Pham Ngoc Thach Hospital for Tuberculosis and Lung Disease, Ho Chi Minh City, Vietnam; 5 King’s College London, London, UK; 6 Hospital for Tropical Diseases, Ho Chi Minh City, Vietnam; 7 Pham Ngoc Thach University of Medicine, Ho Chi Minh City, Vietnam; 8 School of Medicine, Vietnam National University of Ho Chi Minh City, Ho Chi Minh City, Vietnam; 9 MRC Laboratory of Molecular Biology, University of Cambridge, Cambridge, UK

**Keywords:** Phase III trials, Tuberculosis, Meningitis

## Abstract

Adjunctive corticosteroids such as dexamethasone are recommended in tuberculous meningitis treatment, despite modest and heterogeneous survival benefit. Leukotriene A4 hydrolase (*LTA4H*) genotypes associate with distinct intracerebral inflammatory phenotypes and may determine corticosteroid response in tuberculous meningitis, with benefit observed in hyperinflammatory TT genotype but uncertain benefit in lower inflammation CC and CT genotypes. Here, in a phase 3, placebo-controlled trial of human immunodeficiency virus-negative Vietnamese adults with tuberculous meningitis, we randomized 613 *LTA4H* CC- and CT-genotype participants to 6–8 weeks of dexamethasone or placebo, aiming to show noninferiority of placebo (hazard ratio margin of 0.75) or its superiority. Given the significant survival benefit of dexamethasone previously seen in *LTA4H* TT-genotype individuals, TT-genotype participants all received open-label dexamethasone and were not randomized. A total of 89 TT-genotype participants received open-label dexamethasone. In CC- and CT-genotype participants, the primary endpoint of all-cause death or new neurological event over 12 months from randomization occurred in 108/305 (35.4%) given dexamethasone and 110/308 (35.7%) given placebo (hazard ratio of 0.99, 96% confidence interval (adjusted for multiple testing) 0.748–1.31). The number of observed primary endpoints (*n* = 218) exceeded the prespecified number (*n* = 184) used to calculate the trial’s sample size and power. Placebo noninferiority was not established in the CC and CT population or in individual genotype subpopulations. Benefit or heterogeneity of effect was not observed by any prespecified subgroup. In TT-genotype participants, the primary endpoint occurred in 28/89 (31.5%) participants, similar to CC and CT participants. Outcomes were not significantly better in TT-genotype participants versus CC- or CT-genotype participants. In CC- and CT-genotype participants, serious adverse events occurred in 161/305 (52.8%) dexamethasone-treated participants and 160/308 (51.9%) placebo-treated participants. In conclusion, neither noninferiority nor superiority of placebo was established in human immunodeficiency virus-negative *LTA4H* CC- and CT-genotype adults with tuberculous meningitis, and dexamethasone was safe. The modest and heterogeneous benefit of dexamethasone indicates that greater understanding of tuberculous meningitis pathophysiology is needed, alongside better targeted, more effective anti-inflammatory agents than corticosteroids (ClinicalTrials.gov NCT03100786).

## Abstract (web summary)

In this phase 3 trial, human immunodeficiency virus-negative Vietnamese adults with tuberculous meningitis were stratified by *LTA4H* genotype to assess whether this influences corticosteroid response. However, outcomes were not significantly better according to genotype.

## Main

Tuberculous meningitis is a life-threatening brain infection caused by *Mycobacterium tuberculosis*^1^. In 2019, 164,000 adults were estimated to have suffered from tuberculous meningitis, of whom around 30% died^2^. It is the most lethal form of tuberculosis.

Tuberculous meningitis follows initial pulmonary *M. tuberculosis* infection, with dissemination of bacteria in the bloodstream to cause infectious foci in the brain (often called ‘Rich foci’ after the seminal pathophysiological studies conducted by Arnold Rich)^3,4^. These foci can subsequently introduce bacteria into the subarachnoid space, where the resultant inflammatory response heralds the onset of meningitis and the cardinal features of headache, fever and vomiting. As the bacteria replicate, so the inflammation increases, focal neurological deficits occur and consciousness falls, and without treatment, tuberculous meningitis is almost always fatal^5^.

Life-saving tuberculous meningitis treatment consists of antituberculosis drugs that kill *M. tuberculosis* and anti-inflammatory drugs that suppress harmful intracerebral inflammation^1^. While the initiation of antituberculosis drugs before the onset of coma substantially reduces tuberculous meningitis mortality^6^, the nature and severity of intracerebral inflammation before and after starting antituberculosis drugs is strongly associated with poor outcomes^7^.

Corticosteroids are broad-acting anti-inflammatory drugs that have long been hypothesized to suppress the harmful intracerebral inflammation associated with tuberculous meningitis. A randomized controlled trial of the corticosteroid dexamethasone, conducted in Vietnam and published in the year 2004^8^, found that dexamethasone significantly reduced overall mortality from tuberculous meningitis. However, benefit was uncertain in the 18% (98/545) of participants with human immunodeficiency virus (HIV) co-infection. Furthermore, substudies investigating dexamethasone’s effect on cerebrospinal fluid (CSF) inflammatory cytokines^9^ and brain inflammation imaging^10^ found marked interindividual heterogeneity of intracerebral inflammation and did not establish a mechanism of dexamethasone’s survival effect.

An explanation for these puzzling findings came with the discovery of a common functional promoter variant (C/T transition) in the gene encoding leukotriene A4 hydrolase (*LTA4H*), which determines eicosanoid-mediated high- and low-inflammation states during mycobacterial infection^11^. Genetic analysis of a subset of the Vietnam dexamethasone trial^8^ participants and subsequent tuberculous meningitis cohorts found that *LTA4H* genotypes were associated with distinct intracerebral inflammatory phenotypes and dexamethasone responsiveness in HIV-negative individuals but not in those living with HIV^7,12,13^. *LTA4H* TT genotype was associated with increased CSF cytokine concentrations and significant dexamethasone survival benefit, whereas dexamethasone did not appear to benefit lower inflammation CT and CC genotypes, possibly causing harm in CC-genotype individuals.

These findings suggested *LTA4H* genotype might be able to stratify HIV-negative individuals with tuberculous meningitis into those who benefit from dexamethasone and those who do not. We therefore conducted a *LTA4H*-genotype stratified randomized placebo-controlled trial of dexamethasone in HIV-negative Vietnamese adults with tuberculous meningitis. The benefit of dexamethasone in *LTA4H* CC- and CT-genotype adults (around 90% of the Vietnamese population) was uncertain; therefore, these individuals were randomized. We considered it unethical to randomize TT-genotype individuals to placebo; they therefore received open-label dexamethasone. We also assessed systemic and intracerebral inflammation in the trial participants before and during treatment, aiming to characterize the influence of *LTA4H* genotype on inflammation and dexamethasone response.

Our trial’s primary objective was to determine whether placebo is not worse or noninferior than dexamethasone in HIV-negative, CC- and CT-genotype adult patients when added to the first 6–8 weeks of antituberculosis treatment of tuberculous meningitis. In principle, administration of dexamethasone in CC- and CT-genotype patients would be discouraged if placebo could be shown to be noninferior to dexamethasone. However, as personalized administration of dexamethasone would necessitate rapid *LTA4H* genotype testing, which would complicate management, some evidence of harm of dexamethasone in the CC and CT population (or the CC or CT genotypes alone) would also be required to change clinical practice. We therefore adopted a hybrid trial-design approach that aimed to prove noninferiority of placebo first in the CC- and CT-genotype population, but also, superiority of placebo should dexamethasone prove harmful.

In our previous trial, dexamethasone treatment was associated with a reduced risk of death (relative risk, of 0.69, 95% confidence interval (CI) 0.52–0.92)^8^. At the time of the current trial’s design in 2017, a Cochrane meta-analysis of nine trials (1,337 participants) of adjunctive corticosteroids for tuberculous meningitis reported corticosteroids reduced deaths by almost one quarter (relative risk of 0.75, 95% CI 0.65–0.87)^14^. Based on these data, we set the noninferiority margin in favor of dexamethasone at a hazard ratio of 0.75. If placebo was noninferior to dexamethasone, our primary hypothesis, the lower limit of the hazard ratio CIs must be >0.75. However, our previous data suggested dexamethasone might be harmful^12^; we therefore also assumed a true hazard ratio of 1.15 in the CC and CT population. Using these assumptions, the trial was powered to establish the noninferiority of placebo.

## Results

**Fig. 1:**
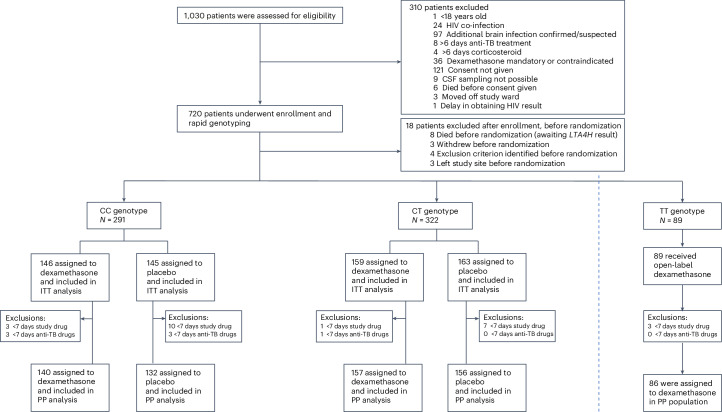
Screening, enrollment and randomization. ITT, intention-to-treat; PP, per-protocol; TB, tuberculosis.

From 12 February 2018 to 9 March 2023, 1,030 adults were screened for enrollment, and 720 adults were enrolled; 18 were not randomized because they died or were excluded before *LTA4H* genotype results returned (Fig. 1). Overall, 613 participants (291 CC genotype and 322 CT genotype) were randomized to receive either dexamethasone or placebo, and 89 TT-genotype participants received open-label dexamethasone. Six participants (0.9%) did not complete the 12-month follow-up (five of male sex, one of female sex). Of these six participants, three withdrew from the study, and three were lost-to-follow-up. A total of 31 participants were excluded for the per-protocol population (Supplementary Table 1). Major amendments made to the trial protocol after study start and major deviations from this protocol are described in the Methods.

### Participant disposition

The median age of all participants was 47 years (first to third quartiles 33–59 years). Disease was mostly mild or moderate (640/702 (91.2%), modified Medical Research Council (MRC) severity grade I or II), and the majority (553/702 (78.8%)) had definite or probable tuberculous meningitis. The enrollment antituberculosis chemotherapy included rifampicin in 695/702 (99.0%), started a median 3 days before randomization. Multidrug resistance was identified in six participants and isoniazid resistance (without rifampicin resistance) in 39 participants; all were treated according to national guidelines.

Baseline participant characteristics of those randomized (CC and CT genotypes) and by genotype were well balanced. However, TT-genotype participants were younger (median 42 years (first to third quartiles 28–59 years) versus median 47 years (first to third quartiles 34–59 years) for CC- and CT-genotype participants), were more likely to have microbiological confirmation of tuberculous meningitis (45/89 (50.6%) definite tuberculous meningitis versus 267/613 (43.6%) for CC- and CT-genotype participants) and had more severe disease at baseline (16/89 (18.0%) modified MRC severity grade III versus 46/613 (7.5%) for CC- and CT-genotype participants) (Table 1 and Supplementary Tables 2 and 3). The participants were broadly representative of populations of persons with tuberculous meningitis (Supplementary Table 4).

**Table 1:** Baseline characteristics in the intention-to-treat population, including TT genotype

Characteristic	*N*	All participants (*N* = 702)	CC and CT genotype (*N* = 613)		TT genotype (*N* = 89)
Treatment	702		Dexamethasone (*N* = 305)	Placebo (*N* = 308)	Dexamethasone (*N* = 89)
Age (years)	702	47 (33–59)	48 (35–60)	46 (33–58)	42 (28–59)
Male sex	702	435 (62.0%)	192 (63.0%)	191 (62.0%)	52 (58.4%)
*LTA4H* CC genotype	702	291 (41.5%)	146 (47.9%)	145 (47.1%)	–
*LTA4H* CT genotype	702	322 (45.9%)	159 (52.1%)	163 (52.9%)	–
Diagnostic category	702	–	–	–	–
Definite	–	312 (44.4%)	128 (42.0%)	139 (45.1%)	45 (50.6%)
Probable	–	241 (34.3%)	107 (35.1%)	108 (35.1%)	26 (29.2%)
Possible	–	149 (21.2%)	70 (23.0%)	61 (19.8%)	18 (20.2%)
Confirmed non-TBM	–	0 (0%)	0 (0%)	0 (0%)	0 (0%)
Modified MRC severity grade	702	–	–	–	–
Grade I	–	298 (42.5%)	134 (43.9%)	132 (42.9%)	32 (36.0%)
Grade II	–	342 (48.7%)	150 (49.2%)	151 (49.0%)	41 (46.1%)
Grade III	–	62 (8.8%)	21 (6.9%)	25 (8.1%)	16 (18.0%)
Glasgow coma score	698	15 (13–15)	15 (13–15)	15 (13–15)	14 (11–15)
Duration of symptoms (days)	702	15 (12–21)	15 (11–22)	15 (11–21)	16 (12–20)
Cranial nerve palsy	702	86 (12.3%)	36 (11.8%)	39 (12.7%)	11 (12.4%)
Hemiplegia	702	76 (10.8%)	35 (11.5%)	26 (8.4%)	15 (16.9%)
Paraplegia/tetraplegia	702	125 (17.8%)	52 (17.0%)	56 (18.2%)	17 (19.1%)
CSF parameters	–	–	–	–	–
Total leucocytes (cells per cubic millimeter)	702	136 (33–326)	127 (31–309)	143 (35–349)	129 (33–294)
Protein (g l^−1^)	702	1.5 (0.9–2.2)	1.4 (0.9–2.2)	1.5 (0.9–2.3)	1.5 (1.0–2.2)
CSF/blood glucose ratio	701	0.37 (0.25–0.49)	0.37 (0.27–0.47)	0.37 (0.24–0.51)	0.38 (0.23–0.48)
CSF microbiological tests	–	–	–	–	–
Positive ZN stain	702	153 (21.8%)	68 (22.3%)	65 (21.1%)	20 (22.5%)
Positive GeneXpert MTB/RIF	702	102 (14.5%)	45 (14.8%)	44 (14.3%)	13 (14.6%)
Positive GeneXpert MTB/RIF Ultra^a^	702	116 (16.5%)	46 (15.1%)	58 (18.8%)	12 (13.5%)
Positive mycobacterial culture	702	175 (24.9%)	77 (25.2%)	72 (23.4%)	26 (29.2%)
Duration of antituberculosis chemotherapy before enrollment (days)	700	3.0 (1.0–4.0)	3.0 (1.0–4.0)	3.0 (1.0–4.0)	2.0 (2.0–4.0)

^a^Xpert Ultra availability varied between sites and over time but only became more widely available for the last 12 months of the study, hence its relatively restricted use. *N* is the number of participants included in that statistic. The summary statistic is the median (first and third quartile) value for continuous data, and the number and frequency (%) of participants with the characteristic for categorical data. Definite TBM is defined as positive acid-fast bacilli on CSF Ziehl-Neelsen stain, or positive CSF GeneXpert test, or positive CSF mycobacterial culture. Probable or possible TBM was defined following uniform case definition^26^. Confirmed non-TBM indicates microbiologically confirmed other brain infection. Confirmed additional brain infection includes positive CSF India Ink stain, CSF cryptococcal antigen, positive blood cryptococcal antigen, positive CSF bacterial Gram stain, positive CSF bacterial culture or positive CSF viral or helminth PCR test. Xpert, Gene Xpert MTB/RIF; ZN, Ziehl-Neelsen.

**Fig. 2:**
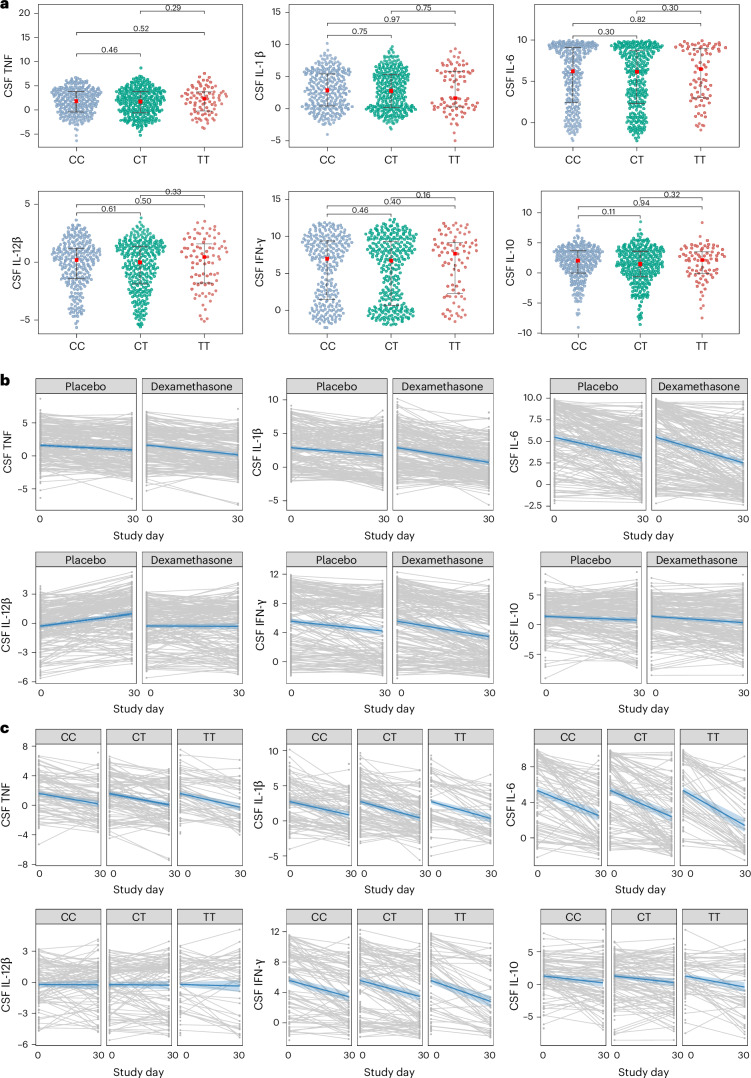
CSF cytokine concentrations at baseline according to *LTA4H* genotype, changes in CSF cytokine concentrations over time in CC- and CT-genotype participants randomized to dexamethasone or placebo and changes in cytokine concentrations over time in dexamethasone-treated CC-, CT- and TT-genotype participants. **a**, The distribution of CSF cytokine levels (normalized protein expression (NPX), log_2_ normalized expression) across different genotypic groups (CC, CT and TT). Each subplot represents a distinct cytokine: TNF, IL-1β, IL-6, IL-12β, IFN-γ and IL-10. The violin plots illustrate the data distribution for each genotype, with individual data points shown as dots. Each red central point indicates the median cytokine level within each group, and the horizontal black bars indicate the interquartile range. Pairwise comparisons between genotype groups were performed using the Wilcoxon rank-sum test (two-sided), with the corresponding *P* values displayed. The cytokine data are shown for a total of 638 participants (262 of CC genotype, 295 of CT genotype and 81 of TT genotype), for each cytokine subpanel. Eight participants (of 646 individuals who underwent proteomic profiling) did not have proteomic profiling data at baseline. **b**, CSF cytokine changes over 30 days, by placebo and dexamethasone arms. The solid blue lines show the fit mean cytokine levels at each timepoint, with shaded 95% Bayesian credible intervals obtained via Markov Chain Monte Carlo (MCMC) estimation from the longitudinal submodel of the Bayesian joint model. Rates of change of CSF cytokines for CC- and CT-genotype participants were as follows: for TNF, placebo −0.023, dexamethasone −0.049; for IL-1β, placebo −0.038, dexamethasone −0.072; for IL-6, placebo −0.078, dexamethasone −0.098; for IL-12β, placebo 0.041, dexamethasone −0.002; for IFN-γ, placebo −0.044, dexamethasone −0.070; for IL-10, placebo −0.021, dexamethasone −0.035. The cytokine data are shown for 281 participants allocated to dexamethasone (data shown for 278 participants at day 0 and 175 participants at day 30) and for 283 participants allocated to placebo (data shown for 279 participants at day 0 and 166 participants at day 30) for each cytokine subpanel. **c**, CSF cytokine changes over 30 days in dexamethasone-treated participants, by *LTA4H* genotype. The solid blue lines show the fit mean cytokine levels at each timepoint, with shaded 95% Bayesian credible intervals obtained via MCMC estimation from the longitudinal submodel of the Bayesian joint model. The cytokine data are shown for 363 dexamethasone-treated participants: for 136 participants of CC genotype (134 participants at day 0 and 83 participants at day 30), for 145 participants of CT genotype (144 participants at day 0 and 92 participants at day 30) and for 82 participants of TT genotype (81 participants at day 0 and 50 participants at day 30).

Baseline characteristics of participants with CSF (*n* = 646) and systemic (*n* = 202) inflammatory measurements were broadly representative of all participants (Supplementary Tables 5 and 6). There were no significant differences in baseline CSF inflammatory profiles among all genotypes. log_2_ normalized expressions of tumor necrosis factor (TNF), Interleukin (IL)-1β, IL-12β, interferon gamma (IFN-ɣ), IL-6 and IL-10 were not significantly different at baseline between CC- and CT-genotype participants. Likewise, activity of six key CSF inflammatory pathways did not differ at baseline between CC and CT groups. Unexpectedly, neither CSF cytokine expression nor CSF inflammatory pathway activity differed between TT-genotype participants and CC- or CT-genotype participants (Fig. 2a and Supplementary Fig. 1). Baseline whole blood inflammatory profiles were also similar between genotypes, except CC-genotype participants had greater systemic neutrophil activation and neutrophil degranulation (both *P* = 0.04), compared with CT-genotype participants (Supplementary Fig. 2).

### Prespecified primary and secondary outcomes

The primary outcome of the LAST ACT trial was death or a new neurological event over the first 12 months after randomization. Secondary endpoints of the LAST ACT trial were as follows: death over the first 12 months after randomization; first new neurological event over the first 12 months after randomization; use of open-label corticosteroid treatment for any reason over the first 12 months after randomization; neurological disability (defined as modified Rankin score ≥3) at 12 months from randomization; all modified Rankin scores as an ordinal scale at 12 months from randomization; measurements of blood and CSF inflammation; and severe (grade 3 and 4) and serious adverse events until 12 months from randomization. All outcomes are presented in this manuscript.

### Primary outcome

**Fig. 3:**
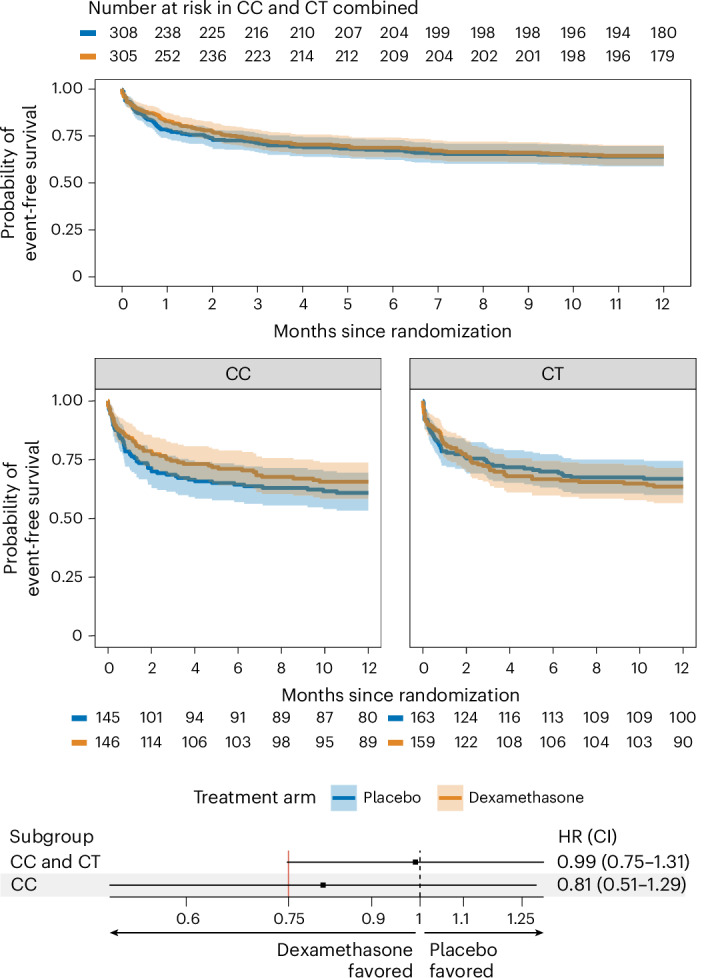
Death or new neurological events during the first 12 months after randomization for the intention-to-treat population. Kaplan–Meier curves are shown for the primary endpoint. The solid lines represent the estimated probability of event-free survival in each stratum. The colored shading represents the 95% CIs. A forest plot is shown displaying the hazard ratio (HR) and CIs, with corresponding confidence levels at 96% for CC and CT combined and 98.3% for CC. The points represent the estimated HRs in the primary endpoint between two treatment arms. The black lines represent the 95% CIs. The red vertical line demonstrates the noninferiority threshold at HR of 0.75. The dashed vertical line demonstrates the superiority threshold at HR of 1. For analysis, a Cox proportional hazard model was used, with MRC grade and *LTA4H* genotype as strata. Analyses are performed for the following populations: for CC and CT genotype combined, 613 participants; for CT genotype, 322 participants; and for CC genotype, 291 participants.

In randomized CC- and CT-genotype participants, death or new neurological events occurred in 108/305 (35.4%) given dexamethasone and 110/308 (35.7%) given placebo (hazard ratio of 0.99). To correct for performing an additional test in the CC-genotype subgroup, the CI was set at 96%, leaving 1.72% for the CC-genotype subgroup. The resulting CI for the combined CC and CT group just covered the noninferiority margin of 0.75 (CI 0.748–1.31) (Fig. 3, Table 2 and Supplementary Table 7). Hence the non-inferiority of placebo was not established. In the CC-genotype subgroup, death or new neurological events occurred in 50/146 (34.2%) of participants given dexamethasone and 56/145 (38.6%) given placebo (hazard ratio of 0.81, 98.3% CI 0.51–1.29).

**Table 2:** Analysis of primary outcome and prespecified subgroups in the CC- and CT-genotype intention-to-treat population

	Dexamethasone (*n* = 305)	Placebo (*n* = 308)	Comparison	Test for heterogeneity^a^
	events/*n* (risk (%))	events/*n* (risk (%))	Hazard ratio (95% CI)	*P* value
*LTA4H* genotype	–	–	–	0.32
CC	50/146 (34.2)	56/145 (38.6)	0.83 (0.57–1.22)	–
CT	58/159 (36.5)	54/163 (33.1)	1.09 (0.76–1.59)	–
TBM diagnosis	–	–	–	0.58
Definite	49/128 (38.3)	57/139 (41.0)	0.87 (0.59–1.28)	–
Probable	43/107 (40.2)	37/108 (34.3)	1.18 (0.76–1.83)	–
Possible	16/70 (22.9)	16/61 (26.2)	0.88 (0.44–1.75)	–
Modified MRC severity grade	–	–	–	0.75
Grade I	21/134 (15.7)	24/132 (18.2)	0.86 (0.48–1.54)	–
Grade II	70/150 (46.7)	69/151 (46.0)	0.97 (0.70–1.35)	–
Grade III	17/21 (81.0)	17/25 (68.0)	1.17 (0.60–2.30)	–
Antituberculosis drug resistance^~^	–	–	–	0.48
Multidrug resistant or rifampicin mono-resistant	2/5 (40.0)	1/3 (33.3)	0.99 (0.09–11.01)	–
Isoniazid resistant without rifampicin resistance	6/18 (33.3)	10/16 (62.5)	0.46 (0.17–1.28)	–
No or other resistance	25/53 (47.2)	25/54 (46.3)	0.93 (0.53–1.62)	–
*M. tuberculosis* not isolated or missing result	74/228 (32.5)	74/235 (31.5)	1.02 (0.74–1.40)	–

^a^Heterogeneity was tested by a likelihood ratio test between two Cox models fitted on the full population, one with an interaction term between treatment arm and the subgroup covariable and one without. No correction for multiplicity is made. The primary endpoint was death from any cause or new neurological event over the first 12 months after randomization. This table reports the results from the Cox proportional hazards regression model. Hazard ratios and the CIs were estimated by a univariate Cox’s proportional hazard model in each subgroup. The primary effect measure was the resulting hazard ratio comparing dexamethasone versus placebo with a corresponding two-sided 95% CI. The proportional-hazards assumption was tested for each model and was not violated (*P* > 0.05). In subgroup analyses, a separate Cox model was fit for each value of the subgroup. The test for heterogeneity was based on the likelihood ratio test that includes subgroup as covariate and compares the models with subgroup as main effect only and with subgroup as treatment effect modifier.

The superiority analyses did not correct for multiple testing. Superiority of dexamethasone or placebo was neither observed in the CC- and CT-genotype group nor in the individual genotype subgroups. Death or new neurological events occurred in 54/163 (33.1%) CT-genotype participants given placebo compared with 58/159 (36.5%) given dexamethasone (hazard ratio of 1.09, 95% CI 0.76–1.59).

Heterogeneity of effect was not observed by prespecified subgroups of tuberculous meningitis diagnosis, modified MRC grade or antituberculosis drug resistance (Table 2, Supplementary Tables 8 and 9 and Supplementary Fig. 4). A per-protocol analysis produced similar findings (Supplementary Tables 10 and 11 and Supplementary Fig. 5); in randomized CC- and CT-genotype participants within the per-protocol population, death or new neurological events occurred in 103/297 (34.7%) given dexamethasone and 101/288 (35.1%) given placebo (hazard ratio of 1.00, 96% CI 0.75–1.33). Restricted mean time lost (RMTL) analyses (Supplementary Tables 8, 9 and 12) did not demonstrate superiority of dexamethasone versus placebo in intention-to-treat or per-protocol populations or within any predefined subgroups.

**Fig. 4:**
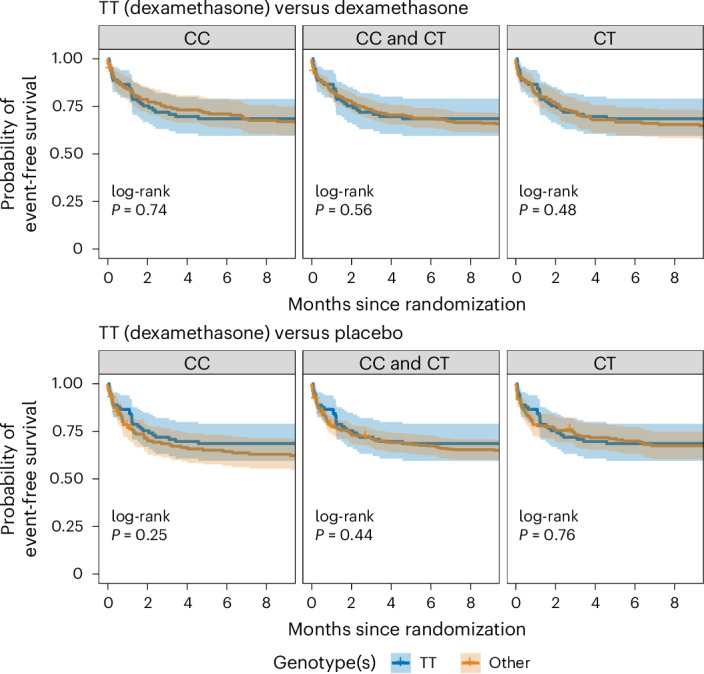
Death or new neurological events for all genotypes. A comparison of TT-genotype individuals receiving dexamethasone with CC, CC and CT, and CT-genotype individuals receiving dexamethasone. This is followed by comparison of TT-genotype individuals receiving dexamethasone with CC, CC and CT, and CT-genotype individuals receiving placebo. The solid lines represent the estimated probability of event-free survival in each stratum. The colored shading represents the 95% CIs. Analyses are performed for the following populations: TT-genotype participants receiving dexamethasone, 89; CC-genotype participants receiving dexamethasone, 146; CT-genotype participants receiving dexamethasone, 159; CC-genotype participants receiving placebo, 145; and CT-genotype participants receiving placebo, 163.

In TT-genotype participants, the primary outcome frequency (28/89 (31.5%)) was similar to CC- and CT-genotype participants (35.6%) regardless of treatment allocation (Fig. 4 and Supplementary Table 13). An exploratory analysis suggested that TT-genotype survival may be superior to CC and CT genotype in those with severe disease (modified MRC severity grade 3) and that an imbalance in disease severity may have skewed the overall similarity of TT versus non-TT groups (Supplementary Fig. 6).

### Secondary outcomes

There were an equal number of deaths in the dexamethasone and placebo arms (66/305 (21.4%) and 66/308 (21.4%), respectively). First new neurological event, neurological disability and use of open-label corticosteroid treatment for any reason occurred with similar frequency in both treatment arms in CC- and CT-genotype intention-to-treat and per-protocol populations, with no heterogeneity of effect identified in planned subgroup analyses (Supplementary Tables 14–43 and Supplementary Figs. 7–16). Open-label corticosteroids were used in 69/394 (17.5%) participants allocated to dexamethasone and in 50/308 (16.2%) allocated to placebo. Reasons for open-label corticosteroid use are given in Supplementary Table 27; the commonest reason was depressed level of consciousness (*N* = 11 dexamethasone group, *N* = 10 placebo group). Within CC-genotype participants, there was less disability in dexamethasone-treated participants (by modified Rankin score as an ordinal scale, 1–6) at 12 months, cumulative odds ratio of 0.63 (95% CI 0.40–0.97).

We sought to investigate the effects of *LTA4H* genotype and dexamethasone on inflammation, with CSF inflammatory proteins and whole blood transcriptomes as planned secondary outcomes. In all randomized (CC- and CT-genotype) participants, the dexamethasone group had a greater rate of reduction of CSF TNF, IL-1β, IL-12β and IFN-ɣ (but not IL-10 and IL-6) concentrations than placebo, from day 0 to day 30 (Fig. 2b and Extended Data Table 1). Similarly, the dexamethasone group had a greater rate of reduction of CSF IFN signaling, neutrophil degranulation, neutrophil activation, eicosanoid metabolic processes, TNF signaling and cytokine signaling, than placebo (Extended Data Table 2 and Supplementary Fig. 17).

There was evidence that *LTA4H* genotype influenced dexamethasone-associated CSF effects (Fig. 2c and Supplementary Fig. 18). In CC-genotype participants, the dexamethasone group had a greater rate of reduction of CSF cytokines (TNF, IL-1, IL-12 and IFN-ɣ) (Extended Data Table 3 and Supplementary Fig. 19a) and all five CSF inflammatory pathways than the placebo group (Extended Data Table 4 and Supplementary Fig. 20a); whereas in CT-genotype participants, although dexamethasone was associated with faster reductions in CSF TNF, IL-1 and IL-12 than placebo (Extended Data Table 5 and Supplementary Fig. 19b), it did not induce faster resolution of any of the CSF inflammatory pathways (Extended Data Table 6 and Supplementary Fig. 20b).

In whole blood, dexamethasone was associated with significant suppression of the transcriptional activity of IFN and cytokine signaling pathways compared with placebo in all CC- and CT-genotype participants (Extended Data Table 7 and Supplementary Fig. 21). However, this effect was driven primarily by CT-genotype participants, with no significant dexamethasone effect in CC-genotype participants (Extended Data Tables 8 and 9 and Supplementary Fig. 22).

### Safety

In CC- and CT-genotype participants, at least one serious adverse event occurred in 161/305 (52.8%) dexamethasone-treated and 160/308 (51.9%) placebo-treated participants (*P* value for difference = 0.84) (Extended Data Table 10 and Supplementary Tables 44 and 45), with no difference in event frequency between genotypes (Supplementary Tables 44–50). Likewise, grade 3 and 4 adverse events frequency was similar between treatment arms and genotypes (Supplementary Table 51).

### Exploratory analyses

In an exploratory analysis, CSF cytokines (Supplementary Fig. 23) and CSF inflammatory pathways (Supplementary Fig. 24) at day 30 were not significantly different between participants of CC and CT genotype. Further exploratory analyses studied the association of day 30 CSF cytokines and CSF inflammatory pathways, with the trial’s primary outcome, both unadjusted and adjusted for *LTA4H* genotype, dexamethasone/placebo allocation and baseline CSF cytokines. In the unadjusted analysis of CSF cytokines, individuals who had survived until day 30 and then subsequently died or experienced a new neurological event (the primary outcome) had significantly higher concentrations of CSF TNF, IL-1β, IL-6, IFN-γ and IL-10, than individuals who had survived until day 30 and subsequently did not meet the trial’s primary outcome (Supplementary Table 52). This CSF cytokine association was not seen in the adjusted analysis. In both unadjusted and adjusted analyses, the enrichment scores of six CSF inflammatory pathways (IFN signaling, neutrophil degranulation, neutrophil activation, eicosanoid metabolic process, TNF signaling and cytokine signaling) were increased in individuals who had survived until day 30 and then subsequently died or experienced a new neurological event, versus individuals who had survived until day 30 and subsequently did not meet the trial’s primary outcome.

### Planned individual participant meta-analysis

In 2004, we and others reported the results of a randomized comparison of dexamethasone versus placebo for the treatment HIV-negative and HIV-positive Vietnamese adults with tuberculous meningitis^8^. The trial was conducted in the same centers in Ho Chi Minh city as the current trial, with the same intervention (dose, duration and route of administration of dexamethasone). The only differences in methodology between the 2004 and the current trial were that in 2004, participants were included following up to 30 days antituberculosis treatment (rather than up to 6 days), and antituberculosis treatment duration and follow-up was for 9 months (rather than 12 months).

Given the similarities between the trials, we conducted a planned individual participant meta-analysis that combined 702 participants from LAST ACT (including TT-genotype dexamethasone-treated participants) with 447 HIV-negative Vietnamese adults with tuberculous meningitis from the 2004 trial (all three genotypes included)^8^. Baseline characteristics of 1,149 combined trial participants (525 placebo, 624 dexamethasone) showed that the current trial participants were more likely to have definite tuberculous meningitis (312/702 (44.4%) versus 97/447 (21.7%)) and less severe disease (298/702 (42.5%) modified MRC severity grade 1 versus 151/447 (33.8%)) than the 2004 trial^8^ (Supplementary Table 53). Overall, death occurred in 135/624 (21.6%) given dexamethasone and 138/525 (26.3%) given placebo (hazard ratio of 0.78, 95% CI 0.62–0.99; *P* = 0.044; RMTL difference 0.42 months, 95% CI 0.04–0.79; *P* = 0.023) (Supplementary Fig. 25 and Supplementary Table 54). Nine-month death or disability was similar between treatment arms (267/613 (43.6%) dexamethasone versus 236/514 (45.9%) placebo, *P* = 0.4). An exploratory analysis combining all placebo-treated participants, and all dexamethasone-treated participants, from the current trial and the 2004 trial^8^, showed mortality was lower in the current trial versus the 2004 trial (*P* < 0.001 in placebo-treated participants, *P* = 0.12 in dexamethasone-treated participants) (Supplementary Fig. 25).

## Discussion

Ever since the advent of antituberculosis chemotherapy, physicians have recognized that some patients with tuberculosis suffer worsening inflammation and symptoms after starting treatment^15^. Such inflammation can have fatal consequences in tuberculous meningitis, thus adjunctive corticosteroids have been advocated in treatment since the 1950s^16^. Yet, determining who with tuberculous meningitis benefits from corticosteroids and who does not has proven difficult. Clinical trials suggest modest survival benefit in HIV-negative adults and children^8,14,17^ and, more recently, uncertain benefit in HIV-positive individuals^18^.

Studies conducted in Vietnam suggested that *LTA4H*-genotype influences intracerebral inflammation and dexamethasone-induced survival benefit^7,12,13,19^. Our current trial sought to extend these observations and determine if *LTA4H* genotype can predict dexamethasone’s benefit in HIV-negative adults with tuberculous meningitis. We found that in CC- and CT-genotype, or CC-genotype participants alone, placebo was not noninferior to dexamethasone with respect to the primary outcome of death or new neurological event by 12 months from randomization. However, neither was dexamethasone associated with significant improvement in the primary or any secondary outcomes or in any prespecified subgroups. Overall, outcomes in dexamethasone-treated TT-genotype participants were similar to CC- and CT-genotype participants, although TT genotype had more severe disease. An exploratory analysis (Supplementary Fig. 6) suggested that the differential effects of dexamethasone associated with *LTA4H* genotype may be most pronounced in those with severe (modified MRC severity grade 3) disease, with substantially better outcomes in dexamethasone-treated TT- than CC- and CT-genotype participants.

A planned individual participant data meta-analysis of 1,149 adults from the current and previous trial^8^, conducted in Vietnamese adults from the same hospitals with the same intervention, suggested that dexamethasone reduced overall mortality, regardless of *LTA4H* genotype. However, this analysis must be interpreted cautiously. Current trial participants had less severe disease, more definite diagnoses, and placebo-treated participants had significantly lower mortality than the earlier trial. These differences, possibly caused by better diagnostics (for example, GeneXpert Ultra) and improvements in clinical care, may have reduced dexamethasone’s beneficial effects in the current trial and introduced bias. In addition, there were no placebo-treated TT-genotype participants in the current trial, which means we were unable to confirm the previous and substantial benefit of dexamethasone in this population.

We sought to understand the differential effects of dexamethasone between *LTA4H* genotypes through a planned analysis of CSF and systemic inflammation. Unlike our previous investigations^7^, TT genotypes did not have significantly higher baseline CSF cytokine concentrations or systemic inflammation measures than CC and CT genotypes. We observed, however, stronger suppression of CSF cytokines and inflammatory pathways in CC-genotype dexamethasone-treated participants and minimal suppression in CT-genotype participants. Linked to these findings were possible improvements in outcome in dexamethasone-treated CC-genotype participants, negating previous concerns that CC-genotype patients might be harmed by dexamethasone^12^.

These findings suggest *LTA4H* genotype influence pathophysiology and corticosteroid response, although they do not conform to earlier models^7^. Our data do not provide any definitive explanations for the discrepancy between our past and current findings. It may reflect the limitations of previous post-hoc analysis of trials and observational studies, which may have over-estimated the effects of *LTA4H* on pathophysiology and treatment outcomes. In addition, it suggests the relationships between host genomic variation, inflammation, dexamethasone treatment and outcome are complex and remain poorly understood. Other genetic variants and factors may also associate with dexamethasone-responsive inflammation in tuberculous meningitis. For example, we recently observed that the association between higher CSF cytokine levels and survival extended to non-TT-genotype patients^19^ strongly suggesting there are other influences on tuberculous meningitis pathophysiology.

Our trial has some limitations. First, it was conducted exclusively in Vietnam. *LTA4H* genotype frequency and effects vary between populations: TT genotype represents <5% of some African populations^20^, and links with tuberculosis phenotypes and treatment responses have varied substantially across populations^20–23^. Studies conducted in Indonesia have suggested *LTA4H* genotype may influence inflammation but not outcome from tuberculous meningitis^24^, although a more recent Bayesian analysis of Vietnam and Indonesian data suggested a possible survival benefit in dexamethasone-treated TT-genotype Indonesian patients in those with less severe disease^13^. Ongoing tuberculous meningitis trials in Africa and Asia^25^, in which all participants receive corticosteroids, present an opportunity to determine the wider impact of *LTA4H* genotype on corticosteroid-associated survival. Second, we were only able to assess systemic and intracerebral inflammation in a subset of trial participants, although these appeared representative of the full trial population.

The strengths of the trial include its relatively large size, powered for clinically important endpoints of death and new neurological events and the careful characterization of treatment response, including inflammation resolution, through 12 months of follow-up. Furthermore, to the best of our knowledge, this is the first host genotype stratified randomized controlled trial ever conducted for tuberculosis treatment.

In conclusion, our trial did not clearly establish noninferiority of placebo in HIV-negative *LTA4H* CC- and CT-genotype Vietnamese adults with tuberculous meningitis. However, nor did it provide evidence that dexamethasone was either beneficial or harmful in this population. The effects of *LTA4H* genotype on pre-treatment inflammation and dexamethasone-responsiveness did not conform to previous data, although there was some evidence TT-genotype individuals may have selective benefit of dexamethasone, especially in those with severe disease. Importantly, there was no evidence of harm in dexamethasone-treated CC- and CT-genotype participants. Analysis of the combined data from this and the largest previous trial of dexamethasone supports dexamethasone’s modest survival benefit in all HIV-negative adults with tuberculous meningitis, although the results indicate the importance of early diagnosis and treatment to good outcomes.

Taken together, our findings support dexamethasone’s continued use in HIV-negative adults with tuberculous meningitis, regardless of *LTA4H* genotype and despite modest benefits. While the potential benefit of dexamethasone in TT-genotype individuals with severe disease requires further study, *LTA4H* genotyping does not currently provide information that can influence clinical management. The modest and heterogeneous benefit of dexamethasone calls for greater understanding of tuberculous meningitis pathophysiology across different geographic populations and better targeted more effective anti-inflammatory agents than corticosteroids.

## Methods

The trial methodology, conduct and analysis are described in the published protocol^27^ and statistical analysis plan^28^. The trial was designed and delivered by the investigators, supported by the Oxford University Clinical Research Unit (OUCRU) Clinical Trials Unit. All authors vouch for the data and analysis. The trial was registered at ClinicalTrials.gov (NCT03100786).

### Settings and study population

We recruited participants from the Hospital for Tropical Diseases and Pham Ngoc Thach Hospital for Tuberculosis and Lung Disease, in Ho Chi Minh City, Vietnam.

Individuals were considered eligible for enrollment if all inclusion criteria were met. As detailed in the published protocol^27^ inclusion criteria for the LAST ACT trial were:

1. ≥18 years old
2. HIV negative
3. A clinical diagnosis of tuberculous meningitis ( ≥5 days of meningitis symptoms and consistent CSF abnormalities) with antituberculosis chemotherapy either planned or started by the treating clinician

Individuals were considered ineligible for enrollment if any exclusion criteria were met. As detailed in the published protocol^27^ exclusion criteria for the LAST ACT trial were:

1. An additional brain infection (other than tuberculous meningitis) confirmed or suspected: positive CSF Gram or India Ink stain, positive blood or CSF Cryptococcal antigen test and cerebral toxoplasmosis suspected and attending clinician plans to give antitoxoplasmosis treatment with antituberculosis treatment.
2. More than six consecutive days of two or more drugs active against *M. tuberculosis* immediately before screening.
3. More than six consecutive days of any type of orally or intravenously administered corticosteroid immediately before randomization.
4. Systemic corticosteroids were considered mandatory for any reason by the attending clinician.
5. Systemic corticosteroids were considered contraindicated for any reason by the attending clinician.
6. Patient previously been randomized into the LAST ACT trial for a prior episode of tuberculous meningitis.
7. Lack of consent from the participant or family member (if the participant is incapacitated by the disease).

Participants were subsequently classified as having definite, probable, or possible tuberculous meningitis, following published diagnostic criteria^26^ (Supplementary Table 55).

### Study oversight

Written informed consent to enter the trial was obtained from all participants or a relative if they were incapacitated. If capacity returned, consent from the participant was obtained. Trial approvals were obtained from local and national ethics and regulatory authorities in Vietnam, and the Oxford Tropical Research Ethics Committee in the UK (Supplementary Text 1). An independent data monitoring committee (Supplementary Text 2) reviewed data, following a prespecified plan, at 6-monthly intervals for the first 24 months of enrollment and annually thereafter.

### *LTA4H* genotyping

Once a participant or their relative consented to enter the study, blood was taken for rapid *LTA4H* genotyping. Genomic DNA was extracted from 5 ml of venous blood collected in tubes containing EDTA anti-coagulant using the Nucleon BACC2 Genomic DNA extraction kits (GE Healthcare). DNA quality and concentration were determined by Nanodrop 2000 v1.0 Spectrophotometer (Thermo Scientific). rs17525495 was genotyped by Taqman using predesigned assay kits (Applied Biosystems) according to the supplier’s instructions (https://documents.thermofisher.com/TFS-Assets/LSG/manuals/MAN0009593_TaqManSNP_UG.pdf). We performed real-time PCR with 15 ng of DNA using a LightCycler 480 Probes Master kit on the LightCycler 480 real-time PCR system (Roche) according to the supplier’s protocol (https://pim-eservices.roche.com/LifeScience/Document/4dd0e207-97ed-e311-98a1-00215a9b0ba8). For each PCR run, the DNA samples from each participant were analyzed in duplicate, and three positive control samples for TT, CC homozygous and CT heterozygous were included. These control samples were from samples that had been previously genotyped for rs17525495 by Taqman, as reported elsewhere^29^ and confirmed by Sanger sequencing. The genotyping results were then analyzed using LightCycler480 software.

### Randomization and study groups

Randomization occurred once *LTA4H* genotyping results were available, usually within 24 h. *LTA4H* CC- and CT-genotype participants were randomized to two parallel groups in a 1:1 ratio: dexamethasone or placebo for 6–8 weeks. TT-genotype participants received open-label dexamethasone for 6–8 weeks.

Randomization was stratified by participating hospital, *LTA4H* genotype and modified MRC disease severity grade^30^ assessed at enrollment. Participants in grade I had a Glasgow coma score of 15 (possible range 3–15, with higher scores indicating better status) with no focal neurologic signs, grade II participants had a score of either 11–14 or had focal neurological signs and grade III participants had a score of 10 or less. The randomization list was computer-generated based on random permuted blocks with block size 4 and 6 (probability 0.75 and 0.25). Participant randomization was performed by trained clinical staff using a web-based software, with 24-h availability.

### Study treatments

All participants received standard-of-care antituberculosis chemotherapy according to national guidelines. Rifampicin (10 mg kg^−1^ per 24 h, maximum 600 mg), isoniazid (5 mg kg^−1^ per 24 h, maximum 300 mg), pyrazinamide (25 mg kg^−1^ 24 h, maximum 2 g) and ethambutol (20 mg kg^−1^ per 24 h, maximum 1.2 g) were given for at least the first 2 months of treatment, provided drug resistance was not suspected or proven. Pyrazinamide was then stopped and rifampicin, isoniazid and ethambutol (at the same doses) were given until at least 12 months antituberculosis treatment in total had been given. Patients with visual complications discontinued ethambutol and an alternative drug was used in its place. The decision of which alternative fourth drug to use is made by the treating physician and was not defined by the trial, but in practice was either levofloxacin or streptomycin or amikacin.

For tuberculous meningitis caused by isoniazid-resistant tuberculosis the attending physician decided which drugs to prescribe, dependent upon clinical circumstances, but usually isoniazid was substituted by levofloxacin. For participants with multidrug resistant tuberculosis, second-line treatment was given as soon as possible, following national guidelines and local policies.

Participants allocated dexamethasone received the 6–8-week regimen previously shown to reduce tuberculous meningitis mortality^8^. Patients with grade II or III disease received intravenous treatment for four weeks (0.4 mg per kilogram per day for week 1, 0.3 mg per kilogram per day for week 2, 0.2 mg per kilogram per day for week 3 and 0.1 mg per kilogram per day for week 4) and then oral treatment for 4 weeks, starting at a total of 4 mg per day and decreasing by 1 mg each week. Patients with grade I disease received three weeks of intravenous therapy (0.3 mg per kilogram per day for week 1, 0.2 mg per kilogram per day for week 2 and 0.1 mg per kilogram per day for week 3) and then 3 weeks of oral therapy, starting at a total of 3 mg per day and decreasing by 1 mg each week. We followed this drug dosage regimen, as it was the one which proved beneficial to adults in our previous trial^8^. No other corticosteroid regimens, including oral regimens, are supported by such strong trial data; therefore, we could not justify using alternative drugs or doses. For our current trial, patients with grade I disease received intravenous therapy during week 3, whereas in our previous trial^8^, patients with grade I disease received oral therapy during week 3 (albeit at 0.1 mg per kilogram per day in both trials). For the trial published in the year 2004^8^, we chose to have a shorter regimen for the least severe patients (grade 1) for pragmatic reasons: at the time doctors felt those with less severe disease were usually discharged home within 3 weeks and therefore it was not acceptable to have a 4-week intravenous regimen. Individuals with more severe disease usually stayed in hospital for longer, and longer intravenous treatment was both desirable and possible. This regimen is now the global standard-of-care.

Blinded fully made-up and labeled study treatment packs contained either dexamethasone or identical placebo. All participants and investigators were blinded to study drug allocation. Adherence to medication was ensured with the use of supervised drug intake for inpatients, encouraged by detailed instructions at discharge, and medication compliance checks at follow-up visits or phone calls.

### Outcome assessments

The primary endpoint was all-cause mortality or new neurological event during 12 months from randomization. A new neurological event was defined as a Glasgow coma score reduction by ≥2 points for ≥2 days from the highest previous score or the new onset of cerebellar symptoms, focal neurological signs or seizures.

Secondary endpoints, assessed over 12 months from randomization, were overall mortality, first new neurological event, neurological disability (modified Rankin score 3–5) (Supplementary Table 56), modified Rankin score as an ordinal scale, use of open-label corticosteroid treatment for any reason, serious or severe adverse events, and resolution of CSF and blood inflammation. The Rankin scale assesses dependence. A score of 0 indicated no symptoms; 1 indicated minor symptoms not interfering with lifestyle; 2 indicated symptoms that might restrict lifestyle, but patients could look after themselves; 3 indicated symptoms that restricted lifestyle and prevented independent living; 4 indicated symptoms that prevented independent living, although constant care and attention were not required; and 5 indicated total dependence on others, requiring help day and night.

### Clinical assessments

Participants underwent clinical assessments at baseline—days 3, 7, 10, 14, 21 and 30—and monthly until month 12. Assessment included Glasgow coma score, focal neurological deficits and details of adverse events. Participants were monitored daily whilst in hospital and serious adverse events were reported to local and national regulators. In participants for whom systemic corticosteroids were considered necessary by the treating clinician after randomization, study drug was discontinued (with previous doses remaining blinded) and corticosteroids commenced.

### CSF and whole blood analyses

Lumbar CSF was sampled at baseline and on days 30 and 60. At least 6 ml of CSF (if available) was used for Ziehl–Neelsen smear microscopy, either Xpert MTB/RIF or Xpert MTB/RIF Ultra, and mycobacterial culture (mycobacteria growth indicator tube) following standard procedures^31^. Phenotypic drug susceptibility testing was performed using a BACTEC mycobacteria growth indicator tube SIRE kit (Becton, Dickinson).

Preplanned analyses of CSF proteomic and whole blood transcriptomic data were performed, focused on six targeted pathways known to be important mediators of tuberculous meningitis (TBM) pathogenesis: CSF IFN signaling, neutrophil degranulation, neutrophil activation, eicosanoid metabolic processes, TNF signaling, and cytokine signaling, and ten reported cytokines. CSF inflammatory proteins were measured using the Olink Explore 384 Inflammation Panel (Olink Proteomics). Olink measurements were conducted for 675 participants on day 0 (*n* = 675) and day 30 (*n* = 397) at the Human Genomics Facility of the Genetic Laboratory, Department of Internal Medicine, Erasmus MC. Raw protein expression data were reported as normalized protein expression units, which were log_2_-transformed and normalized using the plate control method to minimize technical variation. To correct for batch effects, the normalized protein expression values then were further adjusted using the ComBat function from the sva R package^32^. Quality control procedures were conducted at both the sample and protein levels. At the sample level, poor-quality samples were excluded if they exhibited a failure rate of ≥50% across protein assays, as determined by Olink’s internal quality control criteria. Outliers were identified via principal component analysis and excluded if they deviated by more than three standard deviations from the first principal component. At the protein level, proteins with a limit of detection exceeding 75% of samples were filtered out. Finally, 17 participants who died before randomization were excluded, resulting in a final dataset of 275 proteins in 1,029 CSF samples from 646 participants available for analysis (day 0: *n* = 638; day 30: *n* = 391). Among the ten planned CSF cytokines—TNF, IL-1β, IL-2, IL-6, IL-12β, IFN-γ, IL-4, IL-5, IL-10 and IL-13—four (IL-2, IL-4, IL-5 and IL-13) did not pass quality control due to their limit of detection exceeding 75% of samples.

Whole-blood RNA sequencing was performed for the first 207 consecutively enrolled participants on day 0 (*n* = 207), day 14 (*n* = 191) and day 60 (*n* = 156). Whole-blood samples were preserved in PAXgene Blood RNA collection tubes at −80 °C. Total RNA was subsequently extracted using the PAXgene Blood RNA Kit (Qiagen, Valencia), following the manufacturer’s protocol. Extracted RNA was shipped to the Ramaciotti Centre for Genomics (University of New South Wales) for high-throughput sequencing. Library preparation was performed using the TruSeq Stranded Total RNA with Ribo-Zero Globin kit (Illumina) to deplete globin transcripts and ribosomal RNA. Sequencing was conducted on the Illumina NovaSeq 6000 platform, generating approximately 30 million 100 bp paired-end reads per sample. Raw sequencing data were subjected to quality control and aligned to the human reference genome (GRCh38 build 99) with the STAR aligner (v2.5.2a)^33^. Gene-level quantification from aligned reads was performed using FeatureCounts (v2.0.0), generating raw counts for 60,067 genes^34^. Before analysis, five participants were excluded: two who died before randomization and three whose RNA sequencing data were poor quality (RNA integrity number <4 and uniquely mapped reads <10 million). This resulted in a final sequencing dataset of 202 participants (day 0: *n* = 202, day 14: *n* = 188, day 60: *n* = 153). To further clean the dataset, hemoglobin genes, ribosomal RNA genes and genes with low expression (median count <10) were filtered out, reducing the gene set to 20,533. Gene expression values were then normalized and log_2_-transformed using the variance stabilizing transformation algorithm implemented in the DESeq2 package in R (v1.34.0) to enable downstream statistical analyses^35^. For each targeted pathway, a single sample enrichment score was calculated using the *z*-score method to evaluate the activity of the pathway at each timepoint for both whole blood transcriptomics and CSF proteomics, for each patient.

### Statistical analyses

We adopted a hybrid trial-design approach that aimed to prove noninferiority of placebo first but also superiority of placebo should dexamethasone prove harmful. The trial had two primary populations: the combined CC- and CT-genotype population and the CC-genotype population^28^.

The primary analysis used a Cox proportional hazards regression model with the primary endpoint as the outcome, where we aimed to prove noninferiority of placebo in the CC- and CT-genotype population or the CC-genotype subgroup. We corrected for multiple testing using the method by Spiessens and Debois^36^. It takes the correlation into account between the test statistic for the CC-genotype subgroup analysis and the overall CC- and CT-genotype analysis, which makes it less conservative than the Bonferroni approach. We chose to spend 2% of the one-sided type I error of 2.5% to the CC- and CT-genotype analysis, leaving 0.86% for the CC-genotype subgroup analysis.

In addition, we evaluated the primary endpoint using a superiority design, in the CC- and CT-genotype population, in the CC-genotype subgroup and in the CT-genotype subgroup. In the superiority analyses, we did not make any correction for multiple testing.

We set the noninferiority margin in favor of dexamethasone at a hazard ratio of 0.75 and assumed a true hazard ratio of 1.15 in the CC- and CT-genotype population. To obtain 80% power at the one-sided 2% significance level, 184 events in the CC- and CT-genotype population would be required. Assuming a 12-month risk of the primary endpoint in the dexamethasone arm of 35%, a hazard ratio of 1.15 corresponds to a risk of 31.2% of placebo, and the noninferiority margin implies that we can exclude an absolute risk increase of placebo of (at worst) +8.7%. Assuming an overall event risk of ≥32% and 11% sample increase to compensate for loss-to-follow-up, we aimed to randomize 640 CC- and CT-genotype participants. Anticipating 10% being *LTA4H* TT genotype, we planned to enroll 720 participants in total.

The analysis followed a prespecified and published plan^28^. The data were analyzed using the program R (version 4.4.2; R Core Team, 2024)^37^. In brief, intention-to-treat and per-protocol analyses were performed for the primary and secondary endpoints. The intention-to-treat analysis included all randomized CC- and CT-genotype participants, even if no study drug was received after randomization. The per-protocol analysis included all randomized participants, excluding those subsequently found to have not met all inclusion criteria or to have met any exclusion criteria and participants with a final diagnosis other than tuberculous meningitis. We also excluded participants who received <7 days of study drug or <30 days of antituberculosis drugs, for any reason other than death. Participants who were enrolled (genotyped) but not randomized were not included in either analysis. Sex and gender were not considered in the study design; while males are more commonly affected, tuberculous meningitis pathophysiology is not known to vary by sex. Sex-based analyses were not prespecified and not reported here. Sex was determined based on self-reporting. Gender data were not also collected.

Baseline characteristics were summarized by treatment arm and genotype for intention-to-treat and per-protocol analyses. The primary analysis was a Cox proportional hazards regression model with the primary endpoint as the outcome, treatment as the only covariate and with *LTA4H* genotype (CC or CT) and modified MRC severity grade at enrollment as stratum variables. We also performed the analysis in CC-genotype participants only. The null hypothesis of the noninferiority comparison was tested via CIs for the hazard ratio. Further analyses used a superiority design, using hazard ratio and difference in RMTL as effect measures for the time-to-event outcomes and logistic regression and a proportional odds regression model for modified Rankin scores. For the Rankin score *R* = *r*, where 1 ≤ *r* ≤ 6, the proportional odds regression model quantifies the odds of having a score of *r* or higher for a participant allocated to dexamethasone, compared with a participant allocated to placebo, that is, (Probability(*R* ≥ *r*|Dexamethasone)/Probability(*R* < *r*|Dexamethasone))/(Probability(*R* ≥ *r*|Placebo)/Probability(*R* < *r*|Placebo)). In the regression equation we allow the intercept to differ by *r*, whereas the relation of the covariables with the odds is assumed not to depend on *r*.

Subgroup analysis of the primary endpoint was planned for *LTA4H* genotype, and enrollment modified MRC severity grade, tuberculous meningitis diagnosis (definite, probable and possible) and *M. tuberculosis* drug susceptibility profile. Analyses were conducted for CC and CT genotypes combined and CC genotype alone, with the exception of adverse events, which were compared by genotype. No corrections were made for multiple testing, except for the CSF inflammation and blood analysis that focused on predefined pathways and molecules of known importance to tuberculous meningitis pathogenesis^38^. Linear mixed-effects models and Bayesian approaches described *LTA4H* genotype effects on baseline transcriptomic and proteomic signatures and the longitudinal effects of treatment and genotype upon them.

We compared TT-genotype participants with dexamethasone- and placebo-treated CC- and CT-genotype participants for mortality, new neurological events, disability and CSF/blood inflammation. An individual participant data meta-analysis was planned to investigate dexamethasone’s effect on survival and disability in HIV-negative adults with TBM overall, independent of *LTA4H* genotype^28^. Current trial data were combined with 447 HIV-negative adults with tuberculous meningitis from the 2004 trial^8^.

Some exploratory analyses (not articulated in the statistical analysis plan) were conducted and presented. These include the effect of MRC grade on dexamethasone’s effect on survival across the three genotypes (Supplementary Fig. 6); a comparison of day 30 CSF cytokines and CSF inflammatory pathways between participants of CC and CT genotype (Supplementary Figs. 23 and 24); the association of day 30 CSF cytokines and CSF inflammatory pathways with the trial’s primary outcome (Supplementary Table 52); the effect of MRC grade on dexamethasone survival effect in the combined data from the two trials (Supplementary Fig. 25b); and a comparison of 9-month survival between the two trials (Supplementary Fig. 25c).

### Protocol amendments

Major amendments made to the LAST ACT protocol after study start, relevant to these results, are:

1. A change in an exclusion criterion to allow no more than 6 days of any type of orally or intravenously administered corticosteroid immediately before enrollment (previously no more than 3 days).
2. Randomization to be stratified by tuberculous meningitis grade with grade set at enrollment, even if this grade had changed by randomization (which can only be performed once *LTA4H* genotyping results are available).
3. Serum potassium to be checked and recorded at baseline. Values to be recorded if they are repeated as part of routine clinical care.
4. Following discharge, participants to be followed up either in the hospital’s outpatient department or via a phone call. In-person or phone call outpatient review to occur monthly (±7 days) for at least the first 2 months following hospital discharge. If a patient is discharged at day 21 (for example, a grade 1 patient after 3 weeks of intravenous study drug), they are not required to return for follow up at day 30 (month 1). Disability questions due to be asked at day 30 will instead be asked at day 21.

### These protocol amendments were approved by all necessary ethical committees

#### Protocol deviations

Major deviations from the LAST ACT protocol after study start, relevant to these results, are as follows: nine participants were discharged from hospital while still within the intravenous study drug period (in eight cases, this was done in accordance with the participant’s wishes). In each case, intravenous study drug was given as per schedule up to the point of early discharge. The oral study drug period was then started at discharge. The remaining days of intravenous study drug were not replaced, resulting in a shorter total duration of study drug than originally scheduled. Details of participants with shortened study drug regimens, in order of enrollment, are as follows:

1. The participant received a complete week of 0.4 mg kg^−1^ intravenously and 4 days of 0.3 mg kg^−1^ intravenously, followed by completion of the scheduled oral study drug.
2. The participant received a complete week of 0.4 mg kg^−1^ intravenously and 5 days of 0.3 mg kg^−1^ intravenously, followed by completion of the scheduled oral study drug.
3. The participant received a complete week of 0.4 mg kg^−1^ intravenously and 3 days of 0.3 mg kg^−1^ intravenously, followed by completion of the scheduled oral study drug.
4. The participant received a complete week of 0.4 mg kg^−1^ intravenously, a complete week of 0.3 mg kg^−1^ intravenously, a complete week of 0.2 mg kg^−1^ intravenously and 2 days of 0.1 mg kg^−1^ intravenously, followed by completion of the scheduled oral study drug.
5. The participant received a complete week of 0.4 mg kg^−1^ intravenously and 5 days of 0.3 mg kg^−1^ intravenously, followed by completion of the scheduled oral study drug.
6. The participant received a complete week of 0.4 mg kg^−1^ intravenously, a complete week of 0.3 mg kg^−1^ intravenously, a complete week of 0.2 mg kg^−1^ intravenously and 5 days of 0.1 mg kg^−1^ intravenously, followed by completion of the scheduled oral study drug.
7. The participant received a complete week of 0.4 mg kg^−1^ intravenously, a complete week of 0.3 mg kg^−1^ intravenously and 5 days of 0.2 mg kg^−1^ intravenously, followed by completion of the scheduled oral study drug.
8. The participant received a complete week of 0.4 mg kg^−1^ intravenously, a complete week of 0.3 mg kg^−1^ intravenously and 2 days of 0.2 mg kg^−1^ intravenously, followed by completion of the scheduled oral study drug.
9. The participant received a complete week of 0.4 mg kg^−1^ intravenously and 5 days of 0.3 mg kg^−1^ intravenously, followed by completion of the scheduled oral study drug.

### Ethics and inclusion statement

Our trial was designed and conducted by researchers and clinicians, all of whom were living and working within Vietnam. We included local researchers and clinicians in the study design, study implementation and authorship of this publication. Local researchers gave input into study protocol design including the schedule of study investigations and performed recruitment of trial participants routine clinical care and study procedures. Contributions of local researchers to authorship are detailed in the ‘Author contributions’ section and include: data curation, formal analysis, investigation, methodology, resources, software, supervision and visualization. The research is locally relevant and was determined in collaboration with local partners. Tuberculous meningitis is the most severe form of tuberculosis and is regularly encountered in the study setting. Interventions that could optimize therapy and potentially reduce poor clinical outcomes in this disease are locally relevant and would benefit the communities involved in this study. Roles and responsibilities were agreed amongst collaborators ahead of the research, and capacity-building plans for local researchers were discussed. Local clinical, laboratory and researcher staff were trained in the conduct of high quality clinical trial research, building capacity for future studies at these sites. Trial approvals were obtained from local and national ethics and regulatory authorities in Vietnam, and the Oxford Tropical Research Ethics Committee in the UK. Ethical approvals were as follows: The Oxford Tropical Research Ethics Committee (approval no. 52-16), The Ethical Committee of the Hospital for Tropical Diseases (approval no. 37/HDDD), The Ethical Committee of Pham Ngoc Thach Hospital for Tuberculosis and Lung Disease (approval no. 1034/HDDD-PNT) and The Vietnam Ministry of Health (approval no. 151/CN-BDGDD). Local and regional research relevant to this study has been considered in citations. In line with a reimbursement standard operating procedure at OUCRU, participants (and one relative to attend follow-up visits) received reimbursement for travel expenses.

### Reporting summary

Further information on research design is available in the Nature Portfolio Reporting Summary linked to this article.

## Online content

Any methods, additional references, Nature Portfolio reporting summaries, source data, extended data, supplementary information, acknowledgements, peer review information; details of author contributions and competing interests; and statements of data and code availability are available at 10.1038/s41591-025-04138-z.

## Supplementary information

Supplementary Information Supplementary Text 1 and 2 (this includes a list of LAST-ACT investigators), Figs. 1–26 and Tables 1–56.

Reporting Summary

## Figures and Tables

**Extended Data Table 1:**
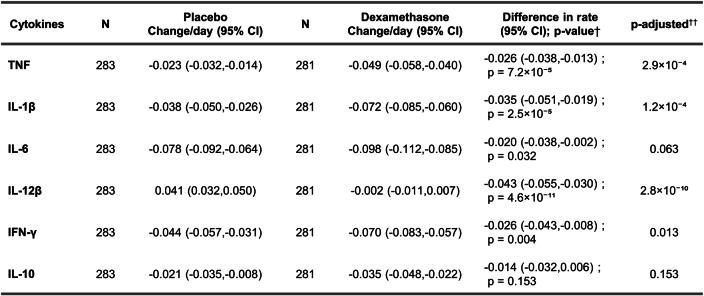
Changes in CSF cytokines concentrations from day 0 to 30 in CC- and CT-genotype participants

95% CI: Bayesian 95% credible intervals.

†p-value: Derived from crude “Wald-type” tests, calculated as the posterior mean estimate divided by its standard deviation from the MCMC sampling of coefficients in the joint model. All tests were two-sided.

^††^p-values adjusted for multiple comparisons using the Hochberg procedure. CI=confidence interval. CSF=cerebrospinal fluid. IFN=interferon. IL=interleukin. TNF=tumour necrosis factor.

**Extended Data Table 2:**
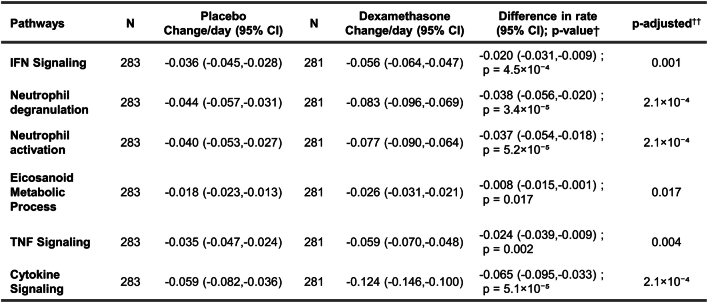
Changes in CSF pathogenesis pathways from day 0 to 30 in CC- and CT-genotype participants

95% CI: Bayesian 95% credible intervals.

†p-value: Derived from crude “Wald-type” tests, calculated as the posterior mean estimate divided by its standard deviation from the MCMC sampling of coefficients in the joint model. All tests were two-sided.

^††^p-values adjusted for multiple comparisons using the Hochberg procedure. CI=confidence interval. CSF=cerebrospinal fluid. IFN=interferon. IL=interleukin. TNF=tumour necrosis factor.

**Extended Data Table 3:**
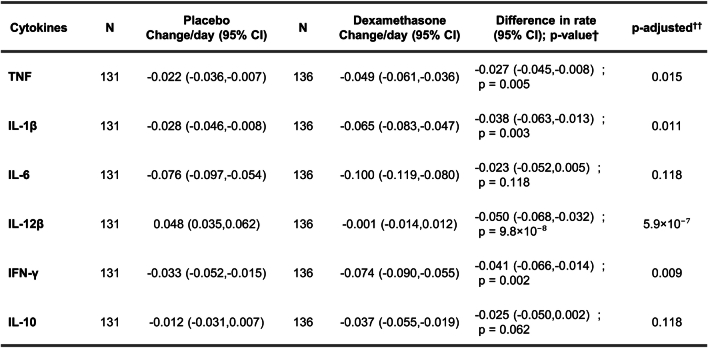
Changes in CSF cytokines concentrations from day 0 to 30 in CC-genotype

95% CI: Bayesian 95% credible intervals.

†p-value: Derived from crude “Wald-type” tests, calculated as the posterior mean estimate divided by its standard deviation from the MCMC sampling of coefficients in the joint model. All tests were two-sided.

^††^p-values adjusted for multiple comparisons using the Hochberg procedure. CI=confidence interval. CSF=cerebrospinal fluid. IFN=interferon. IL=interleukin. TNF=tumour necrosis factor.

**Extended Data Table 4:**
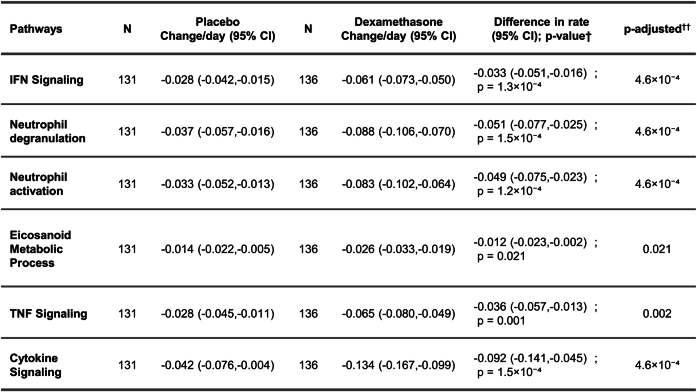
Changes in CSF pathogenesis pathways from day 0 to 30 in CC-genotype participants

95% CI: Bayesian 95% credible intervals.

†p-value: Derived from crude “Wald-type” tests, calculated as the posterior mean estimate divided by its standard deviation from the MCMC sampling of coefficients in the joint model. All tests were two-sided.

^††^p-values adjusted for multiple comparisons using the Hochberg procedure.CI=confidence interval. CSF=cerebrospinal fluid. IFN=interferon. TNF=tumour necrosis factor.

**Extended Data Table 5:**
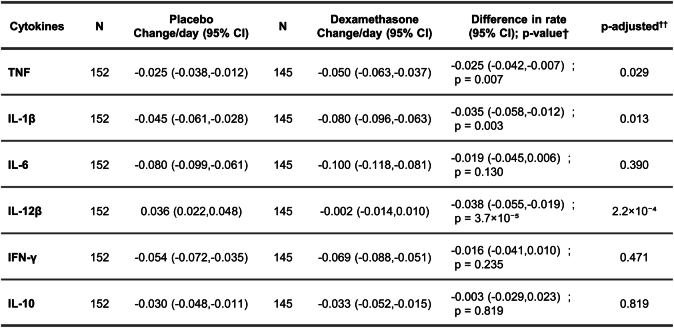
Changes in CSF cytokines concentrations from day 0 to 30 in CT-genotype participants

95% CI: Bayesian 95% credible intervals.

†p-value: Derived from crude “Wald-type” tests, calculated as the posterior mean estimate divided by its standard deviation from the MCMC sampling of coefficients in the joint model. All tests were two-sided.

^††^p-values adjusted for multiple comparisons using the Hochberg procedure. CI=confidence interval. CSF=cerebrospinal fluid. IFN=interferon. IL=interleukin. TNF=tumour necrosis factor.

**Extended Data Table 6:**
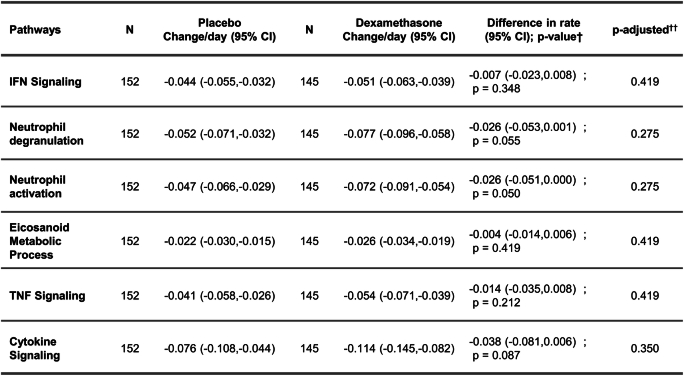
Changes in CSF pathogenesis pathways from day 0 to 30 in CT-genotype participants

95% CI: Bayesian 95% credible intervals.

†p-value: Derived from crude “Wald-type” tests, calculated as the posterior mean estimate divided by its standard deviation from the MCMC sampling of coefficients in the joint model. All tests were two-sided.

^††^p-values adjusted for multiple comparisons using the Hochberg procedure. CI=confidence interval. CSF=cerebrospinal fluid. IFN=interferon. TNF=tumour necrosis factor.

**Extended Data Table 7:**
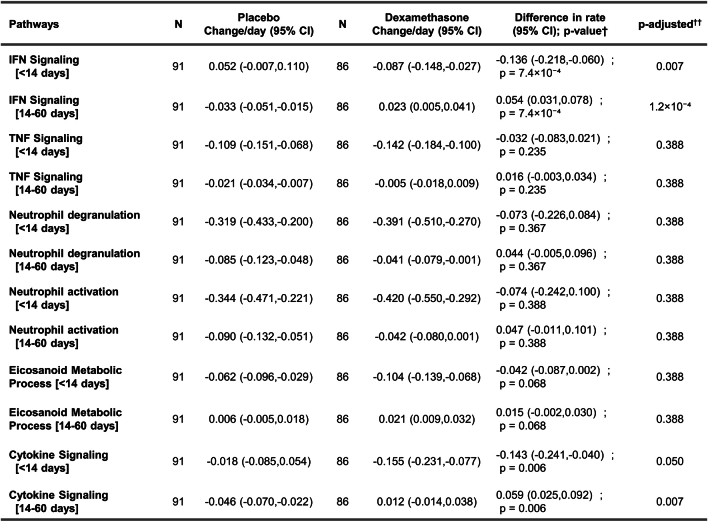
Changes in transcriptional activity of pathophysiological pathways in whole blood in CC- and CT-genotype participants by treatment arm during the first 60 days from randomization

95% CI: Bayesian 95% credible intervals.

†p-value: Derived from crude “Wald-type” tests, calculated as the posterior mean estimate divided by its standard deviation from the MCMC sampling of coefficients in the joint model. All tests were two-sided.

^††^p-values adjusted for multiple comparisons using the Hochberg procedure. CI=confidence interval. IFN=interferon. TNF=tumour necrosis factor.

**Extended Data Table 8:**
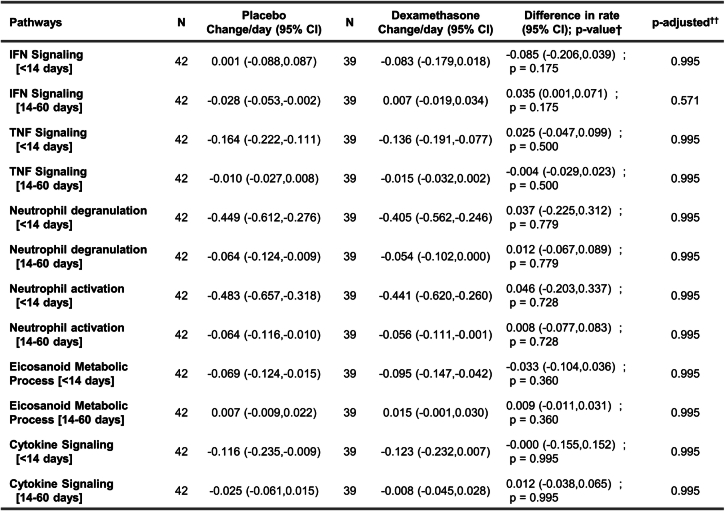
Changes in transcriptional activity of pathophysiological pathways in whole blood in CC-genotype participants by treatment arm during the first 60 days from randomization

95% CI: Bayesian 95% credible intervals.

†p-value: Derived from crude “Wald-type” tests, calculated as the posterior mean estimate divided by its standard deviation from the MCMC sampling of coefficients in the joint model. All tests were two-sided.

^††^p-values adjusted for multiple comparisons using the Hochberg procedure. CI=confidence interval. IFN=interferon. TNF=tumour necrosis factor.

**Extended Data Table 9:**
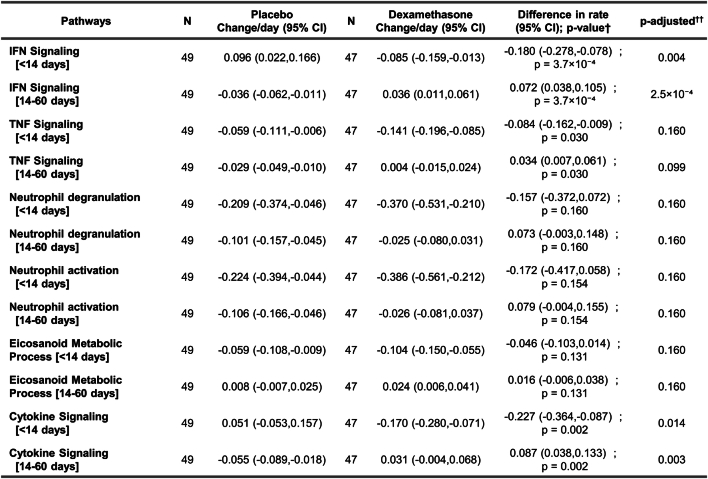
Changes in transcriptional activity of pathophysiological pathways in whole blood in CT-genotype participants by treatment arm during the first 60 days from randomization

95% CI: Bayesian 95% credible intervals.

†p-value: Derived from crude “Wald-type” tests, calculated as the posterior mean estimate divided by its standard deviation from the MCMC sampling of coefficients in the joint model. All tests were two-sided.

^††^p-values adjusted for multiple comparisons using the Hochberg procedure. CI=confidence interval. IFN=interferon. TNF=tumour necrosis factor.

**Extended Data Table 10:**
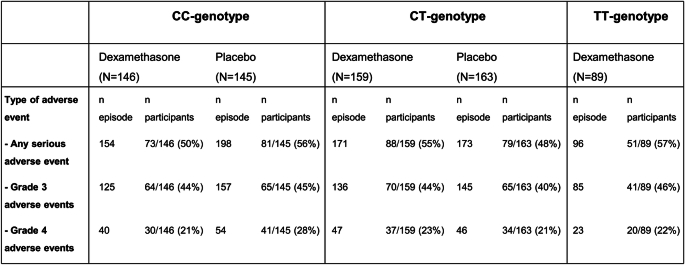
Serious adverse events, and grade 3&4 adverse events, by *LTA4H* genotype

*LTA4H=leukotriene A4 hydrolase*.

## Data Availability

Deidentified trial participant data (including data dictionaries) will be shared on request to the Oxford University Clinical Research Unit (via emailing the senior author, G.E.T., or corresponding author, J.D.). A response will be provided within 4 weeks of receiving the request. The data shared will allow replication of the primary analysis contained within the current manuscript. The study protocol^27^ and statistical analysis plan^28^ have been published and are available under open access. Proteomic and transcriptomic data used for this analysis have been deposited into a public repository and are available via Dryad at 10.5061/dryad.f1vhhmh7v (ref. ^39^).

## Code availability

R analysis code has been made publicly available via GitHub at oucru-biostats/LAST-ACT: Analysis code for LAST ACT trial, https://github.com/oucru-biostats/LAST-ACT.
