# Supplementary material for: Genotype-stratified adjunctive dexamethasone for tuberculous meningitis in HIV-negative adults: a randomized controlled phase 3 trial
Source: Nat Med. 2026 Jan 15;32(3):849–58. doi: 10.1038/s41591-025-04138-z (PMC13004695; doi:10.1038/s41591-025-04138-z)
Supplement: Supplementary file 1 — Supplementary Text 1 and 2 (this includes a list of LAST-ACT investigators), Figs. 1–26 and Tables 1–56. [file 41591_2025_4138_MOESM1_ESM.pdf]

nature medicine

Supplementary information https://doi.org/10.1038/s41591-025-04138-z

Genotype-stratified adjunctive dexamethasone for tuberculous meningitis in HIV-negative adults: a randomized controlled phase 3 trial

In the format provided by the authors and unedited

Supplementary appendix

Table of contents

Page 7. List of investigators

Page 8. Text S1. Committee approvals for the LAST ACT trial

Page 9. Text S2. Trial Steering Committee and Data Monitoring Committee

Page 10. Figure S1. Comparison of CSF immunopathogenesis pathways between all genotypes at baseline

Page 11. Figure S2. Comparison of whole blood transcriptional profiles in key pathways between all genotypes at baseline

Page 12. Figure S3. Death in prespecified sub-groups in the ITT population

Page 13. Figure S4. Death or new neurological events by subgroup in the CC/CT-genotype ITT population over 12 months from randomization

Page 14. Figure S5. Death or new neurological events in the per-protocol population CC/CT-genotype participants

Page 15. Figure S6. Exploratory analysis of effects of dexamethasone on primary endpoint and survival, by TBM grade

Page 16. Figure S7. Death over the first 12 months after randomization in the ITT population.

Page 17. Figure S8. Survival in pre-specified subgroups in the ITT population

Page 18. Figure S9. New neurological events in prespecified sub-groups in the ITT population

Page 19. Figure S10. Open-label use of corticosteroids in prespecified sub-groups in the ITT population, with the primary endpoint as a competing risk

Page 20. Figure S11. Comparison between the two treatment arms for each of the three time-to-event secondary outcomes, in CC- and CT-genotype combined and CC-genotype alone in the ITT population

Page 21. Figure S12. Survival in the CC/CT-genotype per-protocol population

Page 22. Figure S13. Death in prespecified sub-groups in the per-protocol population

Page 23. Figure S14. New neurological events in the per-protocol population

Page 24. Figure S15. Open label use of corticosteroids in prespecified sub-groups in the per

protocol population, with the primary endpoint as a competing risk

Page 25. Figure S16. Comparison of secondary outcomes between the two treatment arms, stratified by genotype, in the per-protocol population

Page 26. Figure S17. Comparison of change in CSF pathways activity from day 0 to 30 in placebo or dexamethasone treated CC/CT-genotype participants CSF pathways

Page 27. Figure S18. Comparison of change in CSF pathways activity from day 0 to 30 in dexamethasone treated CC-, CT- and TT-genotype participants

Page 28. Figure S19. Comparison of change in CSF cytokine concentrations from day 0 to 30 in placebo or dexamethasone treated CC-genotype (a) and CT-genotype (b) participants

Page 29. Figure S20. Comparison of change in CSF pathways activity from day 0 to 30 in placebo or dexamethasone treated CC-genotype (a) or CT-genotype (b) participants

Page 30. Figure S21. Comparison of change in whole blood transcriptional profiles from day 0 to 60 in placebo or dexamethasone treated CC/CT-genotype participants

Page 31. Figure S22. Comparison of change in whole blood transcriptional profiles from day 0 to 60 in placebo or dexamethasone treated CC- genotype (a) and CT-genotype (b) participants

Page 32. Figure S23. Exploratory comparison of CSF cytokines between CC and CT genotypes at day 30

Page 33. Figure S24. Exploratory comparison of CSF immunopathogenesis pathways between CC and CT genotypes at day 30

Page 34. Figure S25. Individual participant meta-analysis of 9-month survival in dexamethasone and placebo treated participants in the 2004 and current trial

Page 36. Figure S26. CONSORT 2025 statement checklist

Page 41. Table S1. Reasons and numbers excluded for the per-protocol analysis

Page 42. Table S2. Supplementary baseline data in the randomized (CC/CT-genotype) and TT-genotype participants (additional to table 1 in the main text)

Page 45. Table S3. Supplementary baseline data by genotype

Page 48. Table S4. The Representativeness of Study Participants

Page 50. Table S5. Baseline characteristics of participants with and without CSF proteomic profiling

Page 53. Table S6. Baseline characteristics of participants with and without transcriptomics profiling

Page 57. Table S7. Primary outcome non-inferiority and superiority analysis for ITT randomized participants

Page 58. Table S8. Restricted mean time lost (RMTL) for all-cause mortality or new neurological event, corrected for genotype and MRC grade in the ITT population.

Page 59. Table S9. Restricted mean time lost in prespecified sub-groups in the ITT population

Page 61. Table S10. Primary endpoint non-inferiority and superiority analysis in the per-protocol population

Page 62. Table S11. Primary endpoint and prespecified sub-group analysis in the per-protocol population

Page 64. Table S12. Restricted mean time lost (RMTL) for all-cause mortality or new neurological event, corrected for genotype and MRC grade, in the per-protocol population

Page 65. Table S13. Absolute event risk by genotype and treatment arm

Page 66. Table S14. Overall incidence of death over the first 12 months after randomization in the ITT population

Page 67. Table S15. Death overall and in prespecified subgroups in the ITT population.

Page 68. Table S16. Death restricted mean time lost (RMTL) in the ITT population

Page 69. Table S17. Overall risk of first neurological event over the first 12 months after randomization in the ITT population

Page 70. Table S18. New neurological events in prespecified sub-groups in the ITT population.

Page 71. Table S19. Restricted mean time lost (RMTL) to new neurological events in the ITT population, with death as a competing risk

Page 72. Table S20. Overall risk of first use of open-label corticosteroid over the first 12 months

after randomization in the ITT population

Page 73. Table S21. First use of open label corticosteroids in the ITT population

Page 74. Table S22. Restricted mean time lost (RMTL) to new use of open label corticosteroids in prespecified sub-groups in the ITT population, with death as competing risk

Page 75. Table S23. Overall risk of first use of open-label corticosteroids over the first 12 months after randomization in the ITT population

Page 76. Table S24. Prespecified sub-group analysis of first use of open label corticosteroids in the ITT population

Page 77. Table S25. Restricted mean time lost (RMTL) to first use of open label corticosteroids in prespecified subgroups in the ITT population

Page 78. Table S26. Total numbers given open-label dexamethasone within 12 months, by subgroups in the ITT population

Page 79. Table S27. Summary of reasons to use open-label corticosteroid treatment over the first 12 months after randomization in the ITT population

Page 83. Table S28. Neurological disability at 12 months from randomization, by treatment arms in the ITT population

Page 84. Table S29. All modified Rankin scores at 12 months from randomization in the ITT population

Page 85. Table S30. Overall incidence of death over the first 12 months after randomization in the per-protocol population

Page 86. Table S31. Deaths in prespecified subgroups in the per-protocol population

Page 87. Table S32. Restricted mean time lost (RMTL) for deaths in the per-protocol population

Page 88. Table S33. Overall risk of first neurological event over the first 12 months after randomization in the per-protocol population

Page 89. Table S34. New neurological events in prespecified sub-groups in the per-protocol population

Page 90. Table S35. Restricted mean time lost (RMTL) for new neurological events in the per-

protocol population

Page 91. Table S36. Overall risk of first use of open-label corticosteroid over the first 12 months after randomization in the per-protocol population

Page 92. Table S37. First use of open label corticosteroids in prespecified sub-groups in the per protocol population

Page 93. Table S38. New use of open label corticosteroids in prespecified sub-groups, with death as competing risk in the per-protocol population

Page 94. Table S39. Overall risk of first use of open-label corticosteroid over the first 12 months after randomization in the per-protocol population

Page 95. Table S40. New use of open label corticosteroids in prespecified subgroups in the per-protocol population

Page 96. Table S41. Restricted mean time lost (RMTL) for new use of open label corticosteroids with the primary endpoint as a competing risk in the per-protocol population

Page 97. Table S42. Death or disability in the per-protocol population

Page 98. Table S43. All modified Rankin scores at 12 months from randomization in the per-protocol population

Page 99. Table S44. Serious adverse events by treatment arm in the CC-genotype

Page 103. Table S45. Serious adverse events by treatment arm in the CT-genotype

Page 107. Table S46. Serious adverse events and Grade 3 or 4 adverse events in the TT-genotype (N=89; all received dexamethasone)

Page 109. Table S47: Summary of serious adverse events, shown by reasons for which they were considered serious, not shown by study arm

Page 120. Table S48. Summary of serious adverse events possibly, probably, or definitely related to the study drug in CC genotype participants

Page 121. Table S49. Summary of serious adverse events possibly, probably, or definitely related to the study drug in CT-genotype participants

Page 122. Table S50. Summary of serious adverse events possibly, probably, or definitely related to the study drug in TT-genotype participants (N=89; all treated with

dexamethasone)

Page 123. Table S51. Summary of Grade 3&4 laboratory abnormalities in CC- and CT- and TT-genotype participants

Page 125. Table S52. Exploratory analysis of associations of day 30 CSF cytokines (a, b) and CSF inflammatory pathway expression (c, d), with the primary outcome, with and without adjustment for LTA4H genotype, dexamethasone/placebo allocation, and baseline cytokines

Page 127. Table S53. Baseline characteristics of the two trials included in the individual participant data meta-analysis

Page 128. Table S54. Survival over the first 9 months after randomization, by study and arm

Page 129. Table S55. Diagnostic criteria for tuberculous meningitis

Page 131. Table S56. The Modified Rankin Scale

Page 132. References

List of investigators

The following investigators participated in the conduct of the LAST ACT trial and are all co-authors of the trial report. Investigators are listed by group, and then alphabetically.

Pham Ngoc Thach Hospital for Tuberculosis and Lung Disease, Ho Chi Minh City, Vietnam

Truong Thi Anh, Nguyen Duc Bang, Luu Tuan Bang, Nguyen Le Bao, Pham Hung Cuong, Phan Thuong Dat, Pham Anh Dung, Nguyen Duc Dung, Dang Minh Duong, Do Chau Giang, Dang Thi Minh Ha, Pham Thu Hang, Vu Dang Khoa, Nguyen Huu Lan, Huynh Thi Mai Ly, Nguyen Huu Minh, Huynh Phat, Tran Nguyen Hoang Phuong, Tran Thi Phuong, Nguyen Van Song, Nguyen Thi Mai Trang, Nguyen Nang Vien, Nguyen Nhu Viet

Hospital for Tropical Diseases, Ho Chi Minh City, Vietnam

Le Thi My Chau, Nguyen Van Vinh Chau, Lai Ngoc Diem, Nguyen Thi Ha, Bui Thi Bich Hanh, Nguyen Ho Hong Hanh, Tran Minh Luan, Nguyen Thi Kieu My, Ho Dang Trung Nghia, Tran Bao Nhu, Pham Kieu Nguyet Oanh, Nguyen Hoan Phu, Nguyen Luong Nha Phuong, Van Thi Xuan Quynh, Nguyen Truc Thanh, Vu Thi Kim Thoa

Oxford University Clinical Research Unit, Ho Chi Minh City, Vietnam

Guy E. Thwaites. Biostatistics group: Ronald B. Geskus, Lam Phung Khanh, Le Thi Phuong Thao, Dong Huu Khanh Trinh, Marcel Wolbers. Clinical trials unit: Pham Thi Anh Dao, Nguyen Thi Phuong Dung, Nguyen Thuy Hang, Phan Thi Ngoc Hue, Dau Thi Thanh Huong, Evelyne Kestelyn, Le Quoc Khanh, Vu Phuong Mai, Lam Hong Bao Ngoc, Tran Thi Bich Ngoc, Nguyen Thi Thao Nguyen, Dong Quynh Nhu, Trinh Khanh Phuong, Nguyen Than Ha Quyen, Le Thi Thao, Vu Thai Kim Thi, Dang Trong Thuan, Vo Thi Thanh Thuy, Vu Thi Thuy, Nguyen Bao Tran, Nguyen Thi Huyen Trang, Ninh Thi Thanh Van, Celine Vidaillac. Tuberculosis group: Tran Dinh Dinh, Joseph Donovan, Vu Thi Mong Dung, Vu Thi Ngoc Ha, Hoang Thanh Hai, Nguyen Thi Thu Hiep, Pham Thi Kim, Phung Vo Khac Nguyen, Le Thanh Hoang Nhat, Nguyen Le Quang, Dang Van Thanh, Dang Anh Thu, Nguyen Thi Anh Thu, Nguyen Thuy Thuong Thuong, Trinh Thi Bich Tram, Nghiem Huyen Trang

Supplementary text

Text S1. Committee approvals for the LAST ACT trial

Ethical approvals for the LAST ACT trial (with approval numbers) were as follows:

1. The Oxford Tropical Research Ethics Committee: 52-16

2. The ethical committee of the Hospital for Tropical Diseases: 37/HDDD

3. The ethical committee of Pham Ngoc Thach Hospital for Tuberculosis and Lung Disease: 1034/HDDD-PNT

4. The Vietnam Ministry of Health: 151/CN-BDGDD

Text S2. Trial Steering Committee and Data Monitoring Committee

Trial Steering Committee

- Chair: Professor Robert Wilkinson (Honorary Professor and Director Wellcome Centre for Infectious Diseases Research in Africa, University of Cape Town, South Africa).
- Professor Nicholas Paton (Infectious Diseases Physician and Clinical Trialist, National University of Singapore, Singapore).
- Professor Ben Marais (Senior Tuberculosis Researcher and Trialist, University of Sydney, Australia).
- Dr Truong Huu Khanh (Infectious Diseases Physician, Paediatric Hospital Number 1, Ho Chi Minh City, Vietnam).

Data Monitoring Committee

- Chair: Professor Sarah Walker (Senior Statistician and Clinical Trialist, MRC Clinical Trials Unit, University College London).
- Professor Graeme Meintjes (Senior Infectious Diseases/HIV Physician, University of Cape Town, South Africa).
- Professor Rovina Ruslami (Senior Clinical Pharmacologist and TBM Researcher, Universitas Padjadjaran, Bandung, Indonesia).

Supplementary figures

Figure S1. Comparison of CSF immunopathogenesis pathways between all genotypes at baseline

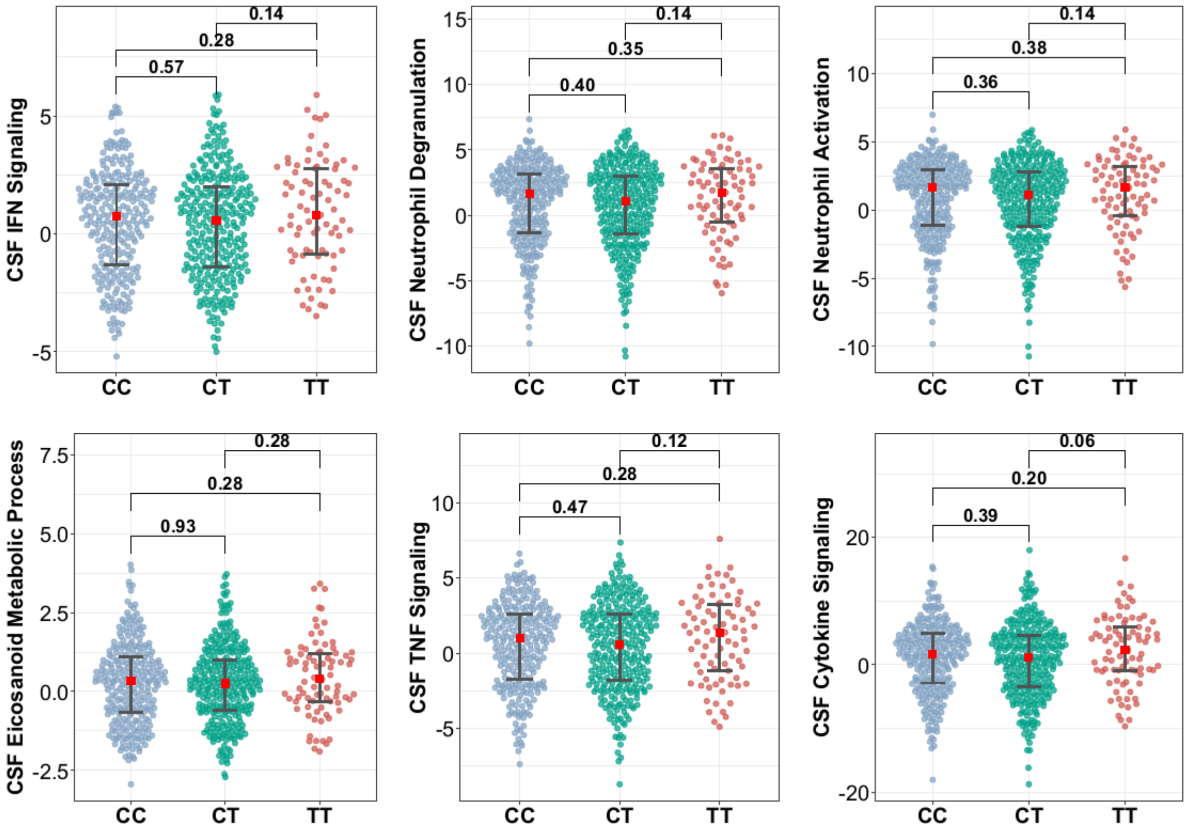

This figure displays the distribution of pathway activity for six immune pathways implicated in TB/TBM pathogenesis across different genotypic groups (CC, CT, TT). Pathway activity in CSF was calculated using a z-score method.[1] The violin plots illustrate the data distribution for each genotype, with individual data points shown as dots. Each red central point indicates the median pathway activity level within each group, and the black horizontal bars indicate the interquartile range. Pairwise comparisons between genotype groups were performed using the Wilcoxon rank-sum test, with the corresponding p-values displayed. CSF=cerebrospinal fluid. IFN=interferon. TBM=tuberculous meningitis. TNF=tumour necrosis factor.

Figure S2. Comparison of whole blood transcriptional profiles in key pathways between all genotypes at baseline

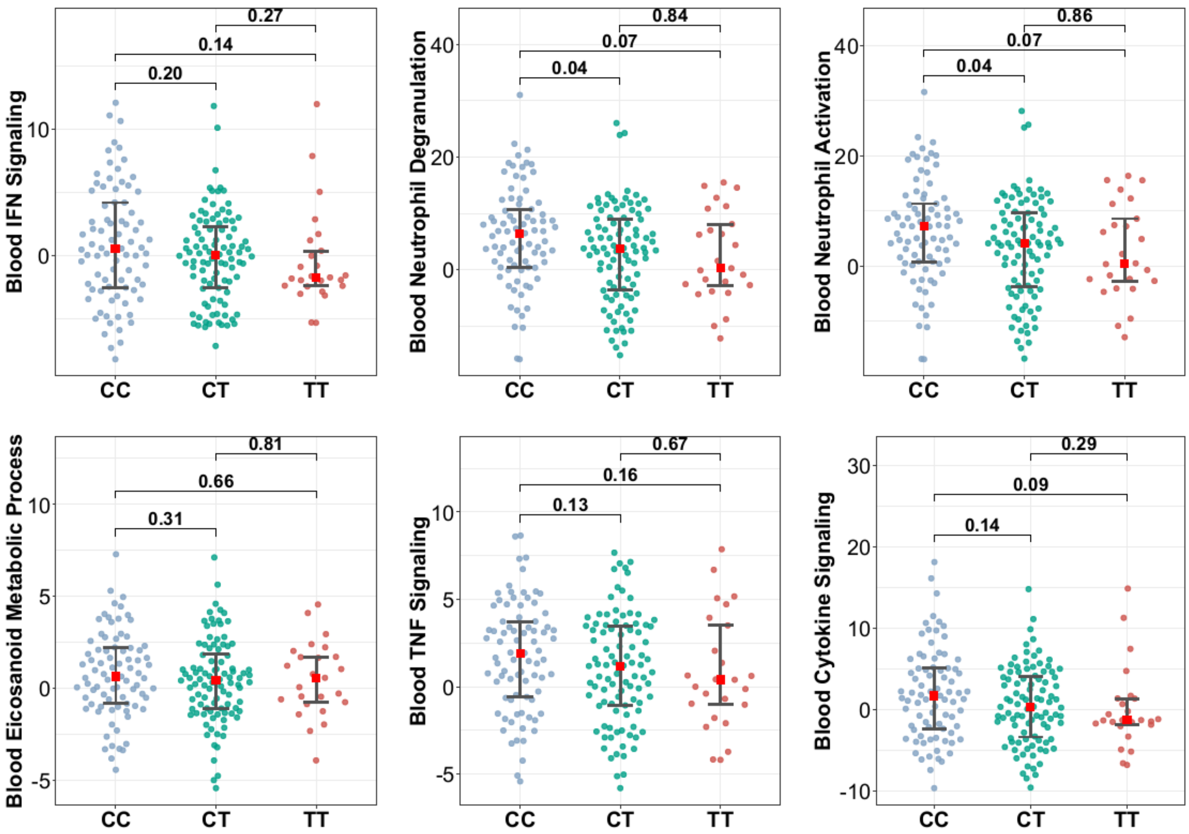

This figure displays the distribution of pathway activity for six immune pathways implicated in TB/TBM pathogenesis across different genotypic groups (CC, CT, TT). Pathway activity in whole blood was calculated using a z-score method.[1] The violin plots illustrate the data distribution for each genotype, with individual data points shown as dots. Each red central point indicates the median pathway activity level within each group, and the black horizontal bars indicate the interquartile range. Pairwise comparisons between genotype groups were performed using the Wilcoxon rank-sum test, with the corresponding p-values displayed. IFN=interferon. TBM=tuberculous meningitis. TNF=tumour necrosis factor.

Figure S3. Death in prespecified sub-groups in the ITT population

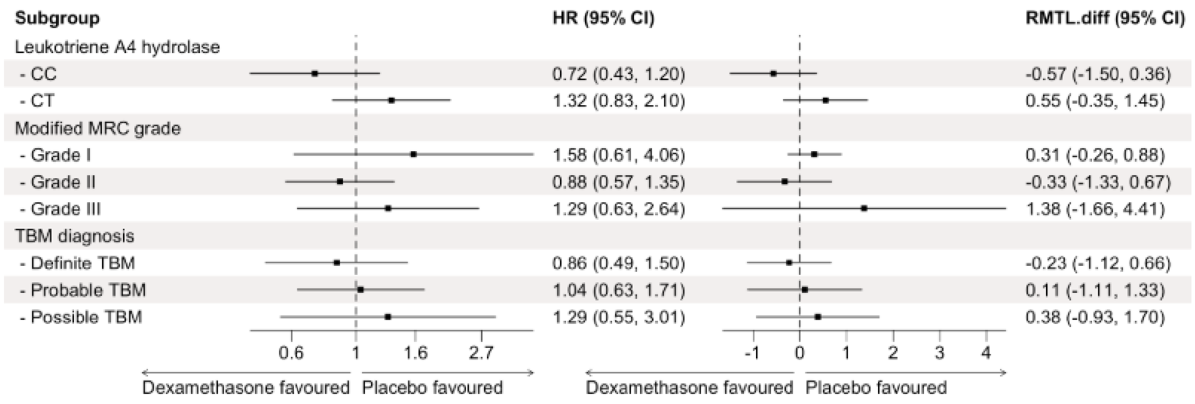

Points represent the estimated hazard ratios and RMTL regarding mortality between two treatment arms in each subgroup. Black lines represent the 95% CIs. No correction for multiplicity is made. CI=confidence interval. HR=hazard ratio. ITT=intention-to-treat. MRC=Medical Research Council. RMTL=restricted mean time lost. TBM=tuberculous meningitis.

Figure S4. Death or new neurological events by subgroup in the CC/CT-genotype ITT population over 12 months from randomization

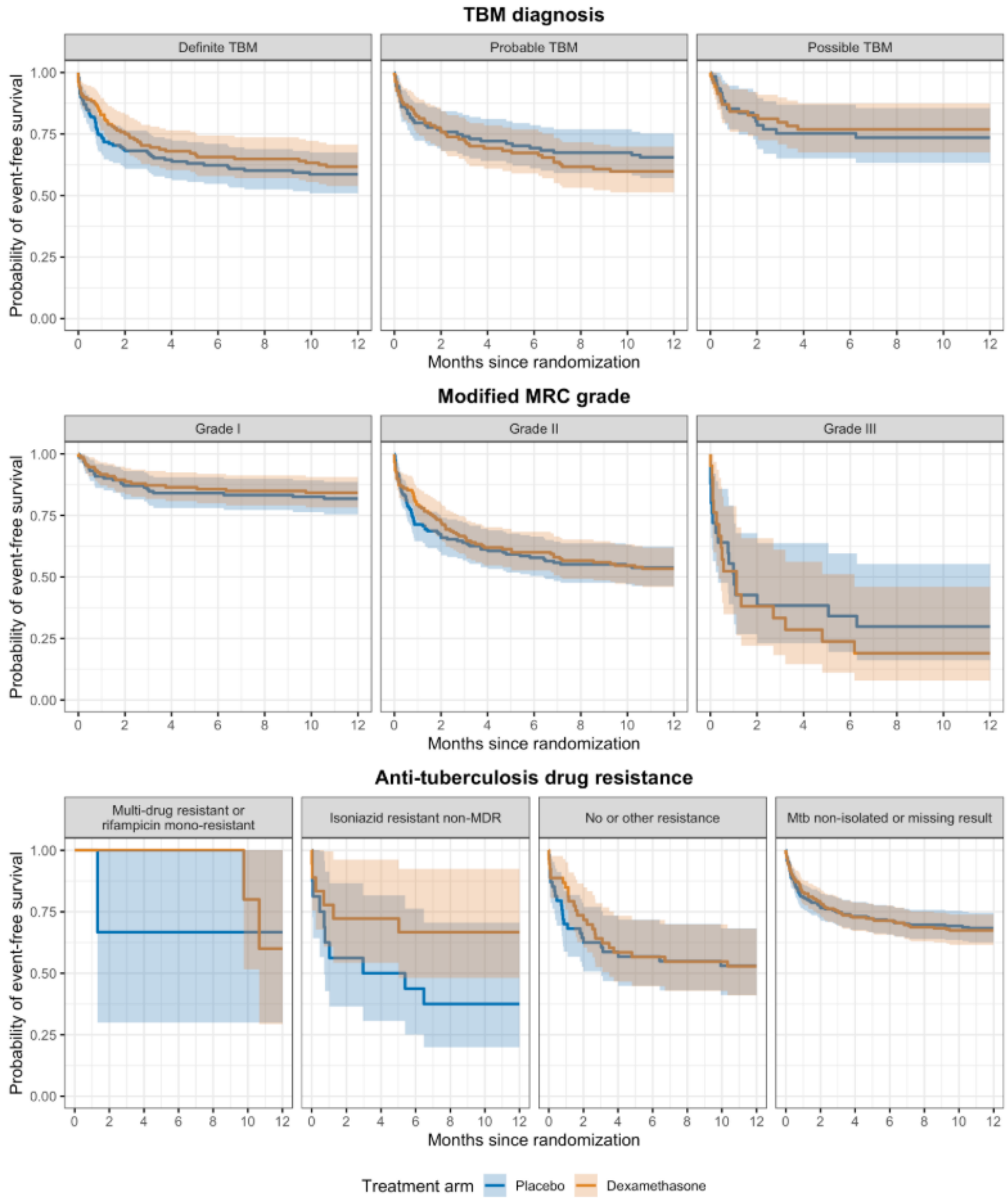

Coloured shading represents 95% confidence intervals. ITT=intention-to-treat. MRC=Medical Research Council. TBM=tuberculous meningitis.

Figure S5. Death or new neurological events in the per-protocol population CC/CT-genotype participants

a)

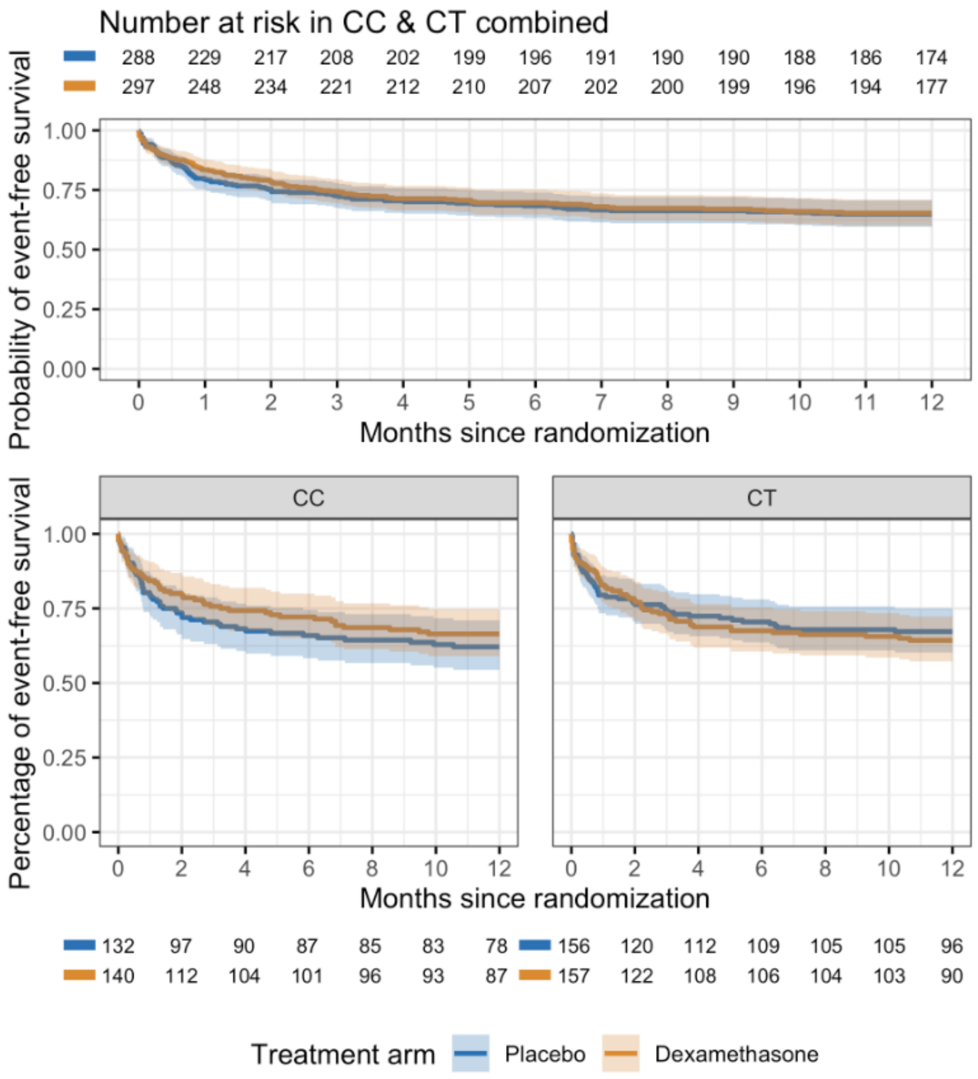

b)

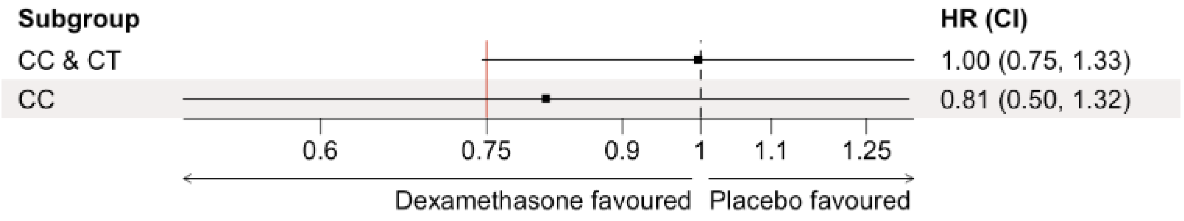

Panel (a) shows Kaplan-Meier curves for the primary endpoint. Coloured shading represents 95% confidence intervals. Panel (b) shows a forest plot displaying the hazard ratio and confidence intervals, with corresponding confidence levels at 96% for CC & CT combined, and 98.3% for CC. The dashed vertical line demonstrates the non-inferiority threshold at hazard ratio = 0.75. For analysis, a Cox proportional hazard model was used, with MRC Grade and LTA4H genotype as strata. Analyses are performed for the following populations; CC&CT-genotype combined: 585 participants, CT-genotype: 313 participants. CC-genotype: 272 participants. MRC=Medical Research Council. LTA4H=leukotriene A4 hydrolase.

Figure S6. Exploratory analysis of effects of dexamethasone on primary endpoint and survival by grade

a) Primary endpoint

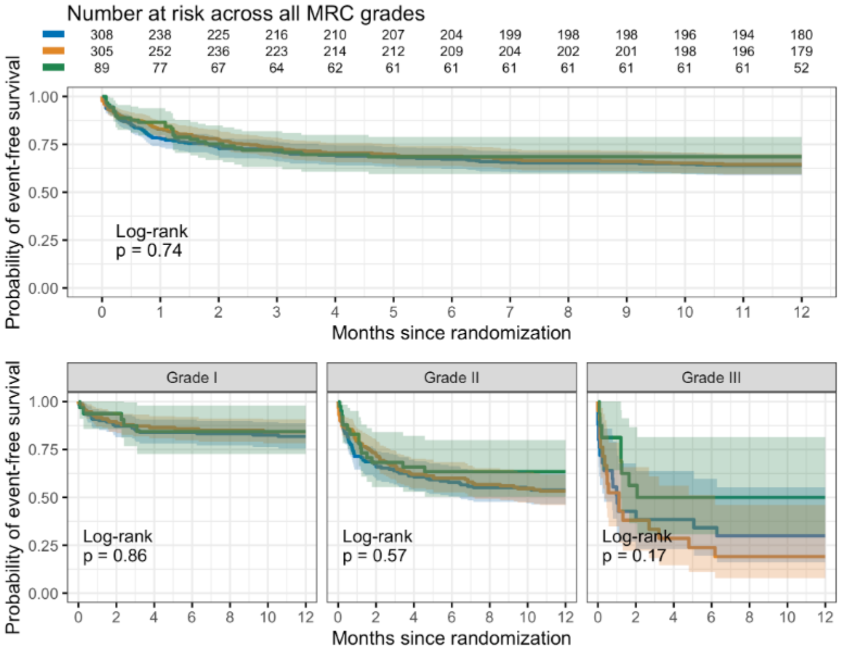

b) Survival

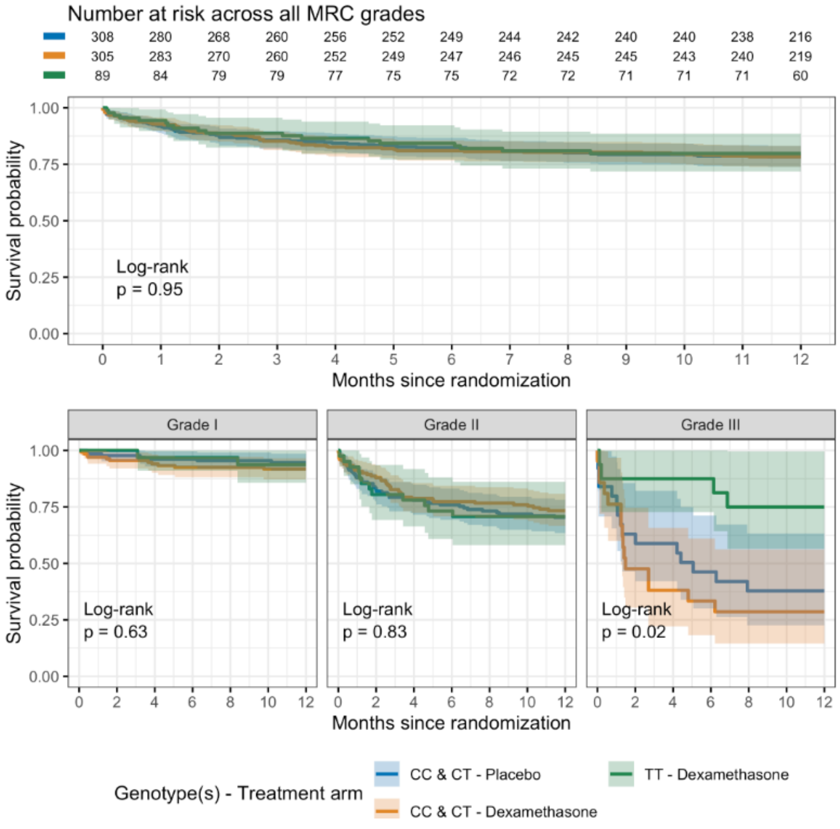

Coloured shading represents 95% confidence intervals. MRC=Medical Research Council.

Figure S7. Death over the first 12 months after randomization in the ITT population

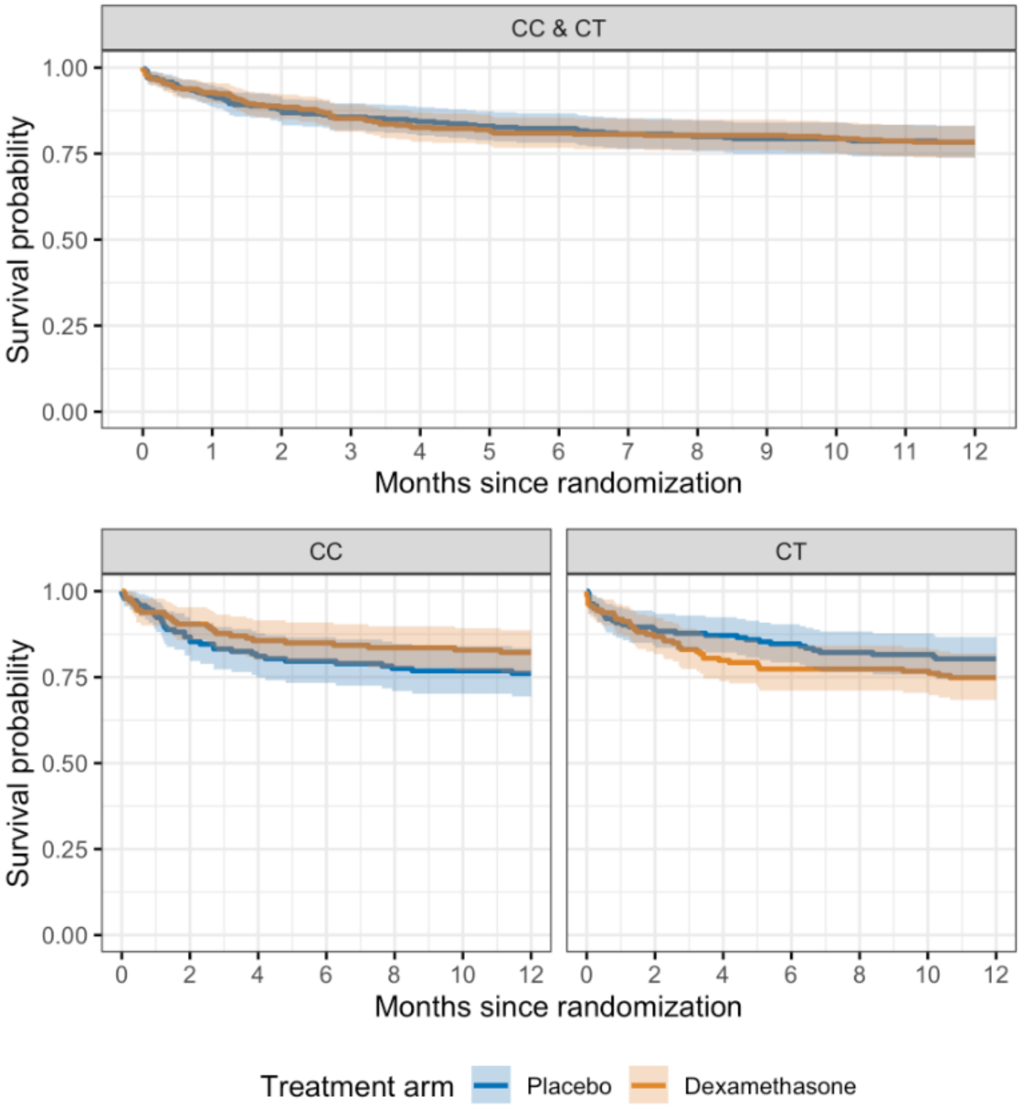

Coloured shading represents 95% confidence intervals. ITT=intention-to-treat.

Figure S8. Survival in pre-specified subgroups in the ITT population

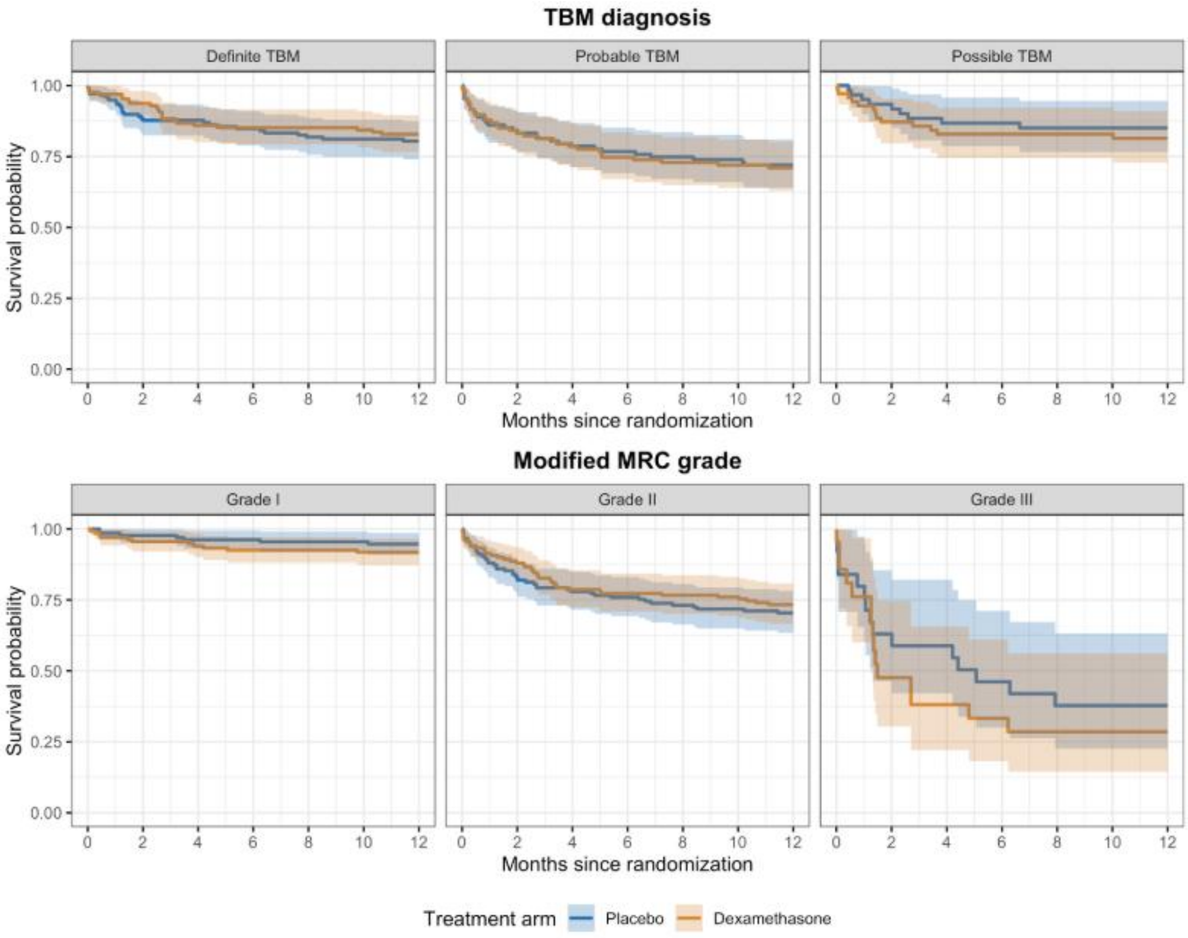

Coloured shading represents 95% confidence intervals. ITT=intention-to-treat. MRC=Medical Research Council. TBM=tuberculous meningitis.

Figure S9. New neurological events in prespecified sub-groups in the ITT population

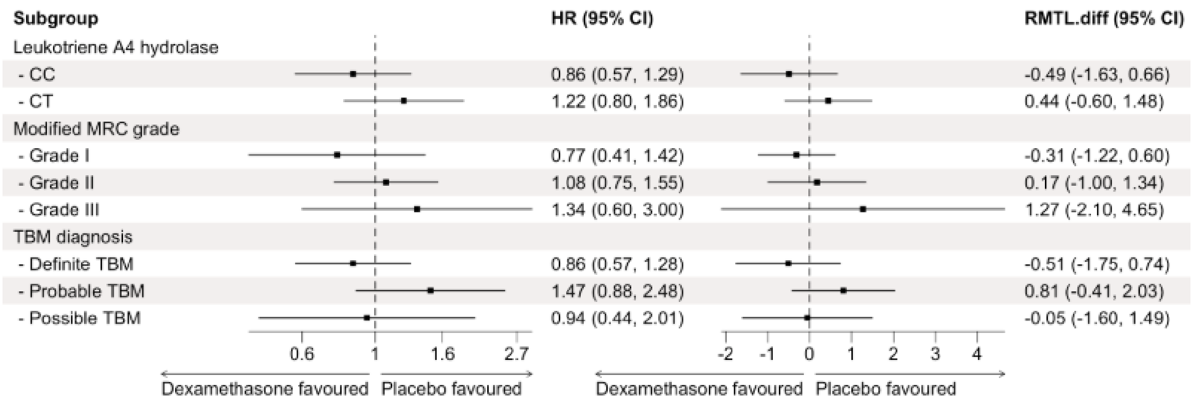

Points represent the estimated hazard ratios and RMTL regarding new neurological events between two treatment arms in each subgroup. Black lines represent the 95% CIs. No correction for multiplicity is made. CI=confidence interval. HR=hazard ratio. ITT=intention-to-treat. MRC=Medical Research Council. RMTL=restricted mean time lost. TBM=tuberculous meningitis.

Figure S10. Open-label use of corticosteroids in prespecified sub-groups in the ITT population, with the primary endpoint as a competing risk

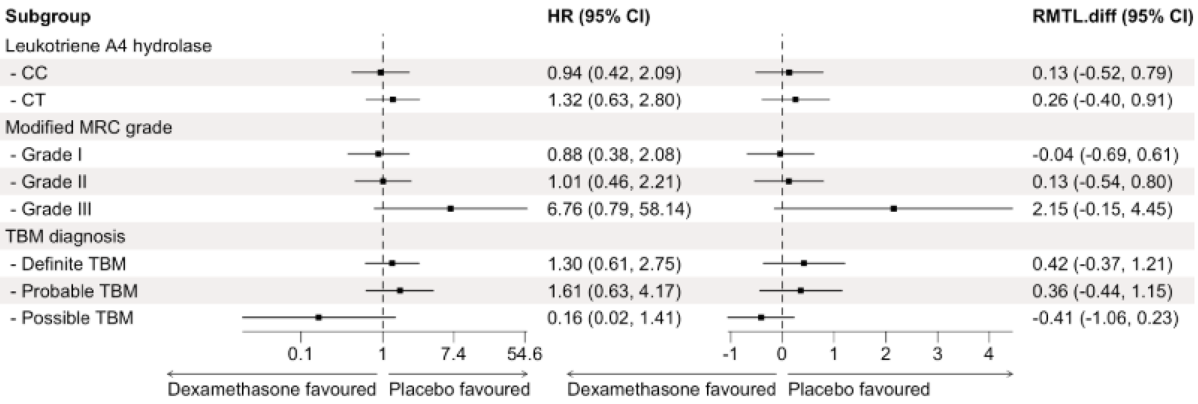

Points represent the estimated hazard ratios and RMTL regarding the use of open label corticosteroids between two treatment arms in each subgroup. Black lines represent the 95% CIs. No correction for multiplicity is made. CI=confidence interval. HR=hazard ratio. ITT=intention-to-treat. MRC=Medical Research Council. RMTL=restricted mean time lost. TBM=tuberculous meningitis.

Figure S11. Comparison between the two treatment arms for each of the three time-to-event secondary outcomes, in CC- and CT-genotype combined and CC-genotype alone in the ITT population

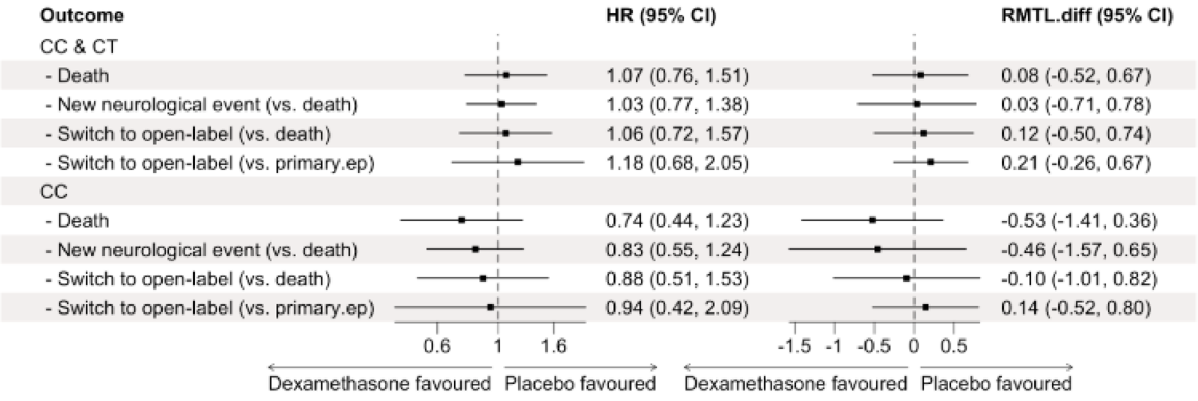

Points represent the estimated hazard ratios and RMTL regarding the three secondary endpoints between two treatment arms. Black lines represent the 95% CIs. No correction for multiplicity is made. CI=confidence interval. HR=hazard ratio. ITT=intention-to-treat. RMTL=restricted mean time lost.

Figure S12. Survival in the CC/CT-genotype per-protocol population

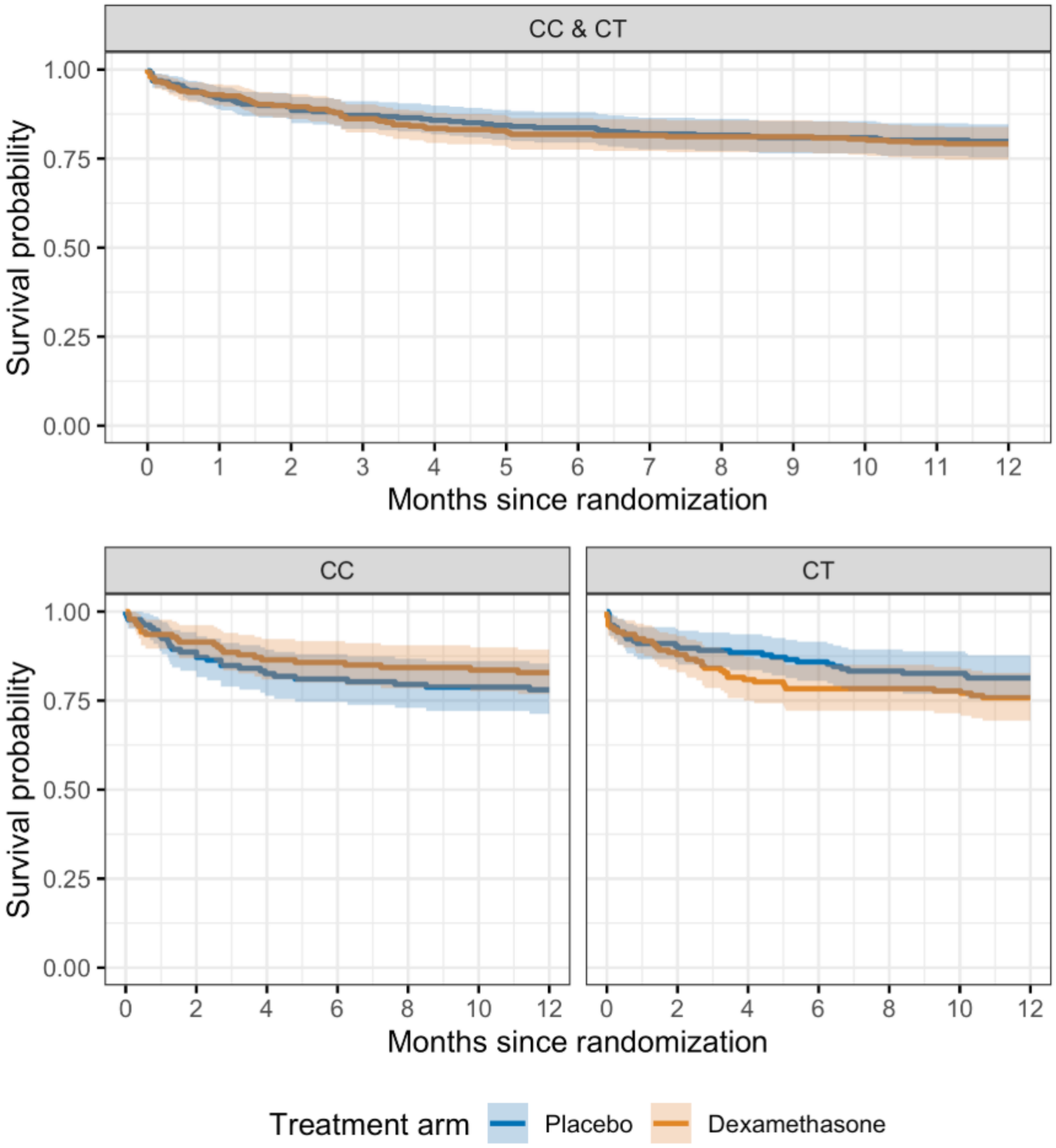

Coloured shading represents 95% confidence intervals.

Figure S13. Death in prespecified sub-groups in the per-protocol population

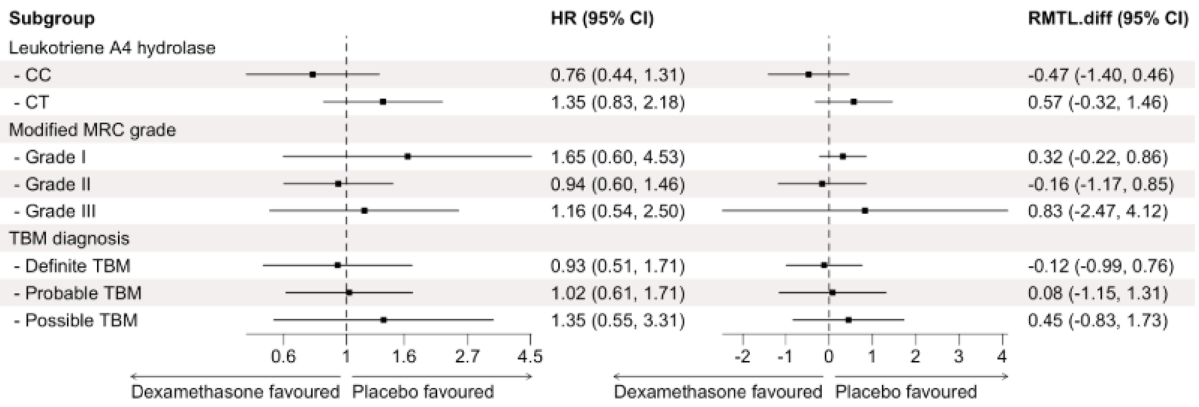

Points represent the estimated hazard ratios and RMTL regarding mortality between two treatment arms in each subgroup. Black lines represent the 95% CIs. No correction for multiplicity is made. CI=confidence interval. HR=hazard ratio. ITT=intention-to-treat. MRC=Medical Research Council. RMTL=restricted mean time lost. TBM=tuberculous meningitis.

Figure S14. New neurological events in the per-protocol population

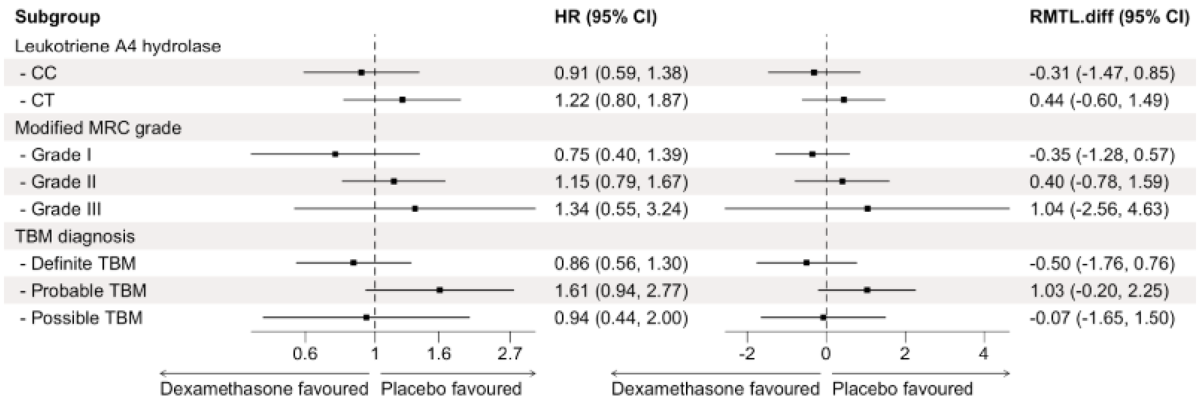

Points represent the estimated hazard ratios and RMTL regarding new neurological events between two treatment arms in each subgroup. Black lines represent the 95% CIs. No correction for multiplicity is made. CI=confidence interval. HR=hazard ratio. ITT=intention-to-treat. MRC=Medical Research Council. RMTL=restricted mean time lost. TBM=tuberculous meningitis.

Figure S15. Open label use of corticosteroids in prespecified sub-groups in the per-protocol population, with the primary endpoint as a competing risk

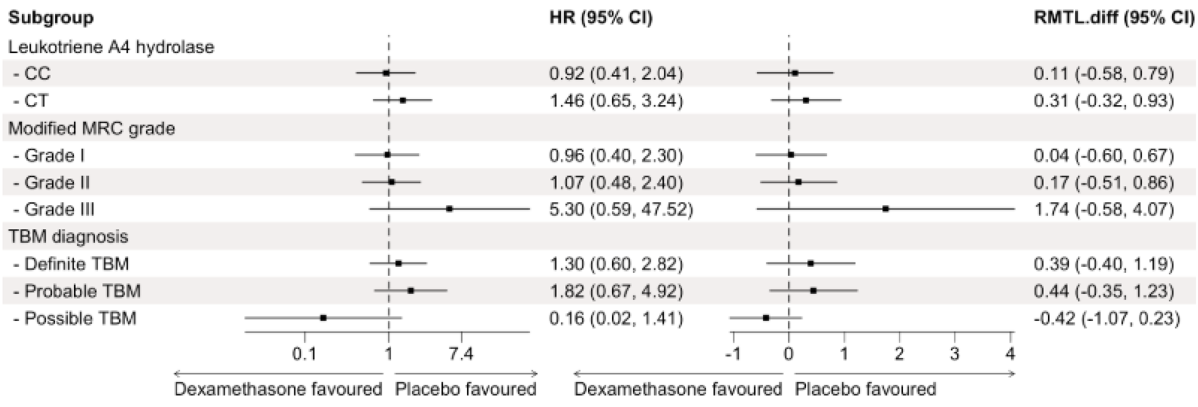

Points represent the estimated hazard ratios and RMTL regarding the use of open label corticosteroids between two treatment arms in each subgroup. Black lines represent the 95% CIs. No correction for multiplicity is made. CI=confidence interval. HR=hazard ratio. ITT=intention-to-treat. MRC=Medical Research Council. RMTL=restricted mean time lost. TBM=tuberculous meningitis.

Figure S16. Comparison of secondary outcomes between the two treatment arms, stratified by genotype, in the per-protocol population

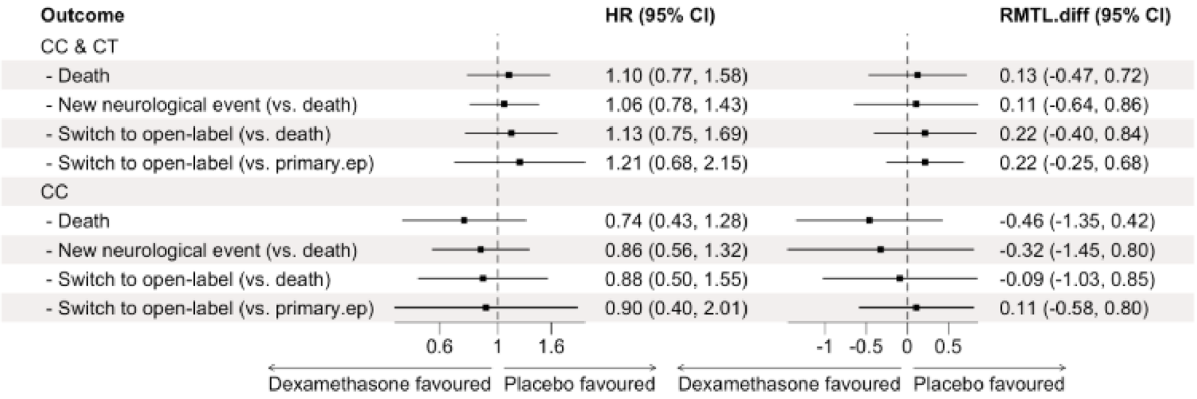

Points represent the estimated hazard ratios and RMTL regarding the three secondary endpoints between two treatment arms. Black lines represent the 95% CIs. No correction for multiplicity is made. CI=confidence interval. HR=hazard ratio. ITT=intention-to-treat. MRC=Medical Research Council. RMTL=restricted mean time lost. TBM=tuberculous meningitis.

Figure S17. Comparison of change in CSF pathways activity from day 0 to 30 in placebo or dexamethasone treated CC/CT-genotype participants CSF pathways

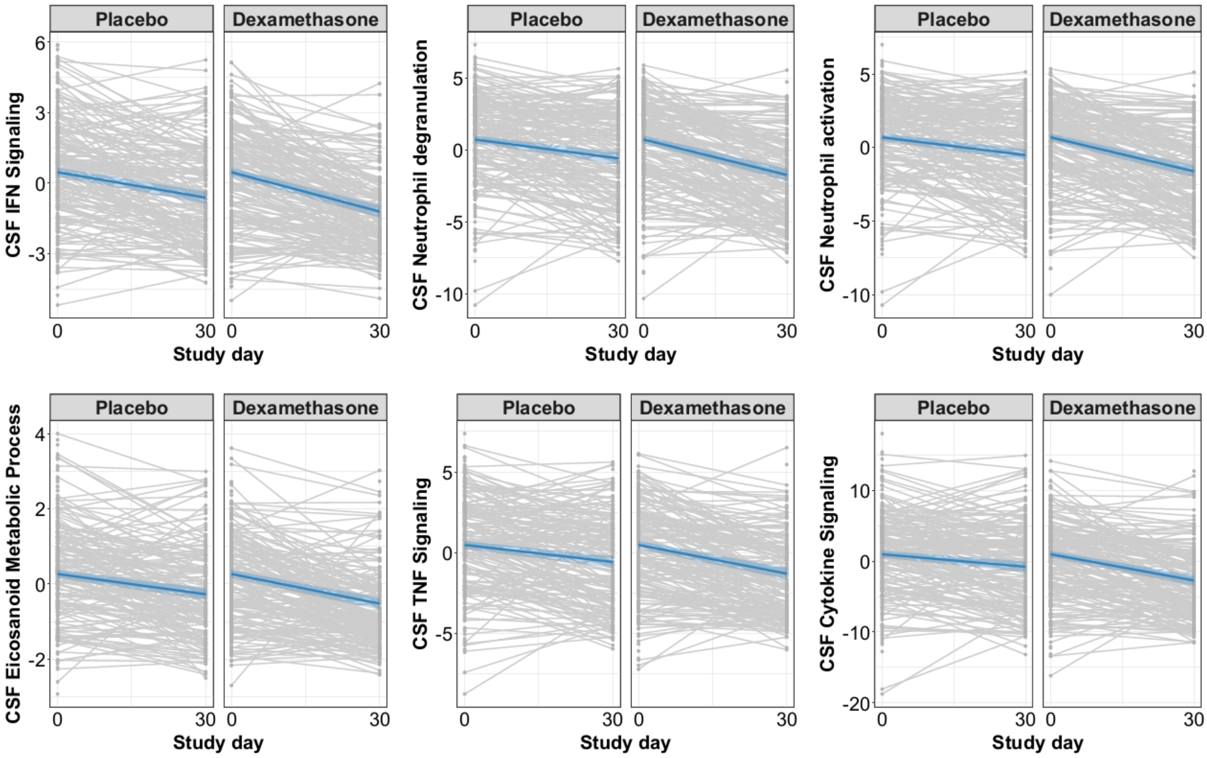

The figure shows changes in six CSF pathway activities from day 0 to day 30 in placebo- or dexamethasone-treated participants with CC/CT- genotype. Pathway activity in CSF was calculated using a z-score method. Each line represents an individual participant. The solid blue lines indicate the fitted mean cytokine levels at each time point, with shaded 95% Bayesian credible intervals obtained via Markov Chain Monte Carlo (MCMC) estimation from the longitudinal sub-model of the Bayesian joint model. CSF=cerebrospinal fluid. IFN=interferon. TNF=tumour necrosis factor.

Figure S18. Comparison of change in CSF pathways activity from day 0 to 30 in dexamethasone treated CC-, CT- and TT-genotype participants

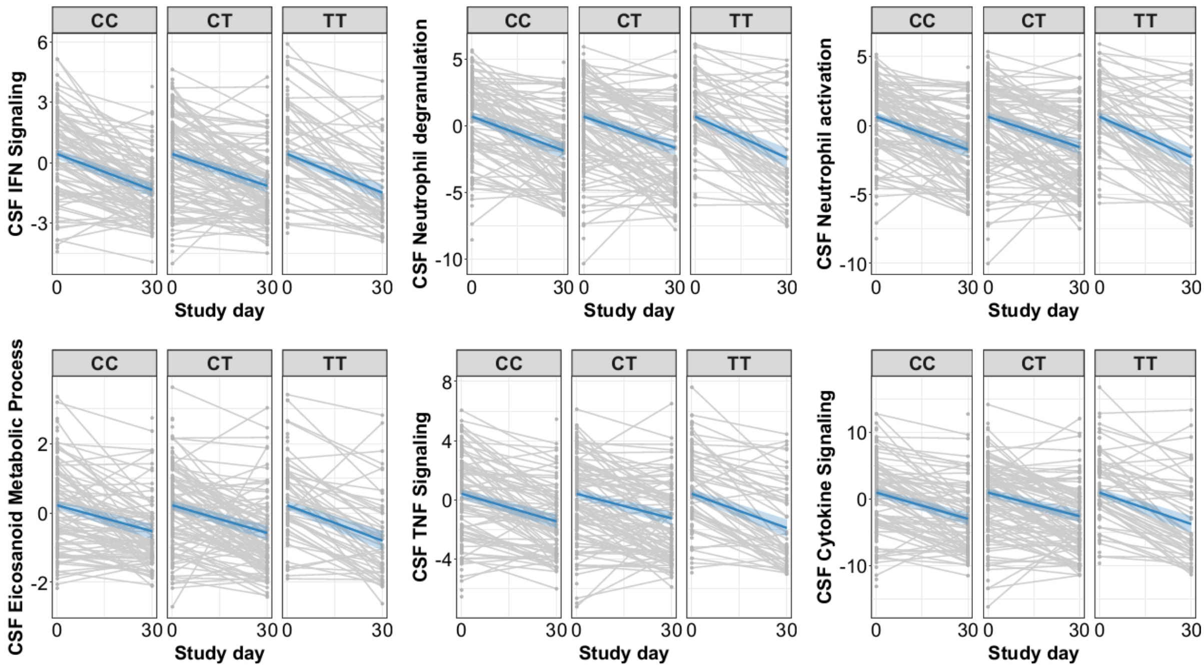

The figure shows changes in six CSF pathway activities from day 0 to day 30 in dexamethasone treated CC-, CT- and TT-genotype participants. Pathway activity in CSF was calculated using a z-score method. Each line represents an individual participant. The solid blue lines indicate the fitted mean cytokine levels at each time point, with shaded 95% Bayesian credible intervals obtained via Markov Chain Monte Carlo (MCMC) estimation from the longitudinal sub-model of the Bayesian joint model. CSF=cerebrospinal fluid. IFN=interferon. TNF=tumour necrosis factor.

Figure S19. Comparison of change in CSF cytokine concentrations from day 0 to 30 in placebo or dexamethasone treated CC-genotype (a) and CT-genotype (b) participants

a)

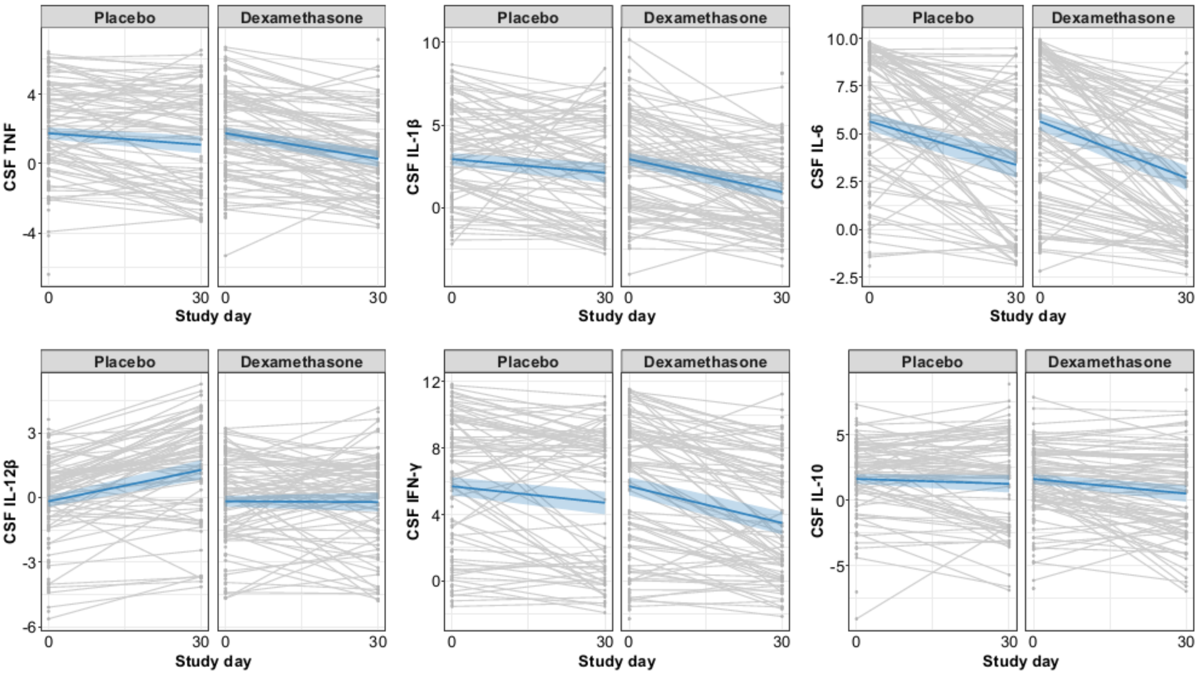

b)

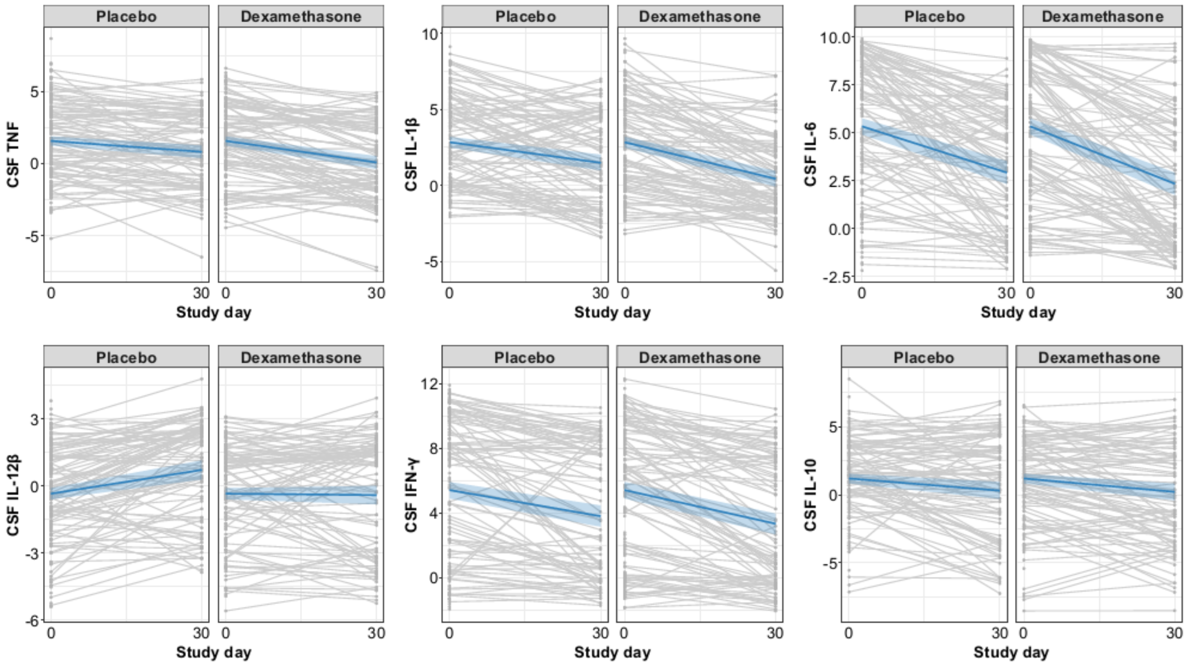

The figure shows changes in six CSF cytokine levels (NPX log2 normalized expression) from day 0 to day 30 in placebo- or dexamethasone-treated participants with the CC-genotype (a) and CT-genotype (b). Each line represents an individual participant. The solid blue lines indicate the fitted mean cytokine levels at each time point, with shaded 95% Bayesian credible intervals obtained via Markov Chain Monte Carlo (MCMC) estimation from the longitudinal sub-model of the Bayesian joint model. CSF=cerebrospinal fluid. IFN=interferon. IL=interleukin. TNF=tumour necrosis factor.

Figure S20. Comparison of change in CSF pathways activity from day 0 to 30 in placebo or dexamethasone treated CC-genotype (a) or CT-genotype (b) participants

a)

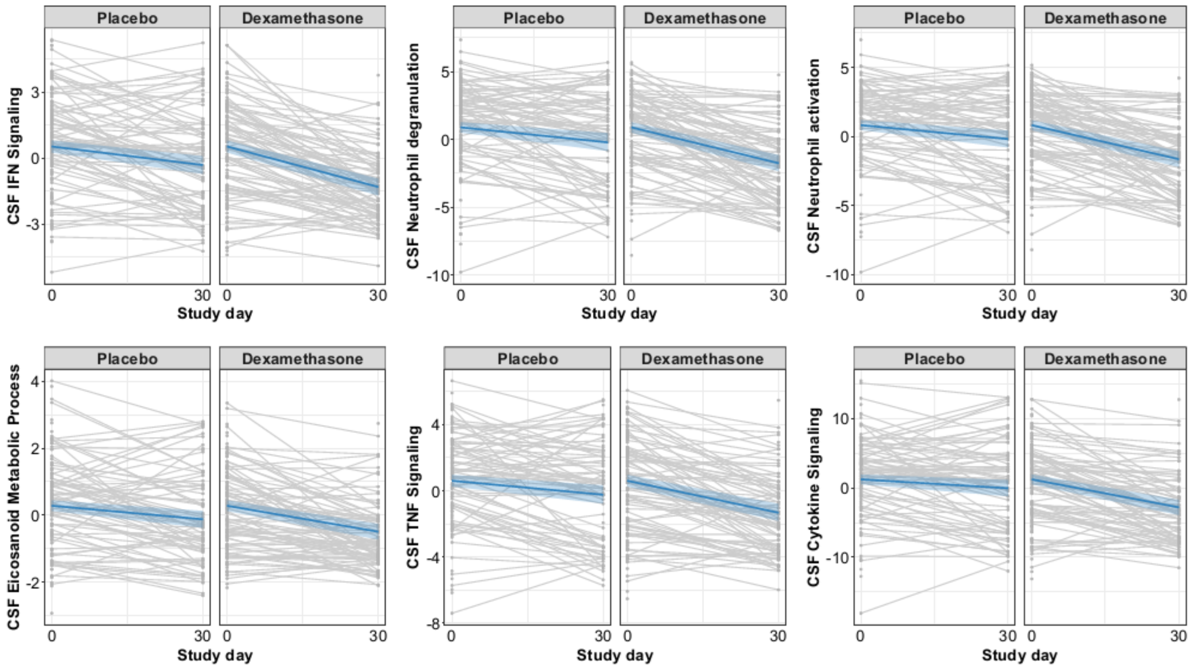

b)

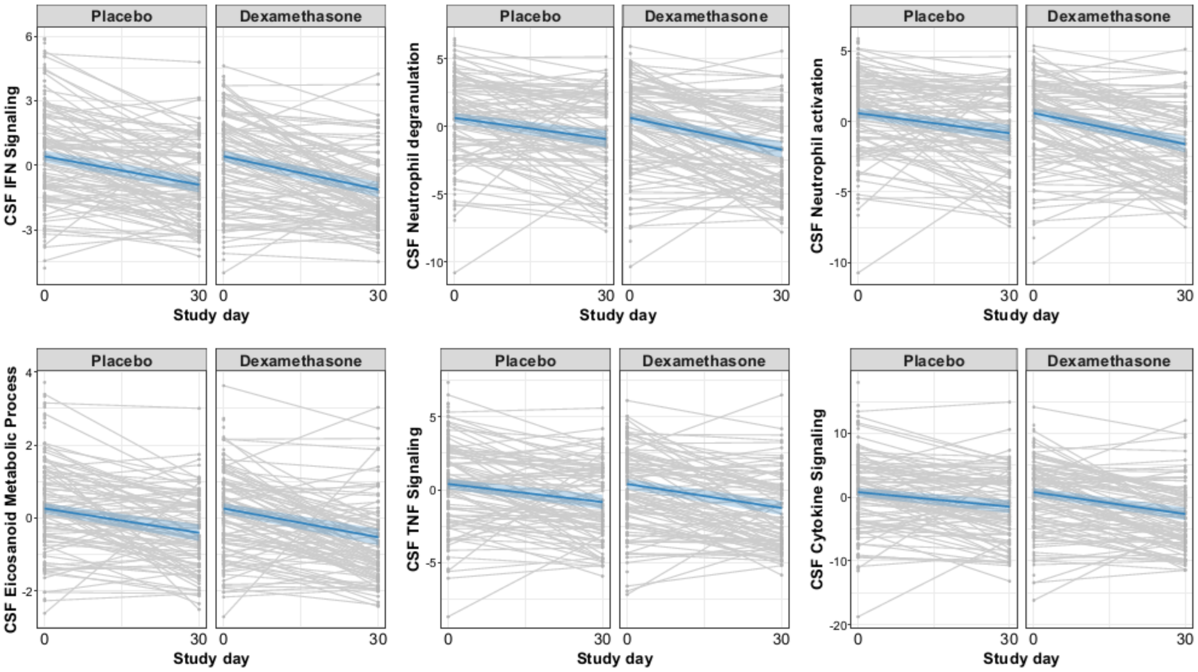

The figure shows changes in six CSF pathway activities from day 0 to day 30 in 30 in placebo or dexamethasone treated CC-genotype (a) or CT-genotype (b) participants. Pathway activity in CSF was calculated using a z-score method. Each line represents an individual participant. The solid blue lines indicate the fitted mean cytokine levels at each time point, with shaded 95% Bayesian credible intervals obtained via Markov Chain Monte Carlo (MCMC) estimation from the longitudinal sub-model of the Bayesian joint model. CSF=cerebrospinal fluid. IFN=interferon. TNF=tumour necrosis factor.

Figure S21. Comparison of change in whole blood transcriptional profiles from day 0 to 60 in placebo or dexamethasone treated CC/CT-genotype participants

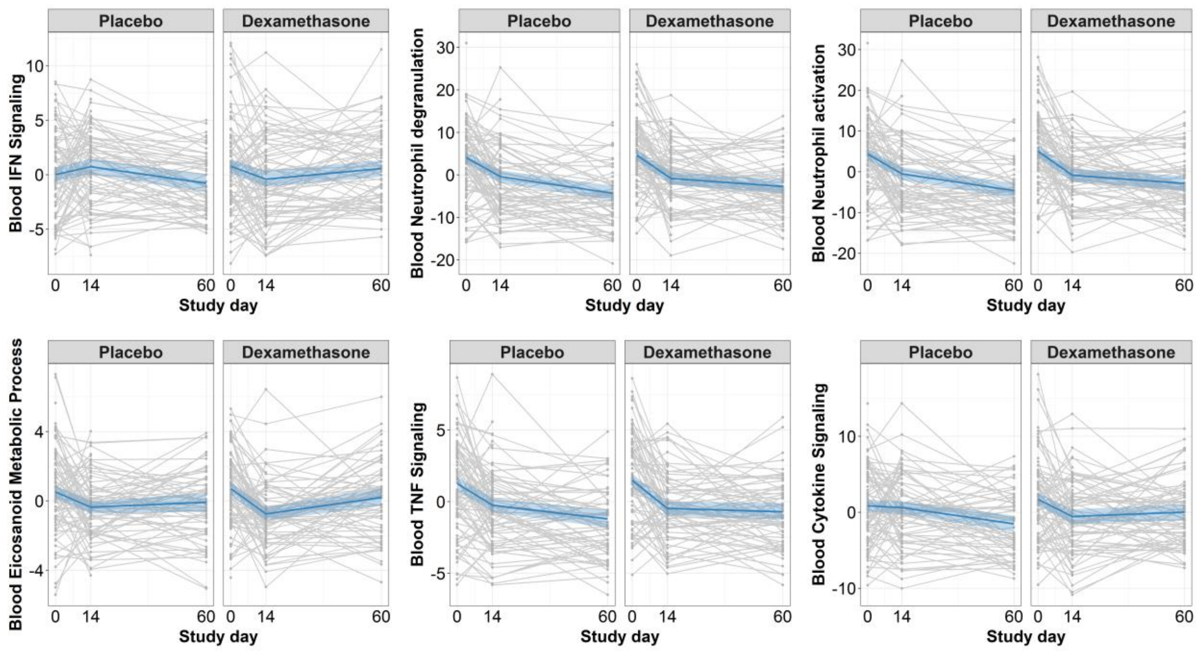

The figure shows changes in six whole-blood transcriptional pathway activities from day 0 to day 60 in placebo- or dexamethasone-treated CC/CT-genotype participants. Pathway activity in whole blood was calculated using a z-score method. Each line represents an individual participant. The solid blue lines indicate the fitted mean cytokine levels at each time point, with shaded 95% Bayesian credible intervals obtained via Markov Chain Monte Carlo (MCMC) estimation from the longitudinal sub-model of the Bayesian joint model. IFN=interferon. TNF=tumour necrosis factor.

Figure S22. Comparison of change in whole blood transcriptional profiles from day 0 to 60 in placebo or dexamethasone treated CC- genotype (a) and CT-genotype (b) participants

a)

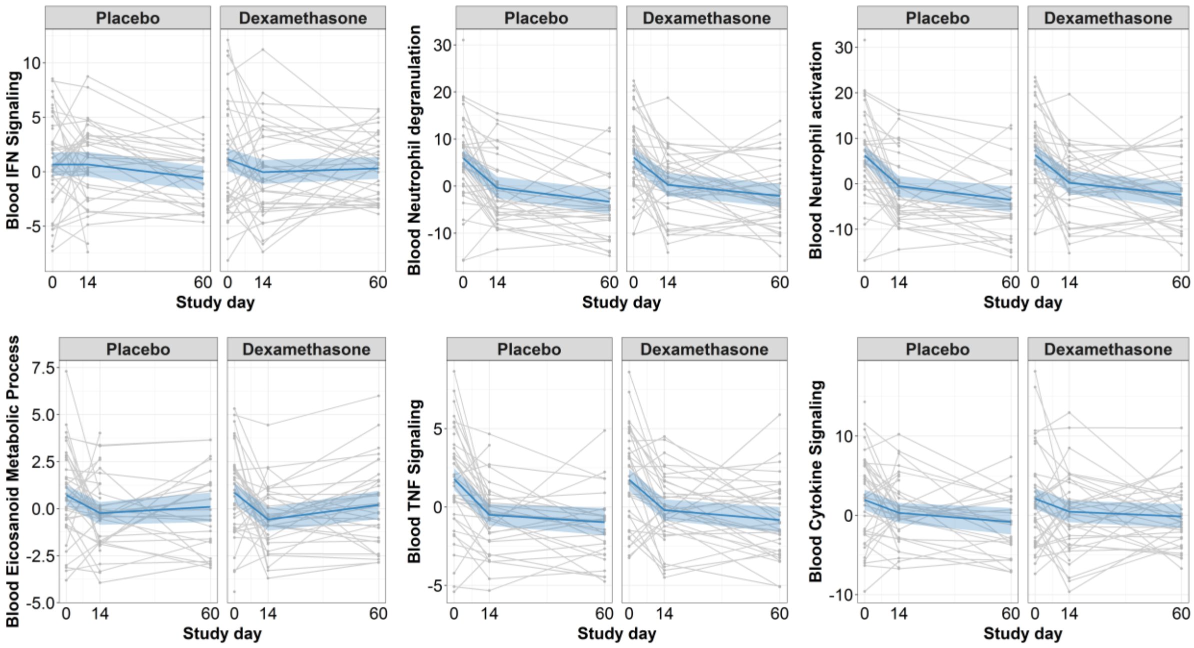

b)

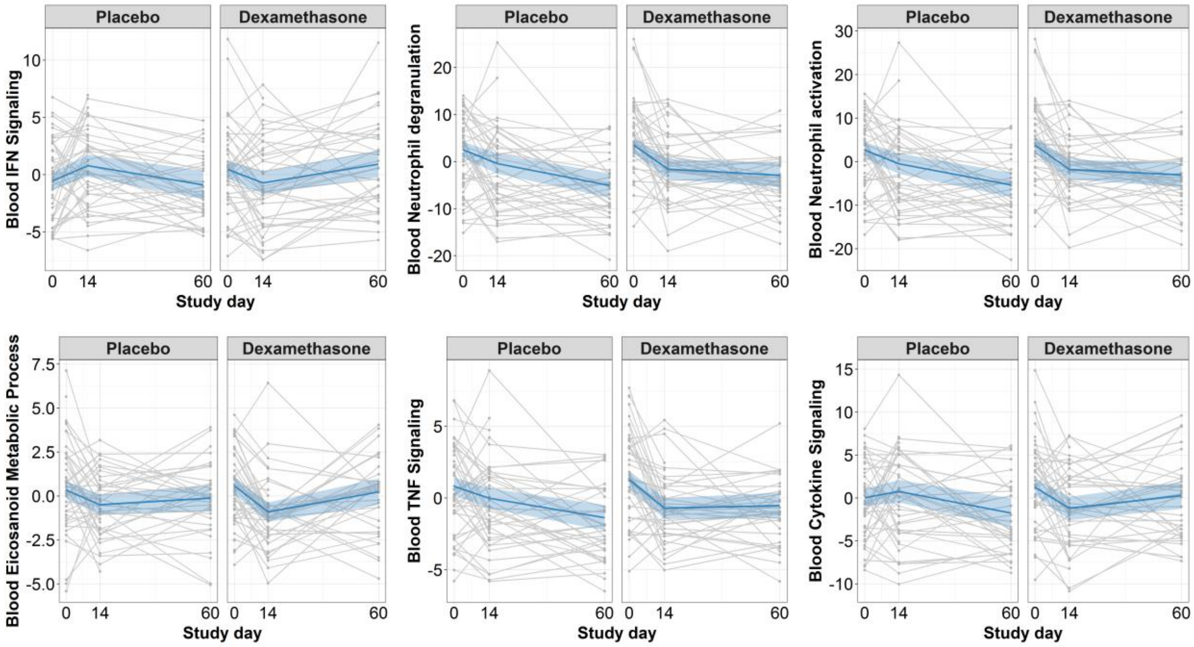

The figure shows changes in six whole-blood transcriptional pathway activities from day 0 to day 60 in in placebo or dexamethasone treated CC-genotype (a) and CT-genotype (b) participants. Pathway activity in whole blood was calculated using a z-score method. Each line represents an individual participant. The solid blue lines indicate the fitted mean cytokine levels at each time point, with shaded 95% Bayesian credible intervals obtained via Markov Chain Monte Carlo (MCMC) estimation from the longitudinal sub-model of the Bayesian joint model. IFN=interferon. TNF=tumour necrosis factor.

Figure S23. Exploratory comparison of CSF cytokines between CC and CT genotypes at day 30

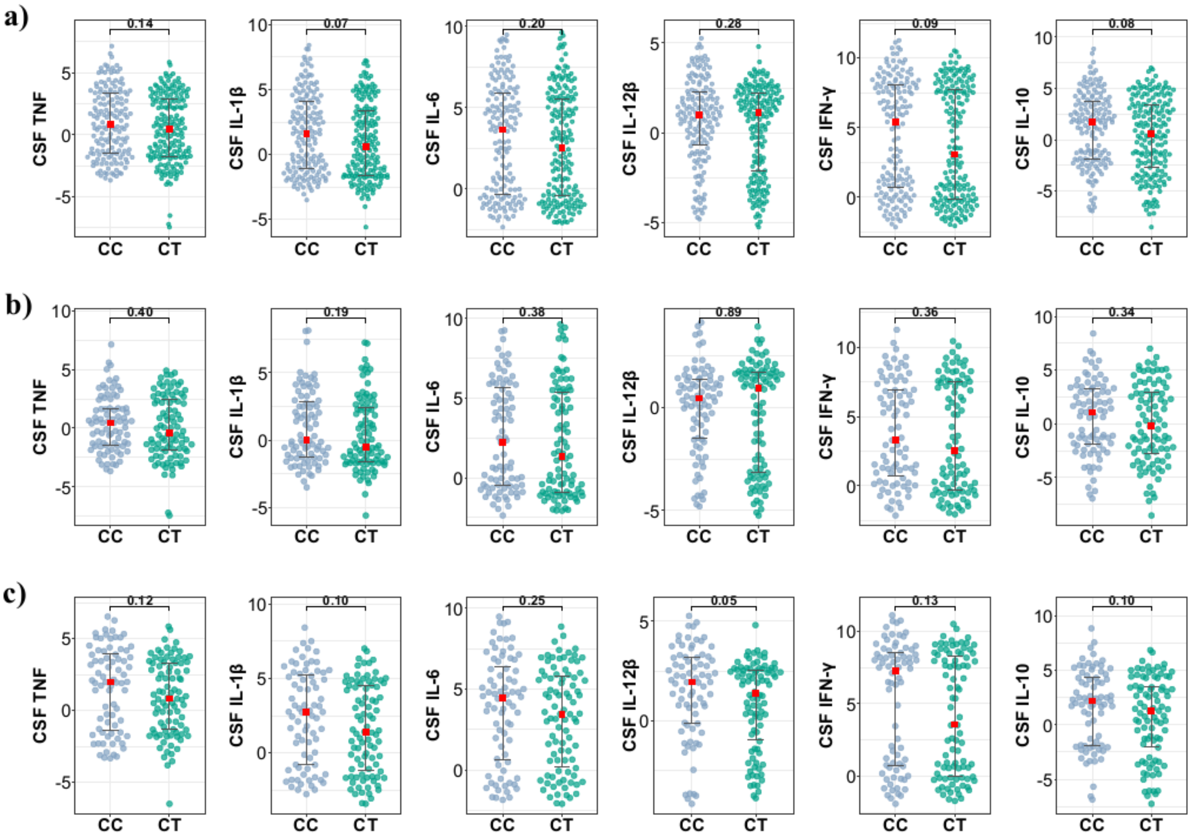

Panel (a) shows the distribution of CSF cytokine levels (NPX, log_2-normalized expression) at day 30 by CC and CT genotype among all patients with available cytokine data at this timepoint. Panel (b) presents the corresponding distribution restricted to participants in the dexamethasone arm, and Panel (c) presents the distribution restricted to participants in the placebo arm. Each subplot represents a distinct cytokine: TNF, IL-1β, IL-6, IL-12β, IFN-γ, and IL-10. The violin plots illustrate the data distribution for each genotype, with individual data points shown as dots. Each red central point indicates the median cytokine level within each group, and black horizontal bars indicate the interquartile range. Comparisons between genotype groups were performed using the Wilcoxon rank-sum test, with the corresponding p-values displayed. CSF=cerebrospinal fluid. IFN=interferon. IL=interleukin. TNF=tumour necrosis factor. This was an exploratory analysis.

Figure S24. Exploratory comparison of CSF immunopathogenesis pathways between CC and CT genotypes at day 30

a)

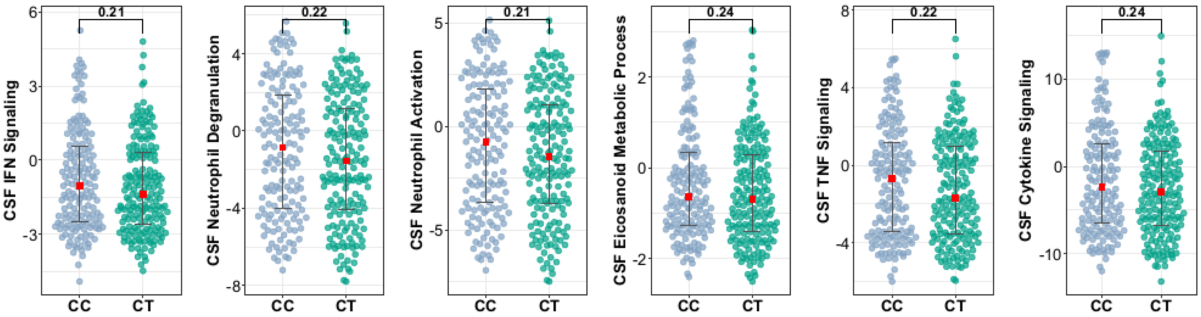

b)

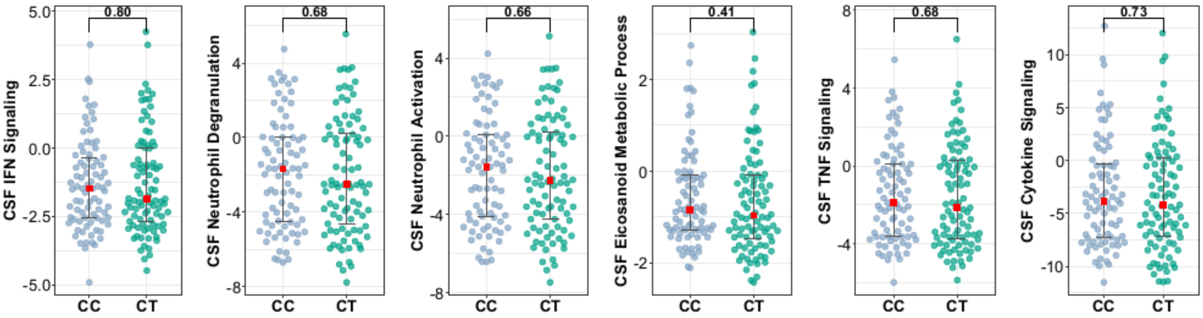

c)

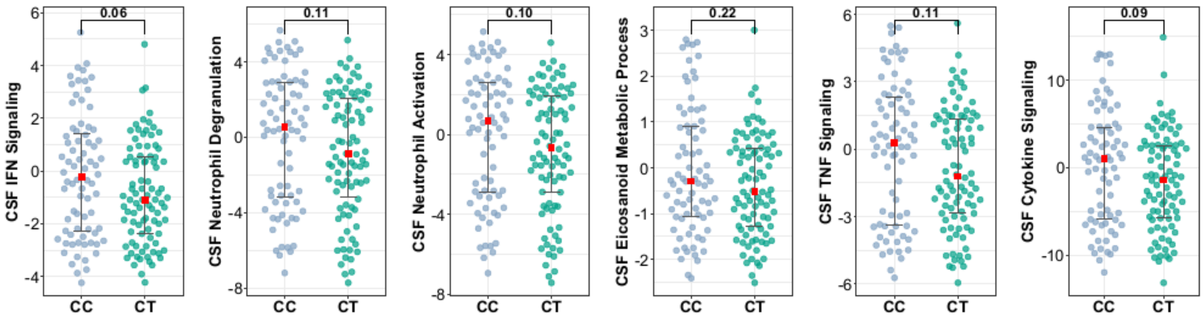

Panel (a) shows the distribution of pathway activity for six immune pathways implicated in TB/TBM pathogenesis at day 30 by CC and CT genotypes among all patients with available cytokine data at this timepoint. Panel (b) presents the corresponding distribution restricted to participants in the dexamethasone arm, and Panel (c) presents the distribution restricted to participants in the placebo arm. Pathway activity in CSF was calculated using a z-score method. Each subplot represents a distinct pathogenesis immune pathway. The violin plots illustrate the data distribution for each genotype, with individual data points shown as dots. Each red central point indicates the median cytokine level within each group, and black horizontal bars indicate the interquartile range. Comparisons between genotype groups were performed using the Wilcoxon rank-sum test, with the corresponding p-values displayed. CSF=cerebrospinal fluid. IFN=interferon. TBM=tuberculous meningitis. TNF=tumour necrosis factor. This was an exploratory analysis.

Figure S25. Individual participant meta-analysis of 9-month survival in dexamethasone and placebo treated participants in the 2004 and current trial

a) All participants (n=1,149)

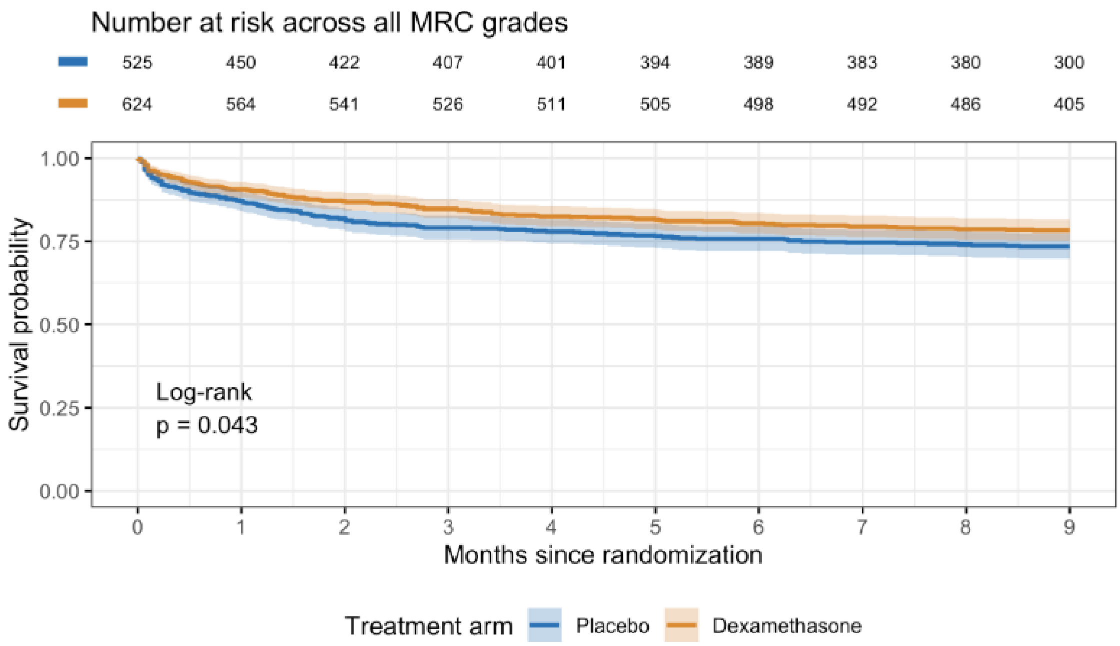

b) All participants sub-grouped by modified MRC severity grade (exploratory)

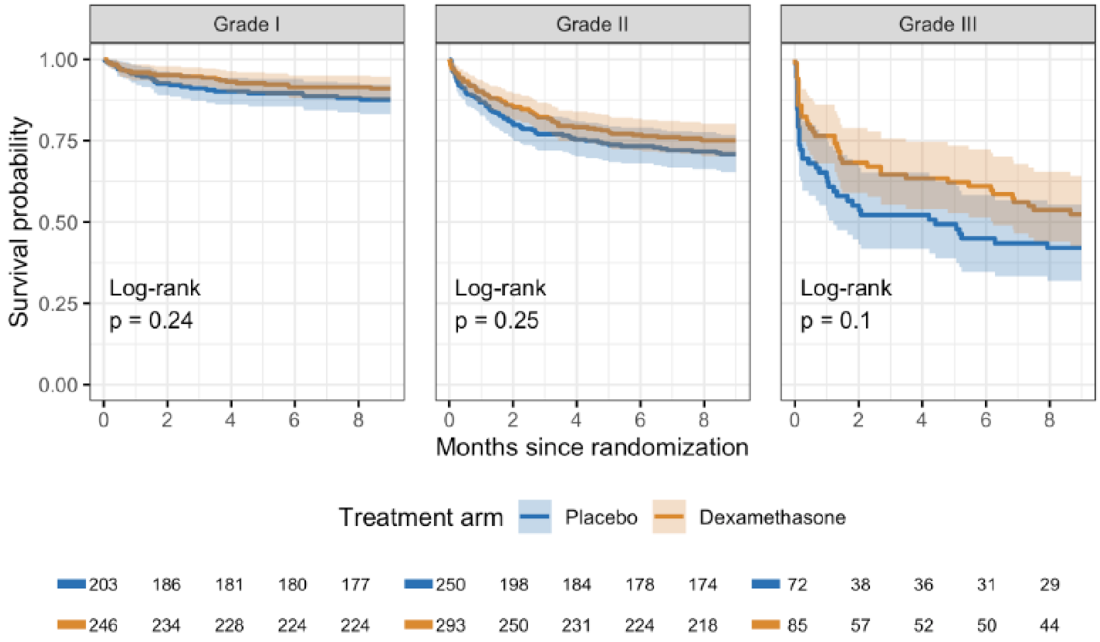

c) Survival comparison between the current and 2004 trials by treatment arm (exploratory)

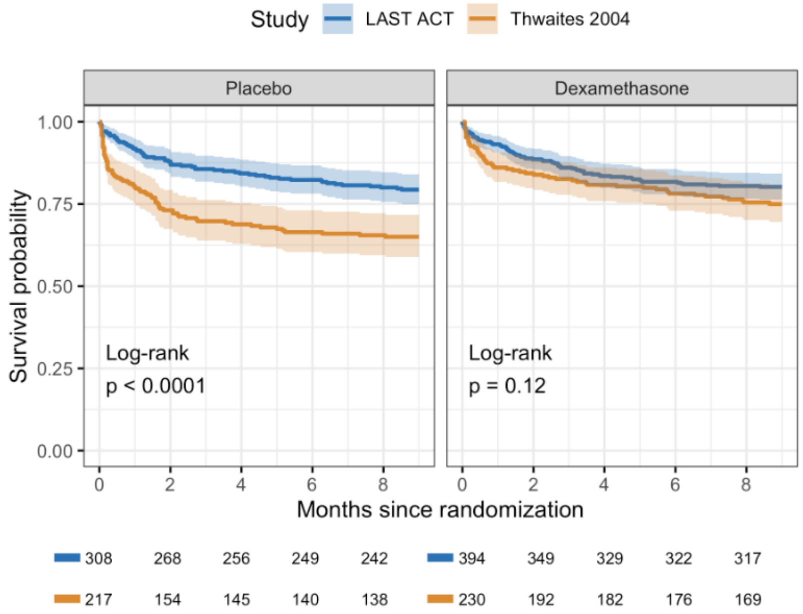

Coloured shading represents 95% confidence intervals. MRC=Medical Research Council.

Figure S26. Consort 2025 statement checklist

Section/topic	No	CONSORT 2025 checklist item description
**Title and abstract**		
Title and structured abstract	1a	Identification as a randomized trial. **Page 3**
	1b	Structured summary of the trial design, methods, results, and conclusions. **Page 3**
**Open science**		
Trial registration	2	Name of trial registry, identifying number (with URL) and date of registration. **Page 3**
Protocol and statistical analysis plan	3	Where the trial protocol and statistical analysis plan can be accessed. **Page 25**
Data sharing	4	Where and how the individual de-identified participant data (including data dictionary), statistical code and any other materials can be accessed. **Page 35-36**
Funding and conflicts of interest	5a	Sources of funding and other support (eg, supply of drugs), and role of funders in the design, conduct, analysis and reporting of the trial. **Page 16**
	5b	Financial and other conflicts of interest of the manuscript authors. **Page 16**

Section/topic	No	CONSORT 2025 checklist item description
**Introduction**		
Background and rationale	6	Scientific background and rationale. **Pages 4-6**
Objectives	7	Specific objectives related to benefits and harms. **Page 5**
**Methods**		
Patient and public involvement	8	Details of patient or public involvement in the design, conduct and reporting of the trial **-**
Trial design	9	Description of trial design including type of trial (eg, parallel group or crossover), allocation ratio, and framework (for example, superiority, equivalence, non-inferiority or exploratory). **Pages 5-6, 27**
Changes to trial protocol	10	Important changes to the trial after it commenced including any outcomes or analyses that were not prespecified, with reason. **Pages 10-11**
Trial setting	11	Settings (such as community or hospital) and locations (eg, countries or sites) where the trial was conducted. **Page 25**
Eligibility criteria	12a	Eligibility criteria for participants. **Page 25**
	12b	If applicable, eligibility criteria for sites and for individuals delivering the interventions (eg, surgeons or physiotherapists) **N/A**

Section/topic	No	CONSORT 2025 checklist item description
Intervention and comparator	13	Intervention and comparator with sufficient details to allow replication. If relevant, where additional materials describing the intervention and comparator (eg, intervention manual) can be accessed. **Pages 27-28**
Outcomes	14	Prespecified primary and secondary outcomes, including the specific measurement variable (eg, systolic blood pressure), analysis metric (for example, change from baseline, final value, time to event), method of aggregation (eg, median, proportion), and time point for each outcome. **Page 29**
Harms	15	How harms were defined and assessed (eg, systematically or non-systematically). **Pages 29-30**
Sample size	16a	How sample size was determined, including all assumptions supporting the sample size calculation. **Pages 32-33**
	16b	Explanation of any interim analyses and stopping guidelines. **Page 32**
Randomization:		
Sequence generation	17a	Who generated the random allocation sequence and the method used. **Page 27**
	17b	Type of randomization and details of any restriction (eg, stratification, blocking and block size). **Page 27**
Allocation concealment mechanism	18	Mechanism used to implement the random allocation sequence (eg, central computer/telephone; sequentially numbered, opaque, sealed containers), describing any steps to conceal the sequence until interventions were assigned. **Page 27**
Implementation	19	Whether the personnel who enrolled and those who assigned participants to the interventions had access to the random allocation sequence. **Page 27**

Section/topic	No	CONSORT 2025 checklist item description
Blinding	20a	Who was blinded after assignment to interventions (eg, participants, care providers, outcome assessors, data analysts). **Page 31.**
	20b	If blinded, how blinding was achieved and description of the similarity of interventions. **Page 29.**
Statistical methods	21a	Statistical methods used to compare groups for primary and secondary outcomes, including harms. **Pages 32-34**
	21b	Definition of who is included in each analysis (eg, all randomized participants), and in which group. **Pages 32-33**
	21c	How missing data were handled in the analysis **-**
	21 d	Methods for any additional analyses (eg. subgroup and sensitivity analyses), distinguishing prespecified from post hoc. **Pages 34**
**Results**		
Participant flow, including flow diagram	22a	For each group, the numbers of participants who were randomly assigned, received intended intervention, and were analyzed for the primary outcome. **Pages 6, 17**
	22b	For each group, losses and exclusions after randomization, together with reasons. **Pages 6, 21**
Recruitment	23a	Dates defining the periods of recruitment and follow-up for outcomes of benefits and harms. **Page 6**
	23b	If relevant, why the trial ended or was stopped **N/A**
	24a	Intervention and comparator as they were actually administered (eg, where appropriate, who delivered the intervention/comparator, how participants adhered, whether they were delivered as intended (fidelity)). **Page 9**

Section/topic	No	CONSORT 2025 checklist item description
Intervention and comparator delivery	24b	Concomitant care received during the trial for each group. **Page 27-29**
Baseline data	25	A table showing baseline demographic and clinical characteristics for each group. **Pages 17-18**
Numbers analyzed, outcomes and estimation	26	For each primary and secondary outcome, by group:<br>• the number of participants included in the analysis. **Page 19, and supplement**<br>• the number of participants with available data at the outcome time point. **Page 19, and supplement**<br>• result for each group, and the estimated effect size and its precision (such as 95% confidence interval). **Page 19, and supplement**<br>• for binary outcomes, presentation of both absolute and relative effect size. **Page 19, and supplement**
Harms	27	All harms or unintended events in each group. **Page 10 and supplement**
Ancillary analyses	28	Any other analyses performed, including subgroup and sensitivity analyses, distinguishing pre-specified from post hoc. **Page 10-11**
**Discussion**		
Interpretation	29	Interpretation consistent with results, balancing benefits and harms, and considering other relevant evidence. **Pages 12-15**
Limitations	30	Trial limitations, addressing sources of potential bias, imprecision, generalisability, and, if relevant, multiplicity of analyses. **Pages 13-14**

Supplementary tables

Table S1. Reasons and numbers excluded for the per-protocol analysis

	**CC-genotype**		**CT-genotype**		**TT-genotype**	
**Reason excluded from per-protocol analysis**	Placebo N=13/145	Dexamethasone N=6/146	Placebo N=7/163	Dexamethasone N=2/159	Placebo N=0/0	Dexamethasone N=3/89
<30 days of anti-tuberculosis drugs for any reason other than death	3 (2.1%)	3 (2.1%)	0 (0%)	1 (0.6%)	0	0
<7 days of administration of the randomized study drug for reasons other than death	10 (6.9%)	3 (2.1%)	7 (4.3%)	1 (0.6%)	0	3 (3.4%)

Table S2. Supplementary baseline data in the randomized (CC/CT-genotype) and TT-genotype participants (additional to table 1 in the main text)

Characteristic	N	All participants (N=702)	CC/CT genotype (N=613)		TT genotype (N=89)
**Treatment**			**Dexamethasone (N=305)**	**Placebo (N=308)**	**Dexamethasone (N=89)**
Site	702				
Hospital for Tropical Diseases		213 (30.3%)	90 (29.5%)	95 (30.8%)	28 (31.5%)
Pham Ngoc Thach Hospital		489 (69.7%)	215 (70.5%)	213 (69.2%)	61 (68.5%)
Previous tuberculosis treatment	702				
Yes		41 (5.8%)	17 (5.6%)	22 (7.1%)	2 (2.2%)
No		646 (92.0%)	283 (92.8%)	277 (89.9%)	86 (96.6%)
Unknown		15 (2.1%)	5 (1.6%)	9 (2.9%)	1 (1.1%)
Chest X-ray findings	701				
No tuberculosis		374 (53.3%)	155 (50.8%)	162 (52.6%)	57 (64.0%)
Miliary tuberculosis		67 (9.5%)	33 (10.8%)	27 (8.8%)	7 (7.9%)
Pulmonary tuberculosis		260 (37.0%)	117 (38.4%)	119 (38.6%)	24 (27.0%)
Neurological signs					
Urinary retention	702	171 (24.4%)	78 (25.6%)	67 (21.8%)	26 (29.2%)
History of diabetes	702				
Yes		71 (10.1%)	30 (9.8%)	32 (10.4%)	9 (10.1%)
No		608 (86.6%)	264 (86.6%)	268 (87.0%)	76 (85.4%)
Unknown		23 (3.3%)	11 (3.6%)	8 (2.6%)	4 (4.5%)
HbA1c	690	5.8 (5.4, 6.2)	5.8 (5.5, 6.2)	5.7 (5.3, 6.2)	5.7 (5.4, 6.1)
Hepatitis B sAg positivity	702	63 (9.0%)	17 (5.6%)	39 (12.7%)	7 (7.9%)
Hepatitis C Ab positivity	701				

**Characteristic**	**N**	**All participants (N=702)**	**CC/CT genotype (N=613)**		**TT genotype (N=89)**
**Treatment**			**Dexamethasone (N=305)**	**Placebo (N=308)**	**Dexamethasone (N=89)**
Positive		15 (2.1%)	8 (2.6%)	5 (1.6%)	2 (2.2%)
Negative		684 (97.6%)	296 (97.4%)	301 (97.7%)	87 (97.8%)
Not Done		2 (0.3%)	0 (0%)	2 (0.6%)	0 (0%)
Alanine aminotransferase (ALT) [IU/L]	699	27 (18, 46)	26 (18, 46)	28 (19, 46)	26 (15, 39)
Bilirubin [μmol/L]	697	14 (10, 19)	13 (10, 20)	14 (10, 19)	13 (9, 16)
Full blood count					
Hemoglobin [g/dL]	700	13 (11, 14)	13 (11, 14)	13 (11, 14)	12 (11, 14)
White cell count [x$10^3/\mu$L]	700	10 (7, 12)	10 (8, 13)	10 (7, 12)	9 (7, 12)
Platelets [x$10^3/\mu$L]	700	299 (234, 364)	304 (227, 372)	298 (234, 361)	288 (235, 340)
Plasma sodium [mmol/L]	699	131 (126, 137)	131 (126, 136)	131 (127, 137)	130 (126, 137)
CSF parameters					
Open pressure [cmH_2O]	115	18 (14, 26)	18 (13, 24)	18 (14, 26)	24 (17, 34)
Enrolment anti-tuberculosis chemotherapy regimen					
Rifampin	702	695 (99.0%)	301 (98.7%)	306 (99.4%)	88 (98.9%)
Isoniazid	702	698 (99.4%)	303 (99.3%)	307 (99.7%)	88 (98.9%)
Pyrazinamide	702	697 (99.3%)	302 (99.0%)	307 (99.7%)	88 (98.9%)
Streptomycin	702	413 (58.8%)	186 (61.0%)	173 (56.2%)	54 (60.7%)
Ethambutol	702	445 (63.4%)	195 (63.9%)	195 (63.3%)	55 (61.8%)
Anti-tuberculosis drug resistance	701				
Multi-drug resistant or rifampin mono-resistant*		8 (1.1%)	5 (1.6%)	3 (1.0%)	0 (0%)

**Characteristic**	**N**	**All participants (N=702)**	**CC/CT genotype (N=613)**		**TT genotype (N=89)**
**Treatment**			**Dexamethasone (N=305)**	**Placebo (N=308)**	**Dexamethasone (N=89)**
Isoniazid resistant (without rifampin resistance)		38 (5.4%)	18 (5.9%)	16 (5.2%)	4 (4.5%)
No or other resistance		129 (18.4%)	53 (17.4%)	54 (17.5%)	22 (24.7%)
Mtb not isolated/missing result		526 (75.0%)	228 (75.0%)	235 (76.3%)	63 (70.8%)

*Defined as positive mycobacterial culture with rifampicin and/or isoniazid resistance, or positive Xpert or Xpert Ultra with rifampicin resistance detected. N = number of participants included in that statistic. Summary statistic = the median (1st and 3rd quartile) value for continuous data, and the number and frequency (%) of participants with the characteristic for categorical data. HbA1c ≥6.5% indicates diabetes by HbA1c criteria. Ab=antibody. Ag=antigen. *Mtb=Mycobacterium tuberculosis*

Table S3. Supplementary baseline data by genotype

Characteristic	N	Overall (N=702)	CC-genotype (N= 291)	CT-genotype (N=322)	TT-genotype (N=89)
Treatment arm	702				
Placebo		308 (43.9%)	145 (49.8%)	163 (50.6%)	0 (0%)
Dexamethasone		394 (56.1%)	146 (50.2%)	159 (49.4%)	89 (100%)
Age [years]	702	47 (33, 59)	48 (36, 58)	46 (33, 60)	42 (28, 59)
Sex	702				
Female		267 (38.0%)	104 (35.7%)	126 (39.1%)	37 (41.6%)
Male		435 (62.0%)	187 (64.3%)	196 (60.9%)	52 (58.4%)
Site	702				
Hospital for Tropical Diseases		213 (30.3%)	89 (30.6%)	96 (29.8%)	28 (31.5%)
Pham Ngoc Thach Hospital		489 (69.7%)	202 (69.4%)	226 (70.2%)	61 (68.5%)
TBM diagnosis	702				
Definite TBM		312 (44.4%)	125 (43.0%)	142 (44.1%)	45 (50.6%)
Probable TBM		241 (34.3%)	99 (34.0%)	116 (36.0%)	26 (29.2%)
Possible TBM		149 (21.2%)	67 (23.0%)	64 (19.9%)	18 (20.2%)
Confirmed non-TBM		0 (0%)	0 (0%)	0 (0%)	0 (0%)
Previous tuberculosis treatment	702				
Yes		41 (5.8%)	18 (6.2%)	21 (6.5%)	2 (2.2%)
No		646 (92.0%)	265 (91.1%)	295 (91.6%)	86 (96.6%)
Unknown		15 (2.1%)	8 (2.7%)	6 (1.9%)	1 (1.1%)
Chest X-ray findings	701				
No tuberculosis		374 (53.3%)	153 (52.6%)	164 (50.9%)	57 (64.0%)
Miliary tuberculosis		67 (9.5%)	22 (7.6%)	38 (11.8%)	7 (7.9%)
Pulmonary tuberculosis		260 (37.0%)	116 (39.9%)	120 (37.3%)	24 (27.0%)
Modified MRC grade	702				
Grade I		298 (42.5%)	115 (39.5%)	151 (46.9%)	32 (36.0%)
Grade II		342 (48.7%)	148 (50.9%)	153 (47.5%)	41 (46.1%)
Grade III		62 (8.8%)	28 (9.6%)	18 (5.6%)	16 (18.0%)
Glasgow coma score (/15)	698	15 (13, 15)	14 (13, 15)	15 (13, 15)	14 (11, 15)
Duration of symptoms (days)	702	15 (12, 21)	16 (12, 22)	14 (11, 21)	16 (12, 20)
Neurological signs					
Cranial nerve palsy	702	86 (12.3%)	40 (13.7%)	35 (10.9%)	11 (12.4%)
Hemiplegia	702	76 (10.8%)	32 (11.0%)	29 (9.0%)	15 (16.9%)

Characteristic	N	Overall (N=702)	CC-genotype (N= 291)	CT-genotype (N=322)	TT-genotype (N=89)
Paraplegia/Tetraplegia	702	125 (17.8%)	60 (20.6%)	48 (14.9%)	17 (19.1%)
Urinary retention	702	171 (24.4%)	77 (26.5%)	68 (21.1%)	26 (29.2%)
History of diabetes	702				
Yes		71 (10.1%)	28 (9.6%)	34 (10.6%)	9 (10.1%)
No		608 (86.6%)	253 (86.9%)	279 (86.6%)	76 (85.4%)
Unknown		23 (3.3%)	10 (3.4%)	9 (2.8%)	4 (4.5%)
HbA1c	690	5.8 (5.4, 6.2)	5.8 (5.4, 6.3)	5.7 (5.3, 6.1)	5.7 (5.4, 6.1)
Hepatitis B sAg positivity	702	63 (9.0%)	31 (10.7%)	25 (7.8%)	7 (7.9%)
Hepatitis C Ab positivity	701				
Positive		15 (2.1%)	6 (2.1%)	7 (2.2%)	2 (2.2%)
Negative		684 (97.6%)	284 (97.6%)	313 (97.5%)	87 (97.8%)
Not Done		2 (0.3%)	1 (0.3%)	1 (0.3%)	0 (0%)
Alanine aminotransferase (ALT) [IU/L]	699	27 (18, 46)	30 (19, 48)	26 (18, 44)	26 (15, 39)
Bilirubin [μmol/L]	697	14 (10, 19)	14 (11, 21)	14 (10, 19)	13 (9, 16)
Full blood count					
Hemoglobin [g/dL]	700	13 (11, 14)	13 (12, 14)	13 (11, 14)	12 (11, 14)
White cell count [x10^3/μL]	700	10 (7, 12)	10 (8, 13)	9 (7, 12)	9 (7, 12)
Platelets [x10^3/μL]	700	299 (234, 364)	294 (232, 356)	304 (236, 370)	288 (235, 340)
Plasma sodium [mmol/L]	699	131 (126, 137)	131 (126, 136)	132 (127, 137)	130 (126, 137)
CSF parameters					
Open pressure [cmH_2O]	115	18 (14, 26)	19 (13, 26)	17 (14, 23)	24 (17, 34)
Total leucocytes [cells/mm3]	702	136 (33, 326)	137 (30, 335)	137 (38, 325)	129 (33, 294)
Total neutrophils [cells/mm3]	702	10 (0, 52)	8 (0, 49)	11 (0, 53)	11 (0, 49)
Total lymphocytes [cells/mm3]	702	104 (29, 236)	106 (28, 252)	104 (30, 218)	91 (27, 220)
Protein [g/L]	702	1.5 (0.9, 2.2)	1.4 (0.9, 2.1)	1.5 (0.9, 2.3)	1.5 (1.0, 2.2)
CSF:Blood glucose ratio	701	0.37 (0.25, 0.49)	0.37 (0.26, 0.50)	0.36 (0.25, 0.48)	0.38 (0.23, 0.48)
CSF microbiological tests					
Positive ZN stain	702	153 (21.8%)	62 (21.3%)	71 (22.0%)	20 (22.5%)
Positive GeneXpert MTB/RIF	702	102 (24.2%)	38 (22.4%)	51 (25.4%)	13 (26.0%)
Positive GeneXpert MTB/RIF Ultra	702	116 (41.7%)	48 (40.0%)	56 (46.3%)	12 (32.4%)

**Characteristic**	**N**	**Overall (N=702)**	**CC-genotype (N= 291)**	**CT-genotype (N=322)**	**TT-genotype (N=89)**
Positive mycobacterial culture	702	175 (24.9%)	67 (23.0%)	82 (25.5%)	26 (29.2%)
Duration of anti-tuberculosis chemotherapy before enrolment [days]	700	3.0 (1.0, 4.0)	3.0 (1.0, 4.0)	3.0 (1.0, 4.0)	2.0 (2.0, 4.0)
Enrolment anti-tuberculosis chemotherapy regimen					
Rifampin	702	695 (99.0%)	289 (99.3%)	318 (98.8%)	88 (98.9%)
Isoniazid	702	698 (99.4%)	290 (99.7%)	320 (99.4%)	88 (98.9%)
Pyrazinamide	702	697 (99.3%)	288 (99.0%)	321 (99.7%)	88 (98.9%)
Streptomycin	702	413 (58.8%)	173 (59.5%)	186 (57.8%)	54 (60.7%)
Ethambutol	702	445 (63.4%)	188 (64.6%)	202 (62.7%)	55 (61.8%)
Anti-tuberculosis drug resistance	701				
Multi-drug resistant or rifampin mono-resistant		8 (1.1%)	5 (1.7%)	3 (0.9%)	0 (0%)
Isoniazid resistant without rifampin resistance		38 (5.4%)	12 (4.1%)	22 (6.9%)	4 (4.5%)
No or other resistance		129 (18.4%)	50 (17.2%)	57 (17.8%)	22 (24.7%)
Mtb not isolated/missing result		526 (75.0%)	224 (77.0%)	239 (74.5%)	63 (70.8%)

N = number of participants included in that statistic. Summary statistic = the median (1st and 3rd quartile) value for continuous data, and the number and frequency (%) of participants with the characteristic for categorical data. HbA1c ≥6.5% indicates diabetes by HbA1c criteria. Ab=antibody. Ag=antigen. *Mtb=Mycobacterium tuberculosis*

Table S4. The representativeness of study participants

**Category**	**Description**
Disease under investigation	HIV-negative tuberculous meningitis
- Sex and gender	Tuberculous meningitis affects individuals of the male sex more commonly than individuals of the female sex.[2]
- Age	Young children show increased susceptibility to tuberculous meningitis.[3] In a global burden of tuberculous meningitis modelling study, the majority of tuberculous meningitis cases were reported in the 25-34 year, and 35-44-year, age groups.
- Race or ethnic group	Tuberculous meningitis occurs in all ethnic groups. The risk of developing tuberculous meningitis within an ethnic group is linked to other risks that may or may not be present for that ethnic group. These risks may include exposure to *Mycobacterium tuberculosis*, and reduced access to healthcare.
- Geography	The incidence of tuberculous meningitis is higher in countries and regions where there is a higher incidence of tuberculosis.[4]<br><br>Eight countries account for than two thirds of tuberculosis cases globally. An estimated 70% of tuberculous meningitis cases occur in Southeast Asia and Africa.[2]
Other considerations	Our study population is generally of lower disease severity (as assessed by modified MRC grade) and with a higher proportion with a definite tuberculous meningitis diagnosis, than other trials.

Overall representativeness of this trial	Our trial is well matched with those studies with which it can be compared. Age and gender data reflected other trials of tuberculous meningitis. Comparisons with the 2004 trial, conducted in the same sites in Vietnam as the current trial and with the same intervention, can be found in **table S53** below.
Methods and questions used to collect the information	Our clinical trial has been compared with a previous clinical trial of tuberculous meningitis, and descriptive non-interventional studies of HIV-negative tuberculous meningitis. We reference a 2021 global burden of tuberculous meningitis modelling study, which uses national surveillance data from Brazil, South Africa, the United Kingdom, the United States of America, and Vietnam.[2]

Table S5. Baseline characteristics of participants with and without CSF proteomic profiling

Characteristic	N	CSF proteomic N=646	Non-CSF proteomic N=56
Treatment arm	702		
Placebo		283 (43.8%)	25 (44.6%)
Dexamethasone		363 (56.2%)	31 (55.4%)
Age [years]	702	47 (33, 59)	46 (33, 60)
Sex	702		
Female		250 (38.7%)	17 (30.4%)
Male		396 (61.3%)	39 (69.6%)
Site	702		
Hospital for Tropical Diseases		198 (30.7%)	15 (26.8%)
Pham Ngoc Thach Hospital		448 (69.3%)	41 (73.2%)
LTA4H genotype	702		
CC		267 (41.3%)	24 (42.9%)
CT		297 (46.0%)	25 (44.6%)
TT		82 (12.7%)	7 (12.5%)
TBM diagnosis	702		
Definite		282 (43.7%)	30 (53.6%)
Probable		227 (35.1%)	14 (25.0%)
Possible		137 (21.2%)	12 (21.4%)
Confirmed non-TBM		0 (0%)	0 (0%)
Previous tuberculosis treatment	702		
Yes		37 (5.7%)	4 (7.1%)
No		595 (92.1%)	51 (91.1%)
Unknown		14 (2.2%)	1 (1.8%)
Chest X-ray findings	701		
No tuberculosis		338 (52.3%)	36 (64.3%)
Miliary tuberculosis		63 (9.8%)	4 (7.1%)
Pulmonary tuberculosis		244 (37.8%)	16 (28.6%)

**Characteristic**	**N**	**CSF proteomic N=646**	**Non-CSF proteomic N=56**
Modified MRC grade	702		
Grade I		284 (44.0%)	14 (25.0%)
Grade II		308 (47.7%)	34 (60.7%)
Grade III		54 (8.4%)	8 (14.3%)
Glasgow coma score (/15)	698	15 (13, 15)	14 (13, 15)
Duration of symptoms (days)	702	15 (11, 21)	18 (13, 24)
Neurological signs			
Cranial nerve palsy	702	75 (11.6%)	11 (19.6%)
Hemiplegia	702	68 (10.5%)	8 (14.3%)
Paraplegia/Tetraplegia	702	110 (17.0%)	15 (26.8%)
Urinary retention	702	153 (23.7%)	18 (32.1%)
History of diabetes	702		
Yes		62 (9.6%)	9 (16.1%)
No		563 (87.2%)	45 (80.4%)
Unknown		21 (3.3%)	2 (3.6%)
HbA1c	690	5.7 (5.3, 6.2)	6.0 (5.6, 6.3)
Hepatitis B sAg positivity	702	59 (9.1%)	4 (7.1%)
Hepatitis C Ab positivity	701		
Positive		14 (2.2%)	1 (1.8%)
Negative		630 (97.5%)	54 (96.4%)
Not Done		1 (0.2%)	1 (1.8%)
Alanine aminotransferase (ALT) [IU/L]	699	27 (18, 45)	30 (20, 53)
Bilirubin [μmol/L]	697	14 (10, 19)	14 (10, 19)
Full blood count			
Hemoglobin [g/dL]	700	13 (11, 14)	13 (11, 14)
White cell count [x$10^3/\mu$L]	700	10 (7, 12)	11 (8, 14)
Platelets [x$10^3/\mu$L]	700	297 (230, 365)	311 (259, 346)
Plasma sodium [mmol/L]	699	132 (126, 137)	130 (126, 134)

**Characteristic**	**N**	**CSF proteomic N=646**	**Non-CSF proteomic N=56**
CSF parameters			
Open pressure [cmH_2O]	115	18 (14, 25)	24 (18, 30)
Total leucocytes [cells/mm^3]	702	130 (32, 316)	190 (53, 452)
Total neutrophils [cells/mm^3]	702	11 (0, 54)	2 (0, 38)
Total lymphocytes [cells/mm^3]	702	103 (28, 227)	151 (47, 346)
Protein [g/L]	702	1.4 (0.9, 2.2)	1.7 (1.0, 2.6)
CSF:Blood glucose ratio	701	0.37 (0.26, 0.49)	0.35 (0.22, 0.46)
CSF microbiological tests			
Positive ZN stain	702	141 (21.8%)	12 (21.4%)
Positive GeneXpert MTB/RIF	421	100 (24.5%)	2 (15.4%)
Positive GeneXpert MTB/RIF Ultra	278	94 (40.0%)	22 (51.2%)
Positive mycobacterial culture	702	163 (25.2%)	12 (21.4%)
Duration of anti-tuberculosis chemotherapy before enrolment [days]	700	3 (1, 4)	3 (1, 5)
Enrolment anti-tuberculosis chemotherapy regimen			
Rifampin	702	639 (98.9%)	56 (100%)
Isoniazid	702	642 (99.4%)	56 (100%)
Pyrazinamide	702	641 (99.2%)	56 (100%)
Streptomycin	702	375 (58.0%)	38 (67.9%)
Ethambutol	702	415 (64.2%)	30 (53.6%)
Anti-tuberculosis drug resistance	701		
Multi-drug resistant or rifampin mono-resistant		6 (0.9%)	0 (0%)
Isoniazid resistant without rifampin resistance		37 (5.7%)	2 (3.6%)
No or other resistance		121 (18.7%)	9 (16.1%)
Mtb non-isolated		482 (74.6%)	44 (78.6%)

CSF=cerebrospinal fluid. MRC=Medical Research Council. *Mtb=Mycobacterium tuberculosis*. TBM=tuberculous meningitis.

Table S6. Baseline characteristics of participants with and without transcriptomics profiling

Characteristic	N	RNA-seq N = 202	Non-RNA-seq N = 500
Treatment arm	702		
Placebo		91 (45.0%)	217 (43.4%)
Dexamethasone		111 (55.0%)	283 (56.6%)
Age [years]	702	46 (33, 57)	47 (34, 59)
Sex	702		
Female		76 (37.6%)	191 (38.2%)
Male		126 (62.4%)	309 (61.8%)
Site	702		
Hospital for Tropical Diseases		78 (38.6%)	135 (27.0%)
Pham Ngoc Thach Hospital		124 (61.4%)	365 (73.0%)
LTA4H genotype	702		
CC		81 (40.1%)	210 (42.0%)
CT		96 (47.5%)	226 (45.2%)
TT		25 (12.4%)	64 (12.8%)
TBM diagnosis	702		
Definite		82 (40.6%)	230 (46.0%)
Probable		76 (37.6%)	165 (33.0%)
Possible		44 (21.8%)	105 (21.0%)
Confirmed non-TBM		0 (0%)	0 (0%)
Previous tuberculosis treatment	702		
Yes		5 (2.5%)	36 (7.2%)
No		194 (96.0%)	452 (90.4%)
Unknown		3 (1.5%)	12 (2.4%)
Chest X-ray findings	701		
No tuberculosis		99 (49.0%)	275 (55.0%)

**Characteristic**	**N**	**RNA-seq N = 202**	**Non-RNA-seq N = 500**
Miliary tuberculosis		12 (5.9%)	55 (11.0%)
Pulmonary tuberculosis		91 (45.0%)	169 (33.8%)
Modified MRC grade	702		
Grade I		91 (45.0%)	207 (41.4%)
Grade II		98 (48.5%)	244 (48.8%)
Grade III		13 (6.4%)	49 (9.8%)
Glasgow coma score	698	15 (13, 15)	15 (13, 15)
Duration of symptoms	702	14 (11, 20)	16 (12, 22)
Neurological signs			
Cranial nerve palsy	702	22 (10.9%)	64 (12.8%)
Hemiplegia	702	13 (6.4%)	63 (12.6%)
Paraplegia/Tetraplegia	702	23 (11.4%)	102 (20.4%)
Urinary retention	702	51 (25.2%)	120 (24.0%)
History of diabetes	702		
Yes		12 (5.9%)	59 (11.8%)
No		180 (89.1%)	428 (85.6%)
Unknown		10 (5.0%)	13 (2.6%)
HbA1c	690	5.6 (5.2, 6.1)	5.8 (5.4, 6.2)
Hepatitis B sAg positivity	702	16 (8%)	47 (9.4%)
Hepatitis C Ab positivity	701		
Positive		5 (2.5%)	10 (2.0%)
Negative		196 (97.0%)	488 (97.6%)
Not Done		1 (0.5%)	1 (0.2%)
Alanine aminotransferase (ALT) [IU/L]	699	30 (21, 47)	26 (17, 44)
Bilirubin [μmol/L]	697	16 (12, 22)	13 (9, 18)
Full blood count			
Hemoglobin [g/dL]	700	13 (12, 14)	13 (11, 14)

**Characteristic**	**N**	**RNA-seq**<br>**N = 202**	**Non-RNA-seq**<br>**N = 500**
White cell count [x10^3/μL]	700	9 (7, 12)	10 (8, 13)
Platelets [x10^3/μL]	700	297 (236, 359)	300 (234, 366)
Plasma sodium [mmol/L]	699	132 (126, 138)	131 (126, 136)
CSF parameters			
Open pressure [cmH_2O]	115	19 (14, 26)	18 (14, 25)
Total leucocytes [cells/mm^3]	702	143 (20, 325)	132 (40, 328)
Total neutrophils [cells/mm^3]	702	14 (0, 65)	8 (0, 47)
Total lymphocytes [cells/mm^3]	702	107 (18, 226)	103 (31, 240)
Protein [g/L]	702	1.7 (1.0, 2.4)	1.4 (0.9, 2.2)
CSF:Blood glucose ratio	701	0.39 (0.27, 0.51)	0.36 (0.24, 0.48)
CSF microbiological tests			
Positive ZN stain	702	47 (23.3%)	106 (21.2%)
Positive GeneXpert MTB/RIF	421	39 (20.9%)	63 (26.9%)
Positive GeneXpert MTB/RIF Ultra	278	5 (35.7%)	111 (42.0%)
Positive mycobacterial culture	702	51 (25.2%)	124 (24.8%)
Duration of anti-<br>tuberculosis chemotherapy before enrolment [days]	700	2 (1, 3)	3 (2, 4)
Enrolment anti-tuberculosis chemotherapy regimen			
Rifampin	702	198 (98.0%)	497 (99.4%)
Isoniazid	702	199 (99.5%)	499 (99.8%)
Pyrazinamide	702	200 (99.0%)	497 (99.4%)
Streptomycin	702	109 (54.0%)	304 (60.8%)
Ethambutol	702	145 (71.8%)	300 (60.0%)
Anti-tuberculosis drug resistance	701		
Multi-drug resistant or rifampicin mono-resistant		2 (1.0%)	4 (0.8%)
Isoniazid resistant without rifampin resistance		9 (4.5%)	30 (6.0%)
No or other resistance		39 (19.3%)	91 (18.2%)

**Characteristic**	**N**	**RNA-seq**<br>**N = 202**	**Non-RNA-seq**<br>**N = 500**
Mtb not isolated		151 (74.8%)	375 (75.0%)

CSF=cerebrospinal fluid. LTA4H=leukotriene A4 hydrolase. MRC=Medical Research Council. *Mtb=Mycobacterium tuberculosis*. TBM=tuberculous meningitis.

Table S7. Primary outcome non-inferiority and superiority analysis for ITT randomized participants

**Non-inferiority analysis**			
	**Dexamethasone**	**Placebo**	**Hazard ratio (CI)***
All participants of CT or CC genotype	108/305 (35.4%)	110/308 (35.7%)	0.99 (0.75 - 1.31) {0.69 [0.78 - 1.26] 1.42}
All participants of CC genotype	50/146 (34.2%)	56/145 (38.6%)	0.81 (0.51 - 1.29) {0.45 [0.54 - 1.21] 1.45}
**Superiority analysis**			
All participants of CT or CC genotype	108/305 (35.4%)	110/308 (35.7%)	0.99 (0.75 - 1.31) {0.69 [0.78 - 1.26] 1.42}
All participants of CC genotype	50/146 (34.2%)	56/145 (38.6%)	0.81 (0.51 - 1.29) {0.45 [0.54 - 1.21] 1.45}

*CI=confidence interval. Significance level based on the Spiessens and Debois method, with main significance level protecting the 2.5% familywise error type I and nested confidence intervals for other familywise one-sided error rate of 5% and 0.5%. For CC & CT combined, nested confidence levels are (96%), [92%] and {99.2%}. For CC, nested significance levels are (98.3%), [96.2%] and {99.7%}.

Table S8. Restricted mean time lost (RMTL) for all-cause mortality or new neurological event, corrected for genotype and MRC grade in the ITT population.

Genotype	Endpoint	Dexamethasone (n=305)	Placebo (n=308)	Comparison
		RMTL (SE, months)	RMTL (SE, months)	RMTL difference (95%CI); p-value*
CC & CT	3 months	0.56 (0.06)	0.66 (0.06)	
	6 months	1.46 (0.13)	1.59 (0.14)	
	9 months	2.45 (0.21)	2.62 (0.21)	
	12 months	3.50 (0.28)	3.69 (0.29)	-0.14 (-0.88, 0.61); p=0.72
CC	3 months	0.54 (0.08)	0.70 (0.09)	
	6 months	1.36 (0.19)	1.73 (0.20)	
	9 months	2.31 (0.29)	2.83 (0.32)	
	12 months	3.34 (0.40)	3.99 (0.44)	-0.60 (-1.72, 0.52); p=0.29

*RMTL difference (months) and p value were based on RMTL model, correcting for MRC Grade and LTA4H genotype. CI=confidence interval. LTA4H=leukotriene A4 hydrolase. RMTL=restricted mean time lost. SE=standard error.

Table S9. Restricted mean time lost (RMTL) for the primary endpoint in prespecified sub-groups in the ITT population

Subgroup	Dexamethasone (n=305)	Placebo (n=308)	Comparison	Test for heterogeneity
	RMTL (SE, months)	RMTL (SE, months)	RMTL difference (95%CI); p-value	p-value
All participants	3.50 (0.28)	3.69 (0.29)	-0.19 (-0.99, 0.62); p=0.65	
LTA4H genotype				0.29
CC	3.34 (0.40)	3.99 (0.44)	-0.65 (-1.82, 0.53); p=0.28	
CT	3.64 (0.40)	3.43 (0.39)	0.22 (-0.89, 1.32); p=0.70	
TBM diagnosis				0.59
Definite TBM	3.74 (0.45)	4.27 (0.45)	-0.53 (-1.79, 0.72); p=0.41	
Probable TBM	3.84 (0.48)	3.43 (0.48)	0.41 (-0.94, 1.76); p=0.55	
Possible TBM	2.52 (0.56)	2.81 (0.61)	-0.28 (-1.93, 1.36); p=0.73	
Modified MRC grade				0.73
Grade I	1.58 (0.33)	1.80 (0.35)	-0.21 (-1.16, 0.73); p=0.66	
Grade II	4.48 (0.42)	4.72 (0.44)	-0.24 (-1.43, 0.96); p=0.70	
Grade III	8.62 (0.98)	7.62 (1.06)	1.00 (-1.95, 3.96); p=0.51	
Anti-tuberculosis drug resistance				0.49
Multidrug resistant or rifampin mono-resistant	0.72 (0.41)	3.56 (2.91)	-2.84 (-11.45, 5.76); p=0.52	
Isoniazid resistant without rifampin resistance	3.60 (1.22)	6.38 (1.31)	-2.78 (-6.52, 0.96); p=0.15	
No or other resistance	4.63 (0.71)	4.90 (0.75)	-0.27 (-2.32, 1.79); p=0.80	
Mtb not isolated/missing result	3.25 (0.32)	3.23 (0.32)	0.02 (-0.88, 0.92); p=0.96	

RMTL and the CIs were estimated by a univariate linear regression model with pseudo-observations in each subgroup. Heterogeneity was tested by a multivariate Wald test between two models fitted on the full population, one with an interaction term between treatment arm and the subgroup covariable, and one without. No correction for multiplicity is made. CI=confidence interval. LTA4H=leukotriene A4 hydrolase. MRC=Medical Research Council. Mtb=*Mycobacterium tuberculosis*. RMTL= Restricted mean time lost. SE=standard error. TBM=tuberculous meningitis.

Table S10. Primary endpoint non-inferiority and superiority analysis in the per-protocol population

**Non-inferiority analysis**			
	**Dexamethasone**	**Placebo**	**Hazard ratio (CI)***
All participants of CT or CC genotype	103/297 (34.7%)	101/288 (35.1%)	1.00 (0.75 - 1.33)<br>{0.69 [0.78 - 1.28] 1.45}
All participants of CC genotype	47/140 (33.6%)	50/132 (37.9%)	0.81 (0.50 - 1.32)<br>{0.44 [0.53 - 1.24] 1.49}
**Superiority analysis**			
All participants of CT or CC genotype	103/297 (34.7%)	101/288 (35.1%)	1.00 (0.75 - 1.33)<br>{0.69 [0.78 - 1.28] 1.45}
All participants of CC genotype	47/140 (33.6%)	50/132 (37.9%)	0.81 (0.50 - 1.32)<br>{0.44 [0.53 - 1.24] 1.49}

*CI=confidence interval. Significance level based on the Spiessens and Debois method, with main significance level protecting the 2.5% familywise error type I and nested confidence intervals for other familywise one-sided error rate of 5% and 0.5%.
In details, for CC & CT combined, nested confidence levels are (96%), [92%] and {99.2%}.
For CC, nested significance levels are (98.3%), [96.2%] and {99.7%}.

Table S11. Primary endpoint and prespecified sub-group analysis in the per-protocol population

	Dexamethasone (n=297)	Placebo (n=288)	Comparison	Test for heterogeneity
	events/n (risk [%])	events/n (risk [%])	HR (95%CI); p-value	p-value
All participants	103/297 (34.7)	101/288 (35.1)	0.97 (0.74, 1.27); p=0.81	
LTA4H genotype				0.38
CC	47/140 (33.6)	50/132 (37.9)	0.85 (0.57, 1.27); p=0.43	
CT	56/157 (35.7)	51/156 (32.7)	1.09 (0.74, 1.59); p=0.67	
TBM diagnosis				0.56
Definite TBM	46/123 (37.4)	52/128 (40.6)	0.87 (0.58, 1.29); p=0.48	
Probable TBM	42/106 (39.6)	34/101 (33.7)	1.20 (0.76, 1.88); p=0.44	
Possible TBM	15/68 (22.1)	15/59 (25.4)	0.87 (0.43, 1.78); p=0.71	
Modified MRC grade				0.80
Grade I	20/132 (15.2)	23/128 (18.0)	0.83 (0.46, 1.51); p=0.55	
Grade II	68/146 (46.6)	63/139 (45.3)	1.00 (0.71, 1.41); p=0.99	
Grade III	15/19 (79.0)	15/21 (71.4)	1.12 (0.55, 2.30); p=0.76	
Anti-tuberculosis drug resistance				0.61
Multi-drug resistant or rifampicin mono-resistant	2/5 (40.0)	1/3 (33.3)	0.99 (0.09, 11.01); p=0.99	
Isoniazid resistant non-MDR	6/18 (33.3)	9/15 (60.0)	0.51 (0.18, 1.42); p=0.20	
No or other resistance	23/49 (46.9)	22/49 (44.9)	0.99 (0.55, 1.78); p=0.98	

	Dexamethasone (n=297)	Placebo (n=288)	Comparison	Test for heterogeneity
	**events/n (risk [%])**	**events/n (risk [%])**	**HR (95%CI); p-value**	**p-value**
Mtb not isolated/missing result	71/224 (31.7)	69/221 (31.2)	1.00 (0.72, 1.40); p=0.98	

CI=confidence interval. HR=hazard ratio. LTA4H=leukotriene A4 hydrolase. MRC=Medical Research Council. Mtb=*Mycobacterium tuberculosis*. TBM=tuberculous meningitis.

Table S12. Restricted mean time lost (RMTL) for all-cause mortality or new neurological event, corrected for genotype and MRC grade, in the per-protocol population

Genotype	Endpoint	Dexamethasone (n=297)	Placebo (n=288)	Comparison
		RMTL (SE, months)	RMTL (SE, months)	RMTL difference (95%CI); p-value
CC & CT	3 months	0.54 (0.06)	0.62 (0.06)	
	6 months	1.41 (0.13)	1.52 (0.14)	
	9 months	2.37 (0.21)	2.52 (0.22)	
	12 months	3.39 (0.28)	3.56 (0.30)	-0.13 (-0.88, 0.63); p=0.74
CC	3 months	0.52 (0.09)	0.65 (0.09)	
	6 months	1.31 (0.19)	1.62 (0.21)	
	9 months	2.23 (0.30)	2.68 (0.33)	
	12 months	3.23 (0.41)	3.79 (0.45)	-0.58 (-1.71, 0.56); p=0.32

CI=confidence interval. MRC=Medical Research Council. RMTL=restricted mean time lost. SE=standard error.

Table S13. Absolute event risk by genotype and treatment arm

	**Death or new neurological event until 12 months after randomization**	
	**No**	**Yes**
LTA4H		
CC Dexamethasone (n=146)	96 (65.8%)	50 (34.2%)
CC Placebo (n=145)	89 (61.4%)	56 (38.6%)
CC&CT Dexamethasone (n=305)	197 (64.6%)	108 (35.4%)
CC&CT Placebo (n=308)	198 (64.3%)	110 (35.7%)
CT Dexamethasone (n=159)	101 (63.5%)	58 (36.5%)
CT Placebo (n=163)	109 (66.9%)	54 (33.1%)
TT (n=89)	61 (68.5%)	28 (31.5%)

LTA4H=leukotriene A4 hydrolase. n = number of participants included in that statistic.

Table S14. Overall incidence of death over the first 12 months after randomization in the ITT population

Genotype	Endpoint	Dexamethasone (n=305)	Placebo (n=308)	Comparison	
		events/n (risk [%])	events/n (risk [%])	HR (95%CI); p-value	RMTL difference (95%CI); p-value
CC & CT	3 months	45/305 (14.8)	44/308 (14.3)		
	6 months	58/305 (19.0)	54/308 (17.5)		
	9 months	60/305 (19.7)	63/308 (20.5)		
	12 months	66/305 (21.6)	66/308 (21.4)	1.07 (0.76, 1.51); p=0.71	0.08 (-0.52, 0.67); p=0.80
CC	3 months	18/146 (12.3)	24/145 (16.6)		
	6 months	22/146 (15.1)	29/145 (20.0)		
	9 months	24/146 (16.4)	33/145 (22.8)		
	12 months	26/146 (17.8)	34/145 (23.5)	0.74 (0.44, 1.23); p=0.24	-0.53 (-1.41, 0.36); p=0.25

CI=confidence interval. HR=hazard ratio. ITT=intention-to-treat. RMTL=restricted mean time lost.

Table S15. Death overall and in prespecified subgroups in the ITT population.

Subgroup	Dexamethasone (n=305)	Placebo (n=308)	Comparison	Test for heterogeneity
	events/n (risk [%])	events/n (risk [%])	HR (95%CI); p-value	p-value
All participants	66/305 (21.6)	66/308 (21.4)	1.00 (0.71, 1.41); p=1.0	
Leukotriene A4 hydrolase				0.09
- CC	26/146 (17.8)	34/145 (23.5)	0.72 (0.43, 1.20); p=0.21	
- CT	40/159 (25.2)	32/163 (19.6)	1.32 (0.83, 2.10); p=0.24	
Modified MRC grade				0.41
- Grade I	11/134 (8.2)	7/132 (5.3)	1.58 (0.61, 4.06); p=0.35	
- Grade II	40/150 (26.7)	44/151 (29.1)	0.88 (0.57, 1.35); p=0.55	
- Grade III	15/21 (71.4)	15/25 (60.0)	1.29 (0.63, 2.64); p=0.49	
TBM diagnosis				0.73
- Definite	22/128 (17.2)	27/139 (19.4)	0.86 (0.49, 1.50); p=0.59	
- Probable	31/107 (29.0)	30/108 (27.8)	1.04 (0.63, 1.71); p=0.89	
- Possible	13/70 (18.6)	9/61 (14.8)	1.29 (0.55, 3.01); p=0.56	

CI=confidence interval. MRC=Medical Research Council. TBM=tuberculous meningitis.

Table S16. Death restricted mean time lost (RMTL) in the ITT population

Subgroup	Dexamethasone (n=305)	Placebo (n=308)	Comparison	Test for heterogeneity
	RMTL (SE, months)	RMTL (SE, months)	RMTL difference (95%CI); p-value	p-value
All participants	2.02 (0.23)	2.00 (0.23)	0.02 (-0.63, 0.66); p=0.95	
Leukotriene A4 hydrolase				0.09
- CC	1.66 (0.31)	2.23 (0.35)	-0.57 (-1.50, 0.36); p=0.23	
- CT	2.34 (0.34)	1.80 (0.30)	0.55 (-0.35, 1.45); p=0.23	
Modified MRC grade				0.41
- Grade I	0.76 (0.23)	0.45 (0.18)	0.31 (-0.26, 0.88); p=0.29	
- Grade II	2.39 (0.35)	2.72 (0.37)	-0.33 (-1.33, 0.67); p=0.52	
- Grade III	7.35 (1.06)	5.98 (1.03)	1.38 (-1.66, 4.41); p=0.37	
TBM diagnosis				0.74
- Definite TBM	1.52 (0.32)	1.75 (0.32)	-0.23 (-1.12, 0.66); p=0.61	
- Probable TBM	2.73 (0.44)	2.62 (0.43)	0.11 (-1.11, 1.33); p=0.87	
- Possible TBM	1.83 (0.48)	1.45 (0.46)	0.38 (-0.93, 1.70); p=0.57	

CI=confidence interval. MRC=Medical Research Council. RMTL=restricted mean time lost. SE=standard error. TBM=tuberculous meningitis.

Table S17. Overall risk of first neurological event over the first 12 months after randomization in the ITT population

Genotype	Endpoint	Dexamethasone (n=305)	Placebo (n=308)	Comparison	
		events/n (risk [%])	events/n (risk [%])	HR (95%CI); p-value	RMTL difference (95%CI); p-value
CC & CT	3 months	69/305 (22.6)	74/308 (22.4)		
	6 months	83/305 (27.2)	82/308 (26.6)		
	9 months	91/305 (29.8)	85/308 (27.6)		
	12 months	93/305 (30.5)	89/308 (28.9)	1.03 (0.77, 1.38); p=0.85	0.03 (-0.71, 0.78); p=0.93
CC	3 months	7/146 (4.8)	6/145 (4.1)		
	6 months	9/146 (6.2)	9/145 (6.2)		
	9 months	14/146 (9.6)	15/145 (10.3)		
	12 months	45/146 (30.8)	49/145 (33.8)	0.83 (0.55, 1.24); p=0.35	-0.46 (-1.57, 0.65); p=0.42

CI=confidence interval. ITT=intention-to-treat. RMTL=restricted mean time lost.

Table S18. New neurological events in prespecified sub-groups in the ITT population

Subgroup	Dexamethasone (n=305)	Placebo (n=308)	Comparison	Test for heterogeneity
	events/n (risk [%])	events/n (risk [%])	HR (95%CI); p-value	p-value
All participants	93/305 (30.5)	89/308 (28.9)	1.02 (0.76, 1.37); p=0.89	
Leukotriene A4 hydrolase				0.23
- CC	45/146 (30.8)	49/145 (33.8)	0.86 (0.57, 1.29); p=0.46	
- CT	48/159 (30.2)	40/163 (24.5)	1.22 (0.80, 1.86); p=0.35	
Modified MRC grade				0.46
- Grade I	18/134 (13.4)	23/132 (17.4)	0.77 (0.41, 1.42); p=0.40	
- Grade II	62/150 (41.3)	55/151 (36.4)	1.08 (0.75, 1.55); p=0.69	
- Grade III	13/21 (61.9)	11/25 (44.0)	1.35 (0.60, 3.01); p=0.47	
TBM diagnosis				0.26
- Definite	44/128 (34.4)	52/139 (37.4)	0.86 (0.57, 1.28); p=0.45	
- Probable	35/107 (32.7)	24/108 (22.2)	1.47 (0.88, 2.48); p=0.14	
- Possible	14/70 (20.0)	13/61 (21.3)	0.94 (0.44, 2.01); p=0.88	

CI=confidence interval. HR=hazard ratio. ITT=intention-to-treat. MRC=Medical Research Council. TBM=tuberculous meningitis.

Table S19. Restricted mean time lost (RMTL) to new neurological events in the ITT population, with death as a competing risk

Subgroup	Dexamethasone (n=305)	Placebo (n=308)	Comparison	Test for heterogeneity
	RMTL (SE, months)	RMTL (SE, months)	RMTL difference (95%CI); p-value	p-value
All participants	3.03 (0.27)	3.03 (0.28)	0.01 (-0.76, 0.78); p=0.98	
LTA4H genotype				0.24
- CC	3.03 (0.39)	3.51 (0.43)	-0.49 (-1.63, 0.66); p=0.40	
- CT	3.04 (0.38)	2.60 (0.37)	0.44 (-0.60, 1.48); p=0.40	
Modified MRC grade				0.59
- Grade I	1.40 (0.31)	1.71 (0.34)	-0.31 (-1.22, 0.60); p=0.50	
- Grade II	4.00 (0.41)	3.83 (0.43)	0.17 (-1.00, 1.34); p=0.77	
- Grade III	6.48 (1.16)	5.21 (1.17)	1.27 (-2.10, 4.65); p=0.46	
TBM diagnosis				0.33
- Definite	3.46 (0.44)	3.96 (0.45)	-0.51 (-1.75, 0.74); p=0.43	
- Probable	3.06 (0.45)	2.25 (0.43)	0.81 (-0.41, 2.03); p=0.19	
- Possible	2.21 (0.53)	2.26 (0.56)	-0.05 (-1.60, 1.49); p=0.95	

CI=confidence interval. ITT=intention-to-treat. LTA4H=leukotriene A4 hydrolase. MRC=Medical Research Council. RMTL=restricted mean time lost. SE=standard error. TBM=tuberculous meningitis

Table S20. Overall risk of first use of open-label corticosteroid over the first 12 months after randomization in the ITT population

Genotype	Endpoint	Dexamethasone (n=305)	Placebo (n=308)	Comparison	
		events/n (risk [%])	events/n (risk [%])	HR (95%CI); p-value	RMTL difference (95%CI); p-value
CC & CT	3 months	45/305 (14.8)	43/308 (14.0)		
	6 months	51/305 (16.7)	47/308 (15.3)		
	9 months	52/305 (17.1)	49/308 (15.9)		
	12 months	54/305 (17.7)	50/308 (16.2)	1.06 (0.72, 1.57); p=0.75	0.12 (-0.50, 0.74); p=0.70
CC	3 months	21/146 (14.4)	21/145 (14.5)		
	6 months	24/146 (16.4)	24/145 (16.6)		
	9 months	24/146 (16.4)	25/145 (17.2)		
	12 months	25/146 (17.1)	26/145 (17.9)	0.88 (0.51, 1.52); p=0.65	-0.10 (-1.01, 0.82); p=0.84

CI=confidence interval. HR=hazard ratio. ITT=intention-to-treat. RMTL=restricted mean time lost.

Table S21. First use of open label corticosteroids in the ITT population

Subgroup	Dexamethasone (n=305)	Placebo (n=308)	Comparison	Test for heterogeneity
	events/n (risk [%])	events/n (risk [%])	HR (95%CI); p-value	p-value
All participants	54/305 (17.7)	50/308 (16.2)	1.06 (0.72, 1.56); p=0.75	
LTA4H genotype				0.41
- CC	25/146 (17.1)	26/145 (17.9)	0.90 (0.52, 1.55); p=0.70	
- CT	29/159 (18.2)	24/163 (14.7)	1.25 (0.72, 2.14); p=0.43	
Modified MRC grade				0.13
- Grade I	19/134 (14.2)	15/132 (11.4)	1.27 (0.65, 2.50); p=0.49	
- Grade II	27/150 (18.0)	31/151 (20.5)	0.81 (0.48, 1.36); p=0.43	
- Grade III	8/21 (38.1)	4/25 (16.0)	2.63 (0.79, 8.77); p=0.12	
TBM diagnosis				0.20
- Definite	32/128 (25.0)	32/139 (23.0)	1.00 (0.61, 1.63); p=0.99	
- Probable	17/107 (15.9)	10/108 (9.3)	1.77 (0.81, 3.86); p=0.15	
- Possible	5/70 (7.1)	8/61 (13.1)	0.53 (0.17, 1.62); p=0.27	

CI=confidence interval. HR=hazard ratio. ITT=intention-to-treat. LTA4H=leukotriene A4 hydrolase. MRC=Medical Research Council. TBM=tuberculous meningitis.

Table S22. Restricted mean time lost (RMTL) to new use of open label corticosteroids in prespecified sub-groups in the ITT population, with death as competing risk

Subgroup	Dexamethasone (n=305)	Placebo (n=308)	Comparison	Test for heterogeneity
	RMTL (SE, months)	RMTL (SE, months)	RMTL difference (95%CI); p-value	p-value
All participants	1.78 (0.22)	1.67 (0.22)	0.10 (-0.52, 0.72); p=0.75	
LTA4H genotype				0.54
- CC	1.71 (0.32)	1.82 (0.33)	-0.10 (-1.01, 0.81); p=0.82	
- CT	1.84 (0.32)	1.55 (0.30)	0.29 (-0.57, 1.14); p=0.51	
Modified MRC grade				0.21
- Grade I	1.38 (0.30)	1.08 (0.27)	0.29 (-0.51, 1.10); p=0.48	
- Grade II	1.81 (0.32)	2.17 (0.35)	-0.36 (-1.30, 0.59); p=0.46	
- Grade III	4.10 (1.15)	1.87 (0.85)	2.23 (-0.70, 5.16); p=0.14	
TBM diagnosis				0.40
- Definite	2.56 (0.39)	2.47 (0.39)	0.09 (-1.00, 1.18); p=0.87	
- Probable	1.54 (0.37)	0.98 (0.30)	0.56 (-0.37, 1.49); p=0.24	
- Possible	0.71 (0.31)	1.09 (0.39)	-0.38 (-1.37, 0.60); p=0.45	

CI=confidence interval. HR=hazard ratio. ITT=intention-to-treat. LTA4H=leukotriene A4 hydrolase. MRC=Medical Research Council. RMTL=restricted mean time lost. TBM=tuberculous meningitis.

Table S23. Overall risk of first use of open-label corticosteroids over the first 12 months after randomization in the ITT population

Genotype	Endpoint	Dexamethasone (n=305)	Placebo (n=308)	Comparison	
		events/n (risk [%])	events/n (risk [%])	HR (95%CI); p-value	RMTL difference (95%CI); p-value
CC & CT	3 months	26/305 (8.5)	19/308 (6.2)		
	6 months	27/305 (8.9)	21/308 (6.8)		
	9 months	27/305 (8.9)	23/308 (7.5)		
	12 months	28/305 (9.2)	24/308 (7.8)	1.18 (0.68, 2.05); p=0.56	0.21 (-0.26, 0.67); p=0.38
CC	3 months	12/146 (8.2)	8/145 (5.5)		
	6 months	12/146 (8.2)	10/145 (6.9)		
	9 months	12/146 (8.2)	11/145 (7.6)		
	12 months	12/146 (8.2)	12/145 (8.3)	0.94 (0.42, 2.09); p=0.87	0.14 (-0.52, 0.80); p=0.67

CI=confidence interval. HR=hazard ratio. ITT=intention-to-treat. RMTL=restricted mean time lost.

Table S24. Prespecified sub-group analysis of first use of open label corticosteroids in the ITT population

Subgroup	Dexamethasone (n=305)	Placebo (n=308)	Comparison	Test for heterogeneity
	events/n (risk [%])	events/n (risk [%])	HR (95%CI); p-value	p-value
All participants	28/305 (9.2)	24/308 (7.8)	1.13 (0.66, 1.95); p=0.66	
LTA4H genotype				0.53
- CC	12/146 (8.2)	12/145 (8.3)	0.94 (0.42, 2.09); p=0.88	
- CT	16/159 (10.1)	12/163 (7.4)	1.32 (0.63, 2.80); p=0.46	
Modified MRC grade				0.11
- Grade I	10/134 (7.5)	11/132 (8.3)	0.88 (0.38, 2.08); p=0.78	
- Grade II	13/150 (8.7)	12/151 (8.0)	1.01 (0.46, 2.21); p=0.99	
- Grade III	5/21 (23.8)	1/25 (4.0)	6.76 (0.79, 58.14); p=0.082	
TBM diagnosis				0.09
- Definite	16/128 (12.5)	12/139 (8.6)	1.30 (0.61, 2.75); p=0.49	
- Probable	11/107 (10.3)	7/108 (6.5)	1.61 (0.63, 4.17); p=0.32	
- Possible	1/70 (1.4)	5/61 (8.2)	0.16 (0.02, 1.41); p=0.10	

CI=confidence interval. HR=hazard ratio. ITT=intention-to-treat. LTA4H=leukotriene A4 hydrolase. MRC=Medical Research Council. TBM=tuberculous meningitis.

Table S25. Restricted mean time lost (RMTL) to first use of open label corticosteroids in prespecified subgroups in the ITT population

Subgroup	Dexamethasone (n=305)	Placebo (n=308)	Comparison	Test for heterogeneity
	RMTL (SE, months)	RMTL (SE, months)	RMTL difference (95%CI); p-value	p-value
All participants	0.96 (0.18)	0.76 (0.16)	0.20 (-0.26, 0.66); p=0.40	
LTA4H				0.80
- CC	0.89 (0.25)	0.75 (0.22)	0.13 (-0.52, 0.79); p=0.69	
- CT	1.03 (0.25)	0.77 (0.22)	0.26 (-0.40, 0.91); p=0.44	
Modified MRC grade				0.17
- Grade I	0.73 (0.23)	0.77 (0.23)	-0.04 (-0.69, 0.61); p=0.91	
- Grade II	0.93 (0.25)	0.81 (0.23)	0.13 (-0.54, 0.80); p=0.71	
- Grade III	2.61 (1.02)	0.46 (0.45)	2.15 (-0.15, 4.45); p=0.07	
TBM diagnosis				0.18
- Definite	1.31 (0.31)	0.89 (0.25)	0.42 (-0.37, 1.21); p=0.30	
- Probable	1.05 (0.31)	0.69 (0.25)	0.36 (-0.44, 1.15); p=0.38	
- Possible	0.17 (0.17)	0.58 (0.28)	-0.41 (-1.06, 0.23); p=0.21	

CI=confidence interval. ITT=intention-to-treat. LTA4H=leukotriene A4 hydrolase. MRC=Medical Research Council. RMTL=restricted mean time lost. SE=standard error. TBM=tuberculous meningitis.

Table S26. Total numbers given open-label corticosteroid within 12 months, by subgroups in the ITT population

	Dexamethasone (N=394)		Placebo (N=308)	
**Type of adverse event**	**n episode**	**n participants**	**n episode**	**n participants**
All participants	80	69/394 (17.5%)	55	50/308 (16.2%)
LTA4H genotype	80	69/394 (17.5%)	55	50/308 (16.2%)
CC	30	25/394 (6.3%)	29	26/308 (8.4%)
CT	33	29/394 (7.4%)	26	24/308 (7.8%)
TT	17	15/394 (3.8%)	0	0/308 (0%)
Modified MRC grade	80	69/394 (17.5%)	55	50/308 (16.2%)
Grade I	25	23/394 (5.8%)	15	15/308 (4.9%)
Grade II	40	33/394 (8.4%)	35	31/308 (10.1%)
Grade III	15	13/394 (3.3%)	5	4/308 (1.3%)
TBM diagnosis	80	69/394 (17.5%)	55	50/308 (16.2%)
Definite	49	41/394 (10.4%)	35	32/308 (10.4%)
Possible	9	7/394 (1.8%)	8	8/308 (2.6%)
Probable	22	21/394 (5.3%)	12	10/308 (3.2%)

n episode refers to the number of events in each study arm. n participants refers to the number of participants with at least one event in each study arm.

Table S27. Summary of reasons to use open-label corticosteroid treatment over the first 12 months after randomization in the ITT population

Note that one episode of open-label corticosteroid can have more than one reason.

		Dexamethasone (N=394)			Placebo (N=308)	
**Reason**	**n episode**	**n participants**	**Days from randomization until use of open-label corticosteroids**	**n episode**	**n participants**	**Days from randomization until use of open-label corticosteroids**
- Depressed level of consciousness	11	11/394 (3%)	47 (29-70)	10	10/308 (3%)	36 (18-80)
- Seizure	1	1/394 (0%)	194 (194-194)	5	2/308 (1%)	100 (98-107)
- Respiratory failure	5	5/394 (1%)	43 (41-115)	0	0/308 (0%)	-
- Hemiplegia	5	3/394 (1%)	76 (48-98)	0	0/308 (0%)	-
- Dyspnoea	4	4/394 (1%)	48.5 (27-80)	1	1/308 (0%)	9 (9-9)
- Allergic reaction to TB drugs	2	2/394 (1%)	7 (5-8)	3	3/308 (1%)	148 (101-199)
- Adrenal suppression	0	0/394 (0%)	-	5	4/308 (1%)	36 (18-80)
- Tuberculoma	3	2/394 (1%)	56 (48-62)	1	1/308 (0%)	44 (44-44)
- Worsening CSF inflammation	1	1/394 (0%)	69 (69-69)	3	2/308 (1%)	36 (33-39)
- Headache	3	2/394 (1%)	68 (53-86)	0	0/308 (0%)	-
- Hydrocephalus	1	1/394 (0%)	32 (32-32)	2	2/308 (1%)	23 (15-30)
- Clinician's discretion	0	0/394 (0%)	-	3	3/308 (1%)	36 (21-36)
- Blurred vision	2	2/394 (1%)	176 (113-240)	0	0/308 (0%)	-
- Allergic reaction	2	2/394 (1%)	19 (15-22)	0	0/308 (0%)	-
- Severe headache	2	2/394 (1%)	33 (26-39)	0	0/308 (0%)	-
- Blurred vision, brain inflammation	2	1/394 (0%)	216 (199-232)	0	0/308 (0%)	-
- Acute asthma	2	1/394 (0%)	11 (9-12)	0	0/308 (0%)	-
- Used to treat drug resistant disease	2	1/394 (0%)	42 (38-46)	0	0/308 (0%)	-
- Sepsis	1	1/394 (0%)	40 (40-40)	1	1/308 (0%)	268 (268-268)

		**Dexamethasone (N=394)**			**Placebo (N=308)**	
**Reason**	**n episode**	**n participants**	**Days from randomization until use of open-label corticosteroids**	**n episode**	**n participants**	**Days from randomization until use of open-label corticosteroids**
- Worsening tuberculous meningitis	1	1/394 (0%)	51 (51-51)	1	1/308 (0%)	46 (46-46)
- Paraplegia	1	1/394 (0%)	122 (122-122)	0	0/308 (0%)	-
- Headache, vomiting, new tuberculoma	1	1/394 (0%)	53 (53-53)	0	0/308 (0%)	-
- Headache and tuberculoma	1	1/394 (0%)	60 (60-60)	0	0/308 (0%)	-
- Headache, vomiting, new lesions	1	1/394 (0%)	70 (70-70)	0	0/308 (0%)	-
- Hydrocephalus, tuberculoma	1	1/394 (0%)	25 (25-25)	0	0/308 (0%)	-
- Clinician's discretion given slowly improving CSF	1	1/394 (0%)	60 (60-60)	0	0/308 (0%)	-
Blurred vision, brain inflammation	1	1/394 (0%)	62 (62-62)	0	0/308 (0%)	-
- Depressed consciousness, brain tuberculomas	1	1/394 (0%)	67 (67-67)	1	0/308 (0%)	-
- Paradoxical reaction	1	1/394 (0%)	29 (29-29)	0	0/308 (0%)	-
- Worsening headache and tuberculoma	1	1/394 (0%)	46 (46-46)	0	0/308 (0%)	-
- Critically ill	1	1/394 (0%)	35 (35-35)	0	0/308 (0%)	-
- Worsening CSF inflammation, brain inflammation	1	1/394 (0%)	61 (61-61)	0	0/308 (0%)	-
- Pancreatitis, severe intestinal paralysis	1	1/394 (0%)	41 (41-41)	0	0/308 (0%)	-
- Vision decreased	1	1/394 (0%)	57 (57-57)	0	0/308 (0%)	-
- Itchy rash	1	1/394 (0%)	103 (103-103)	0	0/308 (0%)	-
- Urticaria	1	1/394 (0%)	35 (35-35)	0	0/308 (0%)	-

		Dexamethasone (N=394)			Placebo (N=308)	
**Reason**	**n episode**	**n participants**	**Days from randomization until use of open-label corticosteroids**	**n episode**	**n participants**	**Days from randomization until use of open-label corticosteroids**
- Headache and focal neurological sign	1	1/394 (0%)	23 (23-23)	0	0/308 (0%)	-
- Suspected increased intracranial pressure	1	1/394 (0%)	63 (63-63)	0	0/308 (0%)	-
- Pneumonia	1	1/394 (0%)	13 (13-13)	0	0/308 (0%)	-
- Rash	1	1/394 (0%)	31 (31-31)	0	0/308 (0%)	-
- Asthma	1	1/394 (0%)	32 (32-32)	0	0/308 (0%)	-
- Paralysis	1	1/394 (0%)	138 (138-138)	0	0/308 (0%)	-
- Stroke	1	1/394 (0%)	69 (69-69)	0	0/308 (0%)	-
- Hyponatraemia	1	1/394 (0%)	30 (30-30)	0	0/308 (0%)	-
- Headache and nausea	1	1/394 (0%)	44 (44-44)	0	0/308 (0%)	-
- Lethargy	1	1/394 (0%)	5 (5-5)	0	0/308 (0%)	-
- Drug allergy	1	1/394 (0%)	70 (70-70)	0	0/308 (0%)	-
- COVID-19	1	1/394 (0%)	23 (23-23)	0	0/308 (0%)	-
- Raised intracranial pressure	0	0/394 (0%)	-	1	1/308 (0%)	23 (23-23)
- New lesion on MRI brain	0	0/394 (0%)	-	1	1/308 (0%)	90 (90-90)
- Worsening headache	0	0/394 (0%)	-	1	1/308 (0%)	90 (90-90)
- Paradoxical reaction with MRI changes consistent with myelitis	0	0/394 (0%)	-	1	1/308 (0%)	41 (41-41)
- Neurological deficits and brain lesions	0	0/394 (0%)	-	1	1/308 (0%)	35 (35-35)
- Blurred vision, optochiasmatic arachnoiditis	0	0/394 (0%)	-	1	1/308 (0%)	61 (61-61)

		Dexamethasone (N=394)			Placebo (N=308)	
**Reason**	**n episode**	**n participants**	**Days from randomization until use of open-label corticosteroids**	**n episode**	**n participants**	**Days from randomization until use of open-label corticosteroids**
- Depressed consciousness, cerebral infarction, brain inflammation, worsening CSF	0	0/394 (0%)	-	1	1/308 (0%)	23 (23-23)
Blurred vision, worsening CSF, brain inflammation	0	0/394 (0%)	-	1	1/308 (0%)	64 (64-64)
- Headache, fever, cerebral oedema and worsening CSF inflammation	0	0/394 (0%)	-	1	1/308 (0%)	32 (32-32)
- Depressed consciousness and hydrocephalus	0	0/394 (0%)	-	1	1/308 (0%)	28 (28-28)
- Depressed level of consciousness and cerebral herniation	0	0/394 (0%)	-	1	1/308 (0%)	37 (37-37)
- Haemophagocytosis	0	0/394 (0%)	-	1	1/308 (0%)	80 (80-80)
- Cholecystitis	0	0/394 (0%)	-	1	1/308 (0%)	16 (16-16)
- Cerebral oedema	0	0/394 (0%)	-	1	1/308 (0%)	23 (23-23)
- Coma	0	0/394 (0%)	-	1	1/308 (0%)	15 (15-15)
- Gout	0	0/394 (0%)	-	1	1/308 (0%)	13 (13-13)
- Respiratory failure, COVID-19	0	0/394 (0%)	-	1	1/308 (0%)	122 (122-122)
- Tuberculoma, cerebral oedema, seizure, hemiplegia	0	0/394 (0%)	-	1	1/308 (0%)	25 (25-25)
- Subdural hematoma	0	0/394 (0%)	-	1	1/308 (0%)	56 (56-56)

Summary statistic = the mean, median (1st and 3rd quartile) value for days to events and frequency (%) for number of episode and number of participants with that particular reason. CSF=cerebrospinal fluid. ITT=intention-to-treat. MRI=magnetic resonance imaging. TB=tuberculosis.

Table S28. Neurological disability at 12 months from randomization, by treatment arms in the ITT population

**CC/CT-genotype**		**Dexamethasone (N=305)**		**Placebo (N=308)**	
**Characteristic**	**n**	**Summary statistic**	**n**	**Summary statistic**	**OR (95% CI)**
Neurological disability at 12 months	304		303		
- No		215/304 (70.7%)		219/303 (72.3%)	1.13 (0.76, 1.68)
- Yes		89/304 (29.3%)		84/303 (27.7%)	
**CC-genotype**					
Neurological disability at 12 months	145		141		
- No		105/145 (72.4%)		98/141 (69.5%)	0.87 (0.50, 1.51)
- Yes		40/145 (27.6%)		43/141 (30.5%)	

N is number of all participants, n is number of participants with non-missing value. Summary statistic is absolute count (%) for categorical variable(s). CI=confidence interval. ITT=intention-to-treat. OR=odds ratio.

Table S29. All modified Rankin scores at 12 months from randomization in the ITT population

**CC/CT-genotype**		**Dexamethasone (N=305)**		**Placebo (N=308)**	**Comparison**
Characteristic	N	Summary statistic	n	Summary statistic	Cumulative Odds ratio (95% CI)
12-month Modified Rankin score	304		303		0.81 (0.60, 1.10)
- 0		130/304 (42.8%)		108/303 (35.6%)	
- 1		76/304 (25.0%)		90/303 (29.7%)	
- 2		9/304 (3.0%)		21/303 (6.9%)	
- 3		13/304 (4.3%)		10/303 (3.3%)	
- 4		7/304 (2.3%)		5/303 (1.7%)	
- 5		3/304 (1.0%)		3/303 (1.0%)	
- 6		66/304 (21.7%)		66/303 (21.8%)	
CC-genotype	145		141		0.63 (0.40, 0.97)
- 0		65/145 (44.8%)		49/141 (34.8%)	
- 1		35/145 (24.1%)		39/141 (27.7%)	
- 2		5/145 (3.4%)		10/141 (7.1%)	
- 3		6/145 (4.1%)		4/141 (2.8%)	
- 4		5/145 (3.4%)		3/141 (2.1%)	
- 5		3/145 (2.1%)		2/141 (1.4%)	
- 6		26/145 (17.9%)		34/141 (24.1%)	

N is number of all participants, n is number of participants with non-missing value. CI=confidence interval. ITT=intention-to-treat.

Table S30. Overall incidence of death over the first 12 months after randomization in the per-protocol population

Genotype	Endpoint	Dexamethasone (n=297)	Placebo (n=288)	Comparison	
		events/n (risk [%])	events/n (risk [%])	HR (95%CI); p-value	RMTL difference (95%CI); p-value
CC & CT	3 months	41/297 (13.8)	37/288 (12.9)		
	6 months	54/297 (18.2)	47/288 (16.3)		
	9 months	56/297 (18.9)	55/288 (19.1)		
	12 months	62/297 (20.9)	58/288 (20.1)	1.10 (0.77, 1.58); p=0.60	0.13 (-0.47, 0.72); p=0.68
CC	3 months	16/140 (11.4)	20/132 (15.2)		
	6 months	20/140 (14.3)	25/132 (18.9)		
	9 months	22/140 (15.7)	28/132 (21.2)		
	12 months	24/140 (17.1)	29/132 (22.0)	0.74 (0.43, 1.28); p=0.28	-0.46 (-1.35, 0.42); p=0.31

CI=confidence interval. HR=hazard ratio. RMTL=restricted mean time lost.

Table S31. Deaths in prespecified subgroups in the per-protocol population

Subgroup	Dexamethasone (n=297)	Placebo (n=288)	Comparison	Test for heterogeneity
	events/n (risk [%])	events/n (risk [%])	HR (95%CI); p-value	p-value
All participants	62/297 (20.9)	58/288 (20.1)	1.04 (0.73, 1.49); p=0.83	
LTA4H genotype				0.12
- CC	24/140 (17.1)	29/132 (22.0)	0.76 (0.44, 1.31); p=0.32	
- CT	38/157 (24.2)	29/156 (18.6)	1.35 (0.83, 2.18); p=0.23	
Modified MRC grade				0.57
- Grade I	10/132 (7.6)	6/128 (4.7)	1.65 (0.60, 4.53); p=0.33	
- Grade II	39/146 (26.7)	39/139 (28.1)	0.94 (0.60, 1.46); p=0.77	
- Grade III	13/19 (68.4)	13/21 (61.9)	1.16 (0.54, 2.50); p=0.71	
TBM diagnosis				0.79
- Definite	20/123 (16.3)	22/128 (17.2)	0.93 (0.51, 1.71); p=0.82	
- Probable	30/106 (28.3)	28/101 (27.7)	1.02 (0.61, 1.71); p=0.93	
- Possible	12/68 (17.7)	8/59 (13.6)	1.35 (0.55, 3.31); p=0.51	

CI=confidence interval. HR=hazard ratio. LTA4H=leukotriene A4 hydrolase. MRC=Medical Research Council. TBM=tuberculous meningitis.

Table S32. Restricted mean time lost (RMTL) for deaths in the per-protocol population

Subgroup	Dexamethasone (n=297)	Placebo (n=288)	Comparison	Test for heterogeneity
	RMTL (SE, months)	RMTL (SE, months)	RMTL difference (95%CI); p-value	p-value
All participants	1.92 (0.23)	1.84 (0.23)	0.08 (-0.56, 0.73); p=0.80	
LTA4H genotype				0.11
- CC	1.58 (0.31)	2.05 (0.35)	-0.47 (-1.40, 0.46); p=0.32	
- CT	2.24 (0.34)	1.66 (0.30)	0.57 (-0.32, 1.46); p=0.21	
Modified MRC grade				0.67
- Grade I	0.69 (0.22)	0.37 (0.16)	0.32 (-0.22, 0.86); p=0.25	
- Grade II	2.38 (0.35)	2.54 (0.37)	-0.16 (-1.17, 0.85); p=0.76	
- Grade III	7.00 (1.14)	6.17 (1.12)	0.83 (-2.47, 4.12); p=0.62	
TBM diagnosis				0.78
- Definite	1.40 (0.31)	1.52 (0.32)	-0.12 (-0.99, 0.76); p=0.80	
- Probable	2.65 (0.44)	2.57 (0.44)	0.08 (-1.15, 1.31); p=0.90	
- Possible	1.74 (0.47)	1.29 (0.43)	0.45 (-0.83, 1.73); p=0.49	

CI=confidence interval. LTA4H=leukotriene A4 hydrolase. MRC=Medical Research Council. SE=standard error. TBM=tuberculous meningitis.

Table S33. Overall risk of first neurological event over the first 12 months after randomization in the per-protocol population

Genotype	Endpoint	Dexamethasone (n=297)	Placebo (n=288)	Comparison	
		events/n (risk [%])	events/n (risk [%])	HR (95%CI); p-value	RMTL difference (95%CI); p-value
CC & CT	3 months	66/297 (22.2)	66/288 (22.9)		
	6 months	80/297 (26.9)	74/288 (25.7)		
	9 months	88/297 (29.6)	77/288 (26.7)		
	12 months	90/297 (30.3)	81/288 (28.1)	1.06 (0.78 1.43); p=0.71	0.11 (-0.64, 0.86); p=0.78
CC	3 months	7/140 (5.0)	4/132 (3.0)		
	6 months	8/140 (5.7)	5/132 (3.8)		
	9 months	13/140 (9.3)	10/132 (7.6)		
	12 months	43/140 (30.7)	42/132 (31.1)	0.86 (0.56, 1.32); p=0.49	-0.32 (-1.45, 0.80); p=0.57

CI=confidence interval. HR=hazard ratio. RMTL=restricted mean time lost.

Table S34. New neurological events in prespecified sub-groups in the per-protocol population

Subgroup	Dexamethasone (n=297)	Placebo (n=288)	Comparison	Test for heterogeneity
	events/n (risk [%])	events/n (risk [%])	HR (95%CI); p-value	p-value
All participants	90/297 (30.3)	81/288 (28.1)	1.05 (0.78, 1.42); p=0.74	
LTA4H genotype				0.33
- CC	43/140 (30.7)	43/132 (32.6)	0.91 (0.59, 1.38); p=0.65	
- CT	47/157 (29.9)	38/156 (24.4)	1.22 (0.80, 1.87); p=0.36	
Modified MRC grade				0.42
- Grade I	18/132 (13.6)	23/128 (18.0)	0.75 (0.40, 1.39); p=0.36	
- Grade II	61/146 (41.8)	49/139 (35.3)	1.15 (0.79, 1.67); p=0.47	
- Grade III	11/19 (57.9)	9/21 (42.9)	1.34 (0.55, 3.24); p=0.52	
TBM diagnosis				0.18
- Definite	41/123 (33.3)	47/128 (36.7)	0.86 (0.56, 1.30); p=0.46	
- Probable	35/106 (33.0)	21/101 (20.8)	1.61 (0.94, 2.77); p=0.08	
- Possible	14/68 (20.6)	13/59 (22.0)	0.94 (0.44, 2.00); p=0.87	

CI=confidence interval. HR=hazard ratio. LTA4H=leukotriene A4 hydrolase. MRC=Medical Research Council. TBM=tuberculous meningitis.

Table S35. Restricted mean time lost (RMTL) for new neurological events in the per-protocol population

Subgroup	Dexamethasone (n=297)	Placebo (n=288)	Comparison	Test for heterogeneity
	RMTL (SE, months)	RMTL (SE, months)	RMTL difference (95%CI); p-value	p-value
All participants	2.99 (0.27)	2.89 (0.28)	0.10 (-0.68, 0.87); p=0.81	
LTA4H genotype				0.35
- CC	2.98 (0.40)	3.28 (0.43)	-0.31 (-1.47, 0.85); p=0.60	
- CT	3.00 (0.38)	2.56 (0.37)	0.44 (-0.60, 1.49); p=0.41	
Modified MRC grade				0.52
- Grade I	1.41 (0.31)	1.76 (0.35)	-0.35 (-1.28, 0.57); p=0.46	
- Grade II	4.03 (0.42)	3.63 (0.43)	0.40 (-0.78, 1.59); p=0.51	
- Grade III	5.97 (1.22)	4.93 (1.24)	1.04 (-2.56, 4.63); p=0.57	
TBM diagnosis				0.22
- Definite	3.32 (0.44)	3.81 (0.46)	-0.50 (-1.76, 0.76); p=0.44	
- Probable	3.09 (0.45)	2.06 (0.42)	1.03 (-0.20, 2.25); p=0.10	
- Possible	2.25 (0.54)	2.32 (0.58)	-0.07 (-1.65, 1.50); p=0.93	

CI=confidence interval. LTA4H=leukotriene A4 hydrolase. MRC=Medical Research Council. SE=standard error. TBM=tuberculous meningitis.

Table S36. Overall risk of first use of open-label corticosteroid over the first 12 months after randomization in the per-protocol population

Genotype	Endpoint	Dexamethasone (n=297)	Placebo (n=288)	Comparison	
		events/n (risk [%])	events/n (risk [%])	HR (95%CI); p-value	RMTL difference (95%CI); p-value
CC & CT	3 months	43/297 (14.5)	37/288 (12.9)		
	6 months	49/297 (16.5)	41/288 (14.2)		
	9 months	50/297 (16.9)	43/288 (14.9)		
	12 months	52/297 (17.5)	44/288 (15.3)	1.13 (0.75, 1.69); p=0.56	0.22 (-0.40, 0.84); p=0.49
CC	3 months	20/140 (14.3)	19/132 (14.4)		
	6 months	23/140 (16.4)	22/132 (16.7)		
	9 months	23/140 (16.4)	23/132 (17.4)		
	12 months	24/140 (17.1)	24/132 (18.2)	0.88 (0.50, 1.54); p=0.65	-0.09 (-1.03, 0.85); p=0.85

CI=confidence interval. HR=hazard ratio. RMTL=restricted mean time lost.

Table S37. First use of open label corticosteroids in prespecified sub-groups in the per protocol population

Subgroup	Dexamethasone (n=297)	Placebo (n=288)	Comparison	Test for heterogeneity
	events/n (risk [%])	events/n (risk [%])	HR (95%CI); p-value	p-value
All participants	52/297 (17.5)	44/288 (15.3)	1.14 (0.76, 1.71); p=0.52	
LTA4H genotype				0.28
- CC	24/140 (17.1)	24/132 (18.2)	0.91 (0.52, 1.60); p=0.74	
- CT	28/157 (17.8)	20/156 (12.8)	1.42 (0.80, 2.52); p=0.23	
Modified MRC grade				0.32
- Grade I	19/132 (14.4)	14/128 (10.9)	1.36 (0.68, 2.70); p=0.39	
- Grade II	27/146 (18.5)	27/139 (19.4)	0.91 (0.53, 1.55); p=0.73	
- Grade III	6/19 (31.6)	3/21 (14.3)	2.39 (0.60, 9.57); p=0.22	
TBM diagnosis				0.19
- Definite	30/123 (24.4)	27/128 (21.1)	1.08 (0.64, 1.82); p=0.76	
- Probable	17/106 (16.1)	9/101 (8.9)	1.88 (0.84, 4.23); p=0.12	
- Possible	5/68 (7.4)	8/59 (13.6)	0.54 (0.18, 1.65); p=0.28	

CI=confidence interval. HR=hazard ratio. LTA4H=leukotriene A4 hydrolase. MRC=Medical Research Council. TBM=tuberculous meningitis.

Table S38. New use of open label corticosteroids in prespecified sub-groups, with death as competing risk in the per-protocol population

Subgroup	Dexamethasone (n=297)	Placebo (n=288)	Comparison	Test for heterogeneity
	events/n (risk [%])	events/n (risk [%])	RMTL difference (95%CI); p-value	p-value
All participants	52/297 (17.5)	44/288 (15.3)	0.21 (-0.40, 0.83); p=0.50	
LTA4H genotype				0.39
- CC	24/140 (17.1)	24/132 (18.2)	-0.08 (-1.01, 0.85); p=0.87	
- CT	28/157 (17.8)	20/156 (12.8)	0.46 (-0.37, 1.29); p=0.27	
Modified MRC grade				0.42
- Grade I	19/132 (14.4)	14/128 (10.9)	0.37 (-0.44, 1.18); p=0.37	
- Grade II	27/146 (18.5)	27/139 (19.4)	-0.14 (-1.09, 0.82); p=0.78	
- Grade III	6/19 (31.6)	3/21 (14.3)	1.76 (-1.15, 4.67); p=0.24	
TBM diagnosis				0.35
- Definite	30/123 (24.4)	27/128 (21.1)	0.27 (-0.82, 1.35); p=0.63	
- Probable	17/106 (16.4)	9/101 (8.9)	0.63 (-0.31, 1.57); p=0.19	
- Possible	5/68 (7.4)	8/59 (13.6)	-0.38 (-1.38, 0.63); p=0.46	

CI=confidence interval. LTA4H=leukotriene A4 hydrolase. MRC=Medical Research Council. RMTL=restricted mean time lost. TBM=tuberculous meningitis.

Table S39. Overall risk of first use of open-label corticosteroid over the first 12 months after randomization in the per-protocol population

Genotype	Endpoint	Dexamethasone (n=297)	Placebo (n=288)	Comparison	
		events/n (risk [%])	events/n (risk [%])	HR (95%CI); p-value	RMTL difference (95%CI); p-value
CC & CT	3 months	25/297 (8.4)	17/288 (5.9)		
	6 months	26/297 (8.8)	19/288 (6.6)		
	9 months	26/297 (8.8)	21/288 (7.3)		
	12 months	27/297 (9.1)	22/288 (7.6)	1.21 (0.68, 2.15); p=0.51	0.22 (-0.25, 0.68); p=0.36
CC	3 months	12/140 (8.6)	8/132 (6.1)		
	6 months	12/140 (8.6)	10/132 (7.6)		
	9 months	12/140 (8.6)	11/132 (8.3)		
	12 months	12/140 (8.6)	12/132 (9.1)	0.90 (0.40, 2.01); p=0.80	0.11 (-0.58, 0.80); p=0.76

CI=confidence interval. HR=hazard ratio. RMTL=restricted mean time lost.

Table S40. New use of open label corticosteroids in prespecified subgroups in the per-protocol population

Subgroup	Dexamethasone (n=297)	Placebo (n=288)	Comparison	Test for heterogeneity
	events/n (risk [%])	events/n (risk [%])	HR (95%CI); p-value	p-value
All participants	27/297 (9.1)	22/288 (7.6)	1.16 (0.66, 2.04); p=0.60	
LTA4H genotype				0.41
- CC	12/140 (8.6)	12/132 (9.1)	0.92 (0.41, 2.04); p=0.83	
- CT	15/157 (9.6)	10/156 (6.4)	1.46 (0.65, 3.24); p=0.36	
Modified MRC grade				0.26
- Grade I	10/132 (7.6)	10/128 (7.8)	0.96 (0.40, 2.30); p=0.92	
- Grade II	13/146 (8.9)	11/139 (7.9)	1.07 (0.48, 2.40); p=0.86	
- Grade III	4/19 (21.1)	1/21 (4.8)	5.30 (0.59, 47.5); p=0.14	
TBM diagnosis				0.07
- Definite	15/123 (12.2)	11/128 (8.6)	1.30 (0.60, 2.82); p=0.51	
- Probable	11/106 (10.4)	6/101 (5.9)	1.82 (0.67, 4.92); p=0.24	
- Possible	1/68 (1.5)	5/59 (8.5)	0.16 (0.02, 1.41); p=0.10	

CI=confidence interval. HR=hazard ratio. LTA4H=leukotriene A4 hydrolase. MRC=Medical Research Council. TBM=tuberculous meningitis.

Table S41. Restricted mean time lost (RMTL) for new use of open label corticosteroids with the primary endpoint as a competing risk in the per-protocol population

Subgroup	Dexamethasone (n=297)	Placebo (n=288)	Comparison	Test for heterogeneity
	RMTL (SE, months)	RMTL (SE, months)	RMTL difference (95%CI); p-value	p-value
All participants	0.94 (0.18)	0.73 (0.16)	0.21 (-0.25, 0.68); p=0.36	
LTA4H genotype				0.68
- CC	0.92 (0.25)	0.81 (0.24)	-0.11 (-0.58, 0.79); p=0.76	
- CT	0.96 (0.24)	0.66 (0.21)	0.31 (-0.32, 0.93); p=0.34	
Modified MRC grade				0.38
- Grade I	0.73 (0.23)	0.70 (0.22)	0.04 (-0.60, 0.67); p=0.91	
- Grade II	0.96 (0.25)	0.79 (0.24)	0.17 (-0.51, 0.86); p=0.62	
- Grade III	2.26 (1.01)	0.52 (0.50)	1.74 (-0.58, 4.07); p=0.14	
TBM diagnosis				0.16
- Definite	1.27 (0.31)	0.87 (0.26)	0.39 (-0.40, 1.19); p=0.33	
- Probable	1.06 (0.31)	0.62 (0.25)	0.44 (-0.35, 1.23); p=0.28	
- Possible	0.17 (0.17)	0.59 (0.28)	-0.42 (-1.07, 0.23); p=0.21	

CI=confidence interval. LTA4H=leukotriene A4 hydrolase. MRC=Medical Research Council. SE=standard error. TBM=tuberculous meningitis.

Table S42. Death or disability in the per-protocol population

A) CC/CT

**CC/CT-genotype**		**Dexamethasone (N=297)**		**Placebo (N=288)**	**Comparison**
Characteristic	n	Summary statistic	n	Summary statistic	OR (95% CI)
Neurological disability at 12 months	297		287		
- No		214/297 (72.1%)		211/287 (73.5%)	1.12 (0.75, 1.69)
- Yes		83/297 (27.9%)		76/287 (26.5%)	
**CC-genotype**	140		132		
- No		104/140 (74.3%)		94/132 (71.2%)	0.84 (0.47, 1.49)
- Yes		36/140 (25.7%)		38/132 (28.8%)	

N is number of all participants, n is number of participants with non-missing value. CI=confidence interval. OR=odds ratio.

Table S43. All modified Rankin scores at 12 months from randomization in the per-protocol population

		Dexamethasone (N=297)		Placebo (N=288)	Comparison
**Characteristic**	**N**	**Summary statistic**	**N**	**Summary statistic**	**Odds ratio (95% CI)**
**CC/CT genotype**					
Modified Rankin score	297		287		0.80 (0.59, 1.09)
- 0		129/297 (43.4%)		103/287 (35.9%)	
- 1		76/297 (25.6%)		88/287 (30.7%)	
- 2		9/297 (3.0%)		20/287 (7.0%)	
- 3		13/297 (4.4%)		10/287 (3.5%)	
- 4		7/297 (2.4%)		5/287 (1.7%)	
- 5		1/297 (0.3%)		3/287 (1.0%)	
- 6		62/297 (20.9%)		58/287 (20.2%)	
**CC-genotype**					
Modified Rankin score	140		132		0.61 (0.39, 0.96)
- 0		64/140 (45.7%)		47/132 (35.6%)	
- 1		35/140 (25.0%)		37/132 (28.0%)	
- 2		5/140 (3.6%)		10/132 (7.6%)	
- 3		6/140 (4.3%)		4/132 (3.0%)	
- 4		5/140 (3.6%)		3/132 (2.3%)	
- 5		1/140 (0.7%)		2/132 (1.5%)	
- 6		24/140 (17.1%)		29/132 (22.0%)	

CI=confidence interval.

Table S44. Serious adverse events, and Grade 3 or 4 adverse events, by treatment arm in the CC-genotype

	Dexamethasone (N=146)		Placebo (N=145)	
**Type of adverse event**	**n episode**	**n participants**	**n episode**	**n participants**
CC genotype: Any serious adverse event	154	73/146 (50%)	198	81/145 (56%)
Grade 3 adverse events	125	64/146 (44%)	157	65/145 (45%)
Grade 4 adverse events	40	30/146 (21%)	54	41/145 (28%)
Fall in GCS ≥ 2 points, for ≥ 48hrs	16	16/146 (11%)	30	27/145 (19%)
Urinary tract infection	13	10/146 (7%)	15	11/145 (8%)
Hyponatraemia requiring intervention or medically important	8	8/146 (5%)	18	18/145 (12%)
New focal neurological sign lasting >24 hours	16	14/146 (10%)	5	5/145 (3%)
Elevation of blood transaminases ≥ 5 times the upper limit of normal or a rise in serum bilirubin >2.0mg/dL (>34 mol/L) without symptoms	8	8/146 (5%)	13	10/145 (7%)
Lung infection	8	8/146 (5%)	13	9/145 (6%)
Hypokalaemia	10	9/146 (6%)	10	8/145 (6%)
Upper gastrointestinal haemorrhage	5	4/146 (3%)	5	5/145 (3%)
Respiratory failure	4	4/146 (3%)	6	6/145 (4%)
Depressed level of consciousness	6	6/146 (4%)	2	2/145 (1%)
Sepsis	1	1/146 (1%)	7	7/145 (5%)
Hydrocephalus	3	3/146 (2%)	4	4/145 (3%)
Elevation of blood transaminase concentrations ≥ 3 times the upper limit of normal with symptoms and signs of hepatitis (vomiting, abdominal pain, jaundice)	1	1/146 (1%)	6	6/145 (4%)
Hypotension	2	2/146 (1%)	4	2/145 (1%)
Nervous system disorders - Other, worsening tuberculous meningitis	3	3/146 (2%)	2	2/145 (1%)
Skin and subcutaneous tissue disorders - Other, TB drug rash	1	1/146 (1%)	4	4/145 (3%)
Headache	2	2/146 (1%)	2	2/145 (1%)
Nervous system disorders - Other, focal neurological sign	2	2/146 (1%)	1	1/145 (1%)

	Dexamethasone (N=146)		Placebo (N=145)	
**Type of adverse event**	**n episode**	**n participants**	**n episode**	**n participants**
Seizure	2	2/146 (1%)	1	1/145 (1%)
Vomiting	2	2/146 (1%)	1	1/145 (1%)
Infections and infestations - Other, COVID-19	2	2/146 (1%)	1	1/145 (1%)
Skin ulceration	1	1/146 (1%)	2	2/145 (1%)
Anaemia	0	0/146 (0%)	3	2/145 (1%)
Infections and infestations - Other, drug resistant tuberculosis				
Oedema cerebral	2	2/146 (1%)	0	0/145 (0%)
Cough	2	2/146 (1%)	0	0/145 (0%)
Death NOS	2	2/146 (1%)	0	0/145 (0%)
Investigations - Other, pancytopaenia	1	1/146 (1%)	1	1/145 (1%)
Stroke	1	1/146 (1%)	1	1/145 (1%)
Nervous system disorders - Other, tuberculoma	1	1/146 (1%)	1	1/145 (1%)
Nervous system disorders - Other, depressed consciousness and hydrocephalus	1	1/146 (1%)	1	1/145 (1%)
Endocrine disorders - Other, polyuria	1	1/146 (1%)	1	1/145 (1%)
Dizziness	1	1/146 (1%)	1	1/145 (1%)
Thromboembolic event	1	1/146 (1%)	1	1/145 (1%)
Blurred vision	1	1/146 (1%)	2	2/145 (1%)
Acute kidney injury	1	1/146 (1%)	1	1/145 (1%)
Metabolism and nutrition disorders - Other, malnutrition	1	1/146 (1%)	1	1/145 (1%)
Lymph gland infection	0	0/146 (0%)	2	2/145 (1%)
Adrenal insufficiency	0	0/146 (0%)	2	2/145 (1%)
Blood bilirubin increased	0	0/146 (0%)	2	2/145 (1%)
Eczema	1	1/146 (1%)	0	0/145 (0%)
Infections and infestations - Other, dengue fever	1	1/146 (1%)	0	0/145 (0%)
Supraventricular tachycardia	1	1/146 (1%)	0	0/145 (0%)
Alanine aminotransferase increased	1	1/146 (1%)	0	0/145 (0%)

	Dexamethasone (N=146)		Placebo (N=145)	
Type of adverse event	n episode	n participants	n episode	n participants
Gastrointestinal disorders - Other, greater than 5mls of fresh or changed blood aspirated from nasogastric tube	1	1/146 (1%)	0	0/145 (0%)
Paraesthesia	1	1/146 (1%)	0	0/145 (0%)
Hypomagnesaemia	1	1/146 (1%)	0	0/145 (0%)
Nervous system disorders - Other, tuberculoma and hydrocephalus	1	1/146 (1%)	0	0/145 (0%)
Gastritis	1	1/146 (1%)	0	0/145 (0%)
Gastrointestinal disorders - Other, nausea and vomiting	1	1/146 (1%)	0	0/145 (0%)
Nervous system disorders - Other, headache and hypertension	1	1/146 (1%)	0	0/145 (0%)
Hepatobiliary disorders - Other, liver tumour	1	1/146 (1%)	0	0/145 (0%)
Infections and infestations - Other, superadded infection	1	1/146 (1%)	0	0/145 (0%)
Colitis	1	1/146 (1%)	0	0/145 (0%)
Nervous system disorders - Other, headache and vomiting	1	1/146 (1%)	0	0/145 (0%)
Nervous system disorders - Other, spinal tuberculosis	1	1/146 (1%)	0	0/145 (0%)
Oesophageal fistula	1	1/146 (1%)	0	0/145 (0%)
Biliary tract infection	1	1/146 (1%)	0	0/145 (0%)
Nervous system disorders - Other, blurred vision	0	0/146 (0%)	1	1/145 (1%)
Chest wall pain	0	0/146 (0%)	1	1/145 (1%)
Nervous system disorders - Other, dizziness and vomiting	0	0/146 (0%)	1	1/145 (1%)
General disorders and administration site conditions - Other, fever and arm pain	0	0/146 (0%)	1	1/145 (1%)
Blood antidiuretic hormone abnormal	0	0/146 (0%)	1	1/145 (1%)
Myelitis	0	0/146 (0%)	1	1/145 (1%)
Nervous system disorders - Other, cerebellar signs and radiculitis	0	0/146 (0%)	1	1/145 (1%)

	Dexamethasone (N=146)		Placebo (N=145)	
**Type of adverse event**	**n episode**	**n participants**	**n episode**	**n participants**
Cardiac disorders - Other, conduction disorder and hypokalaemia	0	0/146 (0%)	1	1/145 (1%)
Oedema limbs	0	0/146 (0%)	1	1/145 (1%)
Cataract	0	0/146 (0%)	1	1/145 (1%)
Eye disorders - Other, subconjunctival haemorrhage	0	0/146 (0%)	1	1/145 (1%)
Shingles	0	0/146 (0%)	1	1/145 (1%)
Anaphylaxis	0	0/146 (0%)	1	1/145 (1%)
Viraemia	0	0/146 (0%)	1	1/145 (1%)
Metabolism and nutrition disorders - Other, anorexia and vomiting	0	0/146 (0%)	1	1/145 (1%)
Ileus	0	0/146 (0%)	1	1/145 (1%)
Skin infection	0	0/146 (0%)	1	1/145 (1%)
Cardiac arrest	0	0/146 (0%)	1	1/145 (1%)
Blood and lymphatic system disorders - Other, haemophagocytic disorder	0	0/146 (0%)	1	1/145 (1%)
Gastrointestinal disorders - Other, dysphagia and anorexia	0	0/146 (0%)	1	1/145 (1%)
Cholecystitis	0	0/146 (0%)	1	1/145 (1%)
Cerebrospinal fluid leakage	0	0/146 (0%)	1	1/145 (1%)
Eye infection	0	0/146 (0%)	1	1/145 (1%)
Encephalomyelitis infection	0	0/146 (0%)	1	1/145 (1%)
Nervous system disorders - Other, focal neurological sign and cerebral oedema	0	0/146 (0%)	1	1/145 (1%)
Hyperglycaemia	0	0/146 (0%)	1	1/145 (1%)

For any serious adverse event in CC genotype participants, at least one event occurred in 73/146 participants receiving dexamethasone, and at least one event occurred in 81/145 participants receiving placebo. p-value for number of participants with serious adverse event(s) = 0.32. p-value for total number of serious adverse events per participant = 0.99. Events summarized according to the System Organ Class of the Medical Dictionary for Regulatory Activities (MedDRA) hierarchy. GCS=Glasgow coma scale. TB=tuberculosis. NOS=not otherwise specified. SAEs=serious adverse events.

Table S45. Serious adverse events, and Grade 3 or 4 adverse events, by treatment arm in the CT-genotype

	Dexamethasone (N=159)		Placebo (N=163)	
**Type of adverse event**	**n episode**	**n participants**	**n episode**	**n participants**
CT genotype: Any serious adverse event	171	88/159 (55%)	173	79/163 (48%)
Grade 3 adverse events	136	70/159 (44%)	145	65/163 (40%)
Grade 4 adverse events	47	37/159 (23%)	46	34/163 (21%)
Fall in GCS ≥ 2 points, for ≥ 48hrs	26	26/159 (16%)	17	17/163 (10%)
Hyponatraemia requiring intervention or medically important	11	11/159 (7%)	17	16/163 (10%)
Lung infection	10	10/159 (6%)	18	16/163 (10%)
Urinary tract infection	12	10/159 (6%)	15	12/163 (7%)
New focal neurological sign lasting >24 hours	11	8/159 (5%)	9	8/163 (5%)
Elevation of blood transaminases ≥ 5 times the upper limit of normal or a rise in serum bilirubin >2.0mg/dL (>34 mol/L) without symptoms	10	10/159 (6%)	5	5/163 (3%)
Hypokalaemia	5	5/159 (3%)	8	7/163 (4%)
Sepsis	6	5/159 (3%)	4	4/163 (2%)
Respiratory failure	5	5/159 (3%)	5	5/163 (3%)
Anaemia	1	1/159 (1%)	8	8/163 (5%)
Skin and subcutaneous tissue disorders - Other, rash of unknown aetiology	4	4/159 (3%)	3	3/163 (2%)
Upper gastrointestinal haemorrhage	3	3/159 (2%)	3	3/163 (2%)
Death NOS	2	2/159 (1%)	3	3/163 (2%)
Skin and subcutaneous tissue disorders - Other, TB drug rash	4	4/159 (3%)	0	0/163 (0%)
Hydrocephalus	3	3/159 (2%)	1	1/163 (1%)
Infections and infestations - Other, COVID-19	3	3/159 (2%)	1	1/163 (1%)
Elevation of blood transaminase concentrations ≥ 3 times the upper limit of normal with symptoms and signs of hepatitis (vomiting, abdominal pain, jaundice)	0	0/159 (0%)	4	4/163 (2%)
Nervous system disorders - Other, tuberculoma	3	3/159 (2%)	0	0/163 (0%)

Headache	3	3/159 (2%)	0	0/163 (0%)
Blurred vision	2	2/159 (1%)	1	1/163 (1%)
Vomiting	2	2/159 (1%)	1	1/163 (1%)
Depressed level of consciousness	2	2/159 (1%)	1	1/163 (1%)
Nervous system disorders - Other, worsening tuberculous meningitis	1	1/159 (1%)	2	2/163 (1%)
Creatinine increased	1	1/159 (1%)	2	2/163 (1%)
Hypertension	1	1/159 (1%)	2	2/163 (1%)
Adrenal insufficiency	0	0/159 (0%)	3	3/163 (2%)
Intracranial haemorrhage	0	0/159 (0%)	3	3/163 (2%)
Acute kidney injury	0	0/159 (0%)	3	2/163 (1%)
Elevated liver enzymes	2	2/159 (1%)	0	0/163 (0%)
General disorders and administration site conditions - Other, withdraw from hospital treatment	2	2/159 (1%)	0	0/163 (0%)
Stroke	2	2/159 (1%)	0	0/163 (0%)
Nervous system disorders - Other, hydrocephalus and tuberculoma	2	2/159 (1%)	0	0/163 (0%)
Cerebrospinal fluid leakage	1	1/159 (1%)	1	1/163 (1%)
Nervous system disorders - Other, focal neurological sign	1	1/159 (1%)	1	1/163 (1%)
Nervous system disorders - Other, subdural haematoma	1	1/159 (1%)	1	1/163 (1%)
Infections and infestations - Other, thigh abscess	1	1/159 (1%)	1	1/163 (1%)
Oedema cerebral	1	1/159 (1%)	1	1/163 (1%)
Pleural effusion	0	0/159 (0%)	3	3/163 (2%)
Hyperuricaemia	0	0/159 (0%)	2	2/163 (1%)
Nervous system disorders - Other, headache and vomiting	0	0/159 (0%)	2	2/163 (1%)
Cardiac disorders - Other, tachycardia	0	0/159 (0%)	2	2/163 (1%)
Measles	1	1/159 (1%)	0	0/163 (0%)
Drug resistant tuberculous meningitis	1	1/159 (1%)	0	0/163 (0%)

Fever and lymph node pain	1	1/159 (1%)	0	0/163 (0%)
Paraesthesia	1	1/159 (1%)	0	0/163 (0%)
Nervous system disorders - Other, cerebellar signs and paraesthesia	1	1/159 (1%)	0	0/163 (0%)
Hypersomnia	1	1/159 (1%)	0	0/163 (0%)
Hepatobiliary disorders - Other, liver mass	1	1/159 (1%)	0	0/163 (0%)
Hyperglycaemia	1	1/159 (1%)	0	0/163 (0%)
Nervous system disorders - Other, cerebellar signs and headache and fever	1	1/159 (1%)	0	0/163 (0%)
Nervous system disorders - Other, depressed consciousness and seizure	1	1/159 (1%)	0	0/163 (0%)
Gastrointestinal disorders - Other, greater than 5mls of fresh or changed blood aspirated from nasogastric tube	1	1/159 (1%)	0	0/163 (0%)
Lung infection and pneumothorax	1	1/159 (1%)	0	0/163 (0%)
Ileus	1	1/159 (1%)	0	0/163 (0%)
Metabolism and nutrition disorders - Other, diabetic ketoacidosis	1	1/159 (1%)	0	0/163 (0%)
Abdominal pain	1	1/159 (1%)	0	0/163 (0%)
Fatigue	1	1/159 (1%)	0	0/163 (0%)
Oedema	1	1/159 (1%)	0	0/163 (0%)
Gastrointestinal disorders - Other, vomiting and fatigue and anorexia	1	1/159 (1%)	0	0/163 (0%)
Musculoskeletal and connective tissue disorders - Other, temporomandibular dislocation	1	1/159 (1%)	0	0/163 (0%)
Respiratory, thoracic and mediastinal disorders - Other, chronic obstructive pulmonary disease	1	1/159 (1%)	0	0/163 (0%)
Haemoptysis	1	1/159 (1%)	0	0/163 (0%)
Nervous system disorders - Other, focal neurological sign and cerebral oedema	1	1/159 (1%)	0	0/163 (0%)
Oesophageal cancer	1	1/159 (1%)	0	0/163 (0%)
Nervous system disorders - Other, paraesthesia and blurred vision	1	1/159 (1%)	0	0/163 (0%)
Muscle cramp	1	1/159 (1%)	0	0/163 (0%)

Soft tissue infection	1	1/159 (1%)	0	0/163 (0%)
Nervous system disorders - Other, depressed consciousness and focal neurological sign	0	0/159 (0%)	1	1/163 (1%)
Musculoskeletal and connective tissue disorders - Other, jaw swelling	0	0/159 (0%)	1	1/163 (1%)
Infections and infestations - Other, dengue fever	0	0/159 (0%)	1	1/163 (1%)
Respiratory, thoracic and mediastinal disorders - Other, throat malignancy	0	0/159 (0%)	1	1/163 (1%)
Hypotension	0	0/159 (0%)	1	1/163 (1%)
Chest pain – cardiac	0	0/159 (0%)	1	1/163 (1%)
Hypernatraemia	0	0/159 (0%)	1	1/163 (1%)
Vision decreased	0	0/159 (0%)	1	1/163 (1%)
Hypoalbuminaemia	0	0/159 (0%)	1	1/163 (1%)
Hypomagnesaemia	0	0/159 (0%)	1	1/163 (1%)
Shingles	0	0/159 (0%)	1	1/163 (1%)
Conduction disorder	0	0/159 (0%)	1	1/163 (1%)
Lung infection and urinary tract infection	0	0/159 (0%)	1	1/163 (1%)
Nervous system disorders - Other, focal neurological sign, cranial nerve palsy	0	0/159 (0%)	1	1/163 (1%)
Acidosis	0	0/159 (0%)	1	1/163 (1%)
Blood bilirubin increased	0	0/159 (0%)	1	1/163 (1%)
Nervous system disorders - Other, headache and confusion	0	0/159 (0%)	1	1/163 (1%)
Dyspnoea	0	0/159 (0%)	1	1/163 (1%)
Haematoma	0	0/159 (0%)	1	1/163 (1%)

For any serious adverse event in CT genotype participants, at least one event occurred in 88/159 participants receiving dexamethasone, and at least one event occurred in 79/163 participants receiving placebo. p-value for number of participants with serious adverse event(s) = 0.22. p-value for total number of serious adverse events per participant = 1. Events summarized according to the System Organ Class of the Medical Dictionary for Regulatory Activities (MedDRA) hierarchy GCS=Glasgow coma scale. NOS=not otherwise specified.

Table S46. Serious adverse events and Grade 3 or 4 adverse events in the TT-genotype (N=89; all received dexamethasone)

Type of adverse event	n episode	n participants
TT genotype: Any selected adverse event	96	51/89 (57%)
Grade 3 adverse events	85	41/89 (46%)
Grade 4 adverse events	23	20/89 (22%)
Fall in GCS ≥ 2 points, for ≥ 48hrs	12	12/89 (13%)
Hypokalaemia	9	9/89 (10%)
Lung infection	8	8/89 (9%)
Urinary tract infection	5	5/89 (6%)
New focal neurological sign lasting >24 hours	5	5/89 (6%)
Elevation of blood transaminases ≥ 5 times the upper limit of normal or a rise in serum bilirubin >2.0mg/dL (>34 mol/L) without symptoms	5	4/89 (4%)
Elevation of blood transaminase concentrations ≥ 3 times the upper limit of normal with symptoms and signs of hepatitis (vomiting, abdominal pain, jaundice)	4	4/89 (4%)
Hyponatraemia requiring intervention or medically important	4	4/89 (4%)
Skin and subcutaneous tissue disorders - Other, TB drug rash	4	4/89 (4%)
Sepsis	4	3/89 (3%)
Headache	3	3/89 (3%)
Nervous system disorders - Other, worsening tuberculous meningitis	2	2/89 (2%)
Respiratory failure	2	2/89 (2%)
Respiratory, thoracic and mediastinal disorders - Other, asthma	2	2/89 (2%)
Upper gastrointestinal haemorrhage	2	2/89 (2%)
Seizure	2	2/89 (2%)
Depressed level of consciousness	2	2/89 (2%)
Fever	1	1/89 (1%)
Infections and infestations - Other, dengue fever	1	1/89 (1%)
Alanine aminotransferase increased	1	1/89 (1%)
Hypokalaemia	1	1/89 (1%)
Acute kidney injury	1	1/89 (1%)

**Type of adverse event**	**n episode**	**n participants**
Endocrine disorders - Other, polyuria	1	1/89 (1%)
Gastrointestinal disorders - Other, greater than 5mls of fresh or changed blood aspirated from nasogastric tube	1	1/89 (1%)
Cardiac disorders - Other, complete heart block	1	1/89 (1%)
Skin and subcutaneous tissue disorders - Other, rash of unknown aetiology	1	1/89 (1%)
Hypertension	1	1/89 (1%)
Gastrointestinal disorders - Other, intestinal obstruction	1	1/89 (1%)
Anaemia	1	1/89 (1%)
Nervous system disorders - Other, vision decreased	1	1/89 (1%)
Gastrointestinal disorders - Other, vomiting and abdominal pain	1	1/89 (1%)
White blood cells decreased	1	1/89 (1%)
Death NOS	1	1/89 (1%)
Nervous system disorders - Other, focal neurological sign and headache	1	1/89 (1%)
Dyspnoea	1	1/89 (1%)
Anorexia	1	1/89 (1%)
Vomiting	1	1/89 (1%)
Abdominal pain	1	1/89 (1%)
Hydrocephalus	1	1/89 (1%)

GCS=Glasgow coma scale. NOS=not otherwise specified.

Table S47: Summary of serious adverse events, shown by reasons for which they were considered serious, not shown by study arm

	Important medical event which may jeopardize the patient and/or require intervention (N=300)		Persistent or significant disability/ incapacity (N=17)		Prolong hospitalization (N=265)		Life threatening event (N=52)		Caused death (N=158)	
**Type of adverse event**	**n**	**Summary statistic**	**n**	**Summary statistic**	**n**	**Summary statistic**	**n**	**Summary statistic**	**N**	**Summary statistic**
Any adverse event	300		17		265		52		158	
- Fall in GCS ≥ 2 points, for ≥ 48hrs		10/300 (3%)		2/17 (12%)		15/265 (6%)		7/52 (13%)		67/158 (42%)
- New focal neurological sign lasting >24 hours		8/300 (3%)		10/17 (59%)		20/265 (8%)		2/52 (4%)		6/158 (4%)
- Hyponatraemia requiring intervention or medically important		45/300 (15%)		0/17 (0%)		8/265 (3%)		3/52 (6%)		2/158 (1%)
- Elevation of blood transaminases ≥ 5 times the upper limit of normal or a rise in serum bilirubin >2.0mg/dL (>34 mol/L) without symptoms		26/300 (9%)		0/17 (0%)		15/265 (6%)		0/52 (0%)		0/158 (0%)
- Elevation of blood transaminase concentrations ≥ 3 times the upper limit of normal with symptoms and signs of hepatitis (vomiting, abdominal pain, jaundice)		6/300 (2%)		0/17 (0%)		8/265 (3%)		1/52 (2%)		0/158 (0%)
- Abdominal pain		1/300 (0%)		0/17 (0%)		1/265 (0%)		0/52 (0%)		0/158 (0%)
- Acidosis		0/300 (0%)		0/17 (0%)		0/265 (0%)		0/52 (0%)		1/158 (1%)
- Acute kidney injury		2/300 (1%)		0/17 (0%)		4/265 (2%)		0/52 (0%)		0/158 (0%)
- Adrenal insufficiency		3/300 (1%)		0/17 (0%)		1/265 (0%)		1/52 (2%)		0/158 (0%)

	**Important medical event which may jeopardize the patient and/or require intervention (N=300)**		**Persistent or significant disability/ incapacity (N=17)**		**Prolong hospitalization (N=265)**		**Life threatening event (N=52)**		**Caused death (N=158)**	
**Type of adverse event**	**n**	**Summary statistic**	**n**	**Summary statistic**	**n**	**Summary statistic**	**n**	**Summary statistic**	**N**	**Summary statistic**
- Alanine aminotransferase increased		0/300 (0%)		0/17 (0%)		2/265 (1%)		0/52 (0%)		0/158 (0%)
- Anaemia		9/300 (3%)		0/17 (6%)		4/265 (2%)		0/52 (0%)		0/158 (0%)
- Anaphylaxis		0/300 (0%)		0/17 (0%)		0/265 (0%)		1/52 (2%)		0/158 (0%)
- Anorexia		0/300 (0%)		0/17 (0%)		1/265 (0%)		0/52 (0%)		0/158 (0%)
- Biliary tract infection		1/300 (0%)		0/17 (0%)		0/265 (0%)		0/52 (0%)		0/158 (0%)
- Blood and lymphatic system disorders - Other, haemophagocytic disorder		0/300 (0%)		0/17 (0%)		1/265 (0%)		0/52 (0%)		0/158 (0%)
- Blood antidiuretic hormone abnormal		1/300 (0%)		0/17 (0%)		0/265 (0%)		0/52 (0%)		0/158 (0%)
- Blood bilirubin increased		1/300 (0%)		0/17 (0%)		1/265 (0%)		0/52 (0%)		1/158 (1%)
- Blurred vision		2/300 (1%)		0/17 (0%)		3/265 (1%)		0/52 (0%)		0/158 (0%)
- Cardiac arrest		0/300 (0%)		0/17 (0%)		0/265 (0%)		0/52 (0%)		1/158 (1%)
- Cardiac disorders - Other, complete heart block		0/300 (0%)		0/17 (0%)		0/265 (0%)		0/52 (0%)		1/158 (1%)
- Cardiac disorders - Other, conduction disorder and hypokalaemia		0/300 (0%)		0/17 (0%)		1/265 (0%)		0/52 (0%)		0/158 (0%)
- Cardiac disorders - Other, tachycardia		0/300 (0%)		0/17 (0%)		0/265 (0%)		2/52 (4%)		0/158 (0%)
- Cataract		0/300 (0%)		0/17 (0%)		1/265 (0%)		0/52 (0%)		0/158 (0%)
- Cerebrospinal fluid leakage		1/300 (0%)		0/17 (0%)		2/265 (1%)		0/52 (0%)		0/158 (0%)

	Important medical event which may jeopardize the patient and/or require intervention (N=300)		Persistent or significant disability/ incapacity (N=17)		Prolong hospitalization (N=265)		Life threatening event (N=52)		Caused death (N=158)	
**Type of adverse event**	**n**	**Summary statistic**	**n**	**Summary statistic**	**n**	**Summary statistic**	**n**	**Summary statistic**	**N**	**Summary statistic**
- Chest pain - cardiac		0/300 (0%)		0/17 (0%)		1/265 (0%)		0/52 (0%)		0/158 (0%)
- Chest wall pain		0/300 (0%)		0/17 (0%)		1/265 (0%)		0/52 (0%)		0/158 (0%)
- Cholecystitis		1/300 (0%)		0/17 (0%)		0/265 (0%)		0/52 (0%)		0/158 (0%)
- Colitis		0/300 (0%)		0/17 (0%)		1/265 (0%)		0/52 (0%)		0/158 (0%)
- Conduction disorder		0/300 (0%)		0/17 (0%)		0/265 (0%)		1/52 (2%)		0/158 (0%)
- Cough		0/300 (0%)		0/17 (0%)		2/265 (1%)		0/52 (0%)		0/158 (0%)
- Creatinine increased		0/300 (0%)		0/17 (0%)		1/265 (0%)		1/52 (2%)		0/158 (0%)
- Death NOS		0/300 (0%)		0/17 (0%)		0/265 (0%)		0/52 (0%)		8/158 (5%)
- Depressed level of consciousness		0/300 (0%)		0/17 (0%)		1/265 (1%)		1/52 (2%)		11/158 (7%)
- Dizziness		2/300 (1%)		0/17 (0%)		0/265 (0%)		0/52 (0%)		0/158 (0%)
- Drug resistant tuberculous meningitis		1/300 (0%)		0/17 (0%)		0/265 (0%)		0/52 (0%)		0/158 (0%)
- Dyspnoea		1/300 (0%)		0/17 (0%)		0/265 (0%)		0/52 (0%)		1/158 (1%)
- Eczema		0/300 (0%)		0/17 (0%)		1/265 (0%)		0/52 (0%)		0/158 (0%)
- Elevated liver enzymes		1/300 (0%)		0/17 (0%)		1/265 (0%)		0/52 (0%)		0/158 (0%)
- Encephalomyelitis infection		0/300 (0%)		0/17 (0%)		1/265 (0%)		0/52 (0%)		0/158 (0%)
- Endocrine disorders - Other, polyuria		0/300 (0%)		0/17 (0%)		0/265 (0%)		2/52 (4%)		1/158 (1%)
- Eye disorders - Other, subconjunctival hemorrhage		1/300 (0%)		0/17 (0%)		0/265 (0%)		0/52 (0%)		0/158 (0%)
- Eye infection		0/300 (0%)		0/17 (0%)		1/265 (0%)		0/52 (0%)		0/158 (0%)
- Fatigue		0/300 (0%)		0/17 (0%)		1/265 (0%)		0/52 (0%)		0/158 (0%)

	Important medical event which may jeopardize the patient and/or require intervention (N=300)		Persistent or significant disability/ incapacity (N=17)		Prolong hospitalization (N=265)		Life threatening event (N=52)		Caused death (N=158)	
**Type of adverse event**	**n**	**Summary statistic**	**n**	**Summary statistic**	**n**	**Summary statistic**	**n**	**Summary statistic**	**N**	**Summary statistic**
- Fever		0/300 (0%)		0/17 (0%)		1/265 (0%)		0/52 (0%)		0/158 (0%)
- Fever and lymph node pain		0/300 (0%)		0/17 (0%)		1/265 (0%)		0/52 (0%)		0/158 (0%)
- Gastritis		1/300 (0%)		0/17 (0%)		0/265 (0%)		0/52 (0%)		0/158 (0%)
- Gastrointestinal disorders - Other, dysphagia and anorexia		0/300 (0%)		0/17 (0%)		1/265 (0%)		0/52 (0%)		0/158 (0%)
- Gastrointestinal disorders - Other, greater than 5mls of fresh or changed blood aspirated from nasogastric tube		2/300 (0%)		0/17 (0%)		0/265 (0%)		1/52 (2%)		0/158 (0%)
- Gastrointestinal disorders - Other, intestinal obstruction		1/300 (0%)		0/17 (0%)		0/265 (0%)		0/52 (0%)		0/158 (0%)
- Gastrointestinal disorders - Other, nausea and vomiting		1/300 (0%)		0/17 (0%)		0/265 (0%)		0/52 (0%)		0/158 (0%)
- Gastrointestinal disorders - Other, vomiting and abdominal pain		0/300 (0%)		0/17 (0%)		1/265 (0%)		0/52 (0%)		0/158 (0%)
- Gastrointestinal disorders - Other, vomiting and fatigue and anorexia		0/300 (0%)		0/17 (0%)		1/265 (0%)		0/52 (0%)		0/158 (0%)
- General disorders and administration site conditions - Other, fever and arm pain		1/300 (0%)		0/17 (0%)		0/265 (0%)		0/52 (0%)		0/158 (0%)

	**Important medical event which may jeopardize the patient and/or require intervention (N=300)**		**Persistent or significant disability/ incapacity (N=17)**		**Prolong hospitalization (N=265)**		**Life threatening event (N=52)**		**Caused death (N=158)**	
**Type of adverse event**	**n**	**Summary statistic**	**n**	**Summary statistic**	**n**	**Summary statistic**	**n**	**Summary statistic**	**N**	**Summary statistic**
- General disorders and administration site conditions - Other, withdraw from hospital treatment		0/300 (0%)		0/17 (0%)		0/265 (0%)		0/52 (0%)		2/158 (1%)
- Headache		1/300 (0%)		0/17 (0%)		9/265 (3%)		0/52 (0%)		0/158 (0%)
- Haematoma		0/300 (0%)		0/17 (0%)		1/265 (0%)		0/52 (0%)		0/158 (0%)
- Hemoptysis		0/300 (0%)		0/17 (0%)		1/265 (0%)		0/52 (0%)		0/158 (0%)
- Hepatobiliary disorders - Other, liver mass		1/300 (0%)		0/17 (0%)		0/265 (0%)		0/52 (0%)		0/158 (0%)
- Hepatobiliary disorders - Other, liver tumour		0/300 (0%)		0/17 (0%)		0/265 (0%)		0/52 (0%)		1/158 (1%)
- Hydrocephalus		5/300 (2%)		2/17 (12%)		4/265 (2%)		0/52 (0%)		1/158 (1%)
- Hyperglycaemia		0/300 (0%)		0/17 (0%)		2/265 (1%)		0/52 (0%)		0/158 (0%)
- Hypersomnia		0/300 (0%)		0/17 (0%)		1/265 (0%)		0/52 (0%)		0/158 (0%)
- Hypertension		1/300 (0%)		0/17 (0%)		1/265 (0%)		1/52 (2%)		1/158 (1%)
- Hyperuricaemia		2/300 (1%)		0/17 (0%)		0/265 (0%)		0/52 (0%)		0/158 (0%)
- Hypoalbuminaemia		0/300 (0%)		0/17 (0%)		1/265 (0%)		0/52 (0%)		0/158 (0%)
- Hypokalaemia		36/300 (12%)		0/17 (0%)		6/265 (2%)		0/52 (0%)		0/158 (0%)
- Hypomagnesaemia		0/300 (0%)		0/17 (0%)		2/265 (1%)		0/52 (0%)		0/158 (0%)
- Hypotension		0/300 (0%)		0/17 (0%)		0/265 (0%)		4/52 (8%)		3/158 (2%)
- Ileus		0/300 (0%)		0/17 (0%)		2/265 (1%)		0/52 (0%)		0/158 (0%)
- Infections and infestations - Other, COVID-19		1/300 (0%)		0/17 (0%)		4/265 (2%)		0/52 (0%)		2/158 (1%)

	Important medical event which may jeopardize the patient and/or require intervention (N=300)		Persistent or significant disability/ incapacity (N=17)		Prolong hospitalization (N=265)		Life threatening event (N=52)		Caused death (N=158)	
**Type of adverse event**	**n**	**Summary statistic**	**n**	**Summary statistic**	**n**	**Summary statistic**	**n**	**Summary statistic**	**N**	**Summary statistic**
- Infections and infestations - Other, dengue fever		0/300 (0%)		0/17 (0%)		3/265 (1%)		0/52 (0%)		0/158 (0%)
- Infections and infestations - Other, drug resistant tuberculosis		0/300 (0%)		0/17 (0%)		1/265 (0%)		0/52 (0%)		1/158 (1%)
- Infections and infestations - Other, superadded infection		0/300 (0%)		0/17 (0%)		1/265 (0%)		0/52 (0%)		0/158 (0%)
- Infections and infestations - Other, thigh abscess		1/300 (0%)		0/17 (0%)		1/265 (0%)		0/52 (0%)		0/158 (0%)
- Intracranial haemorrhage		0/300 (0%)		0/17 (0%)		0/265 (0%)		0/52 (0%)		3/158 (2%)
- Investigations - Other, pancytopaenia		2/300 (1%)		0/17 (0%)		0/265 (0%)		0/52 (0%)		0/158 (0%)
- Lung infection		30/300 (10%)		0/17 (0%)		14/265 (5%)		1/52 (2%)		12/158 (8%)
- Lung infection and pneumothorax		0/300 (0%)		0/17 (0%)		0/265 (0%)		1/52 (2%)		0/158 (0%)
- Lung infection and urinary tract infection		0/300 (0%)		0/17 (0%)		1/265 (0%)		0/52 (0%)		0/158 (0%)
- Lymph gland infection		0/300 (0%)		0/17 (0%)		2/265 (1%)		0/52 (0%)		0/158 (0%)
- Measles		0/300 (0%)		0/17 (0%)		1/265 (0%)		0/52 (0%)		0/158 (0%)
- Metabolism and nutrition disorders - Other, anorexia and vomiting		1/300 (0%)		0/17 (0%)		0/265 (0%)		0/52 (0%)		0/158 (0%)
- Metabolism and nutrition disorders -		0/300 (0%)		0/17 (0%)		0/265 (0%)		1/52 (2%)		0/158 (0%)

	Important medical event which may jeopardize the patient and/or require intervention (N=300)		Persistent or significant disability/ incapacity (N=17)		Prolong hospitalization (N=265)		Life threatening event (N=52)		Caused death (N=158)	
**Type of adverse event**	**n**	**Summary statistic**	**n**	**Summary statistic**	**n**	**Summary statistic**	**n**	**Summary statistic**	**N**	**Summary statistic**
Other, diabetic ketoacidosis										
- Metabolism and nutrition disorders - Other, malnutrition		2/300 (1%)		0/17 (0%)		0/265 (0%)		0/52 (0%)		0/158 (0%)
- Muscle cramp		0/300 (0%)		0/17 (0%)		1/265 (0%)		0/52 (0%)		0/158 (0%)
- Musculoskeletal and connective tissue disorders - Other, jaw swelling		0/300 (0%)		0/17 (0%)		1/265 (0%)		0/52 (0%)		0/158 (0%)
- Musculoskeletal and connective tissue disorders - Other, temporomandibular dislocation		0/300 (0%)		0/17 (0%)		1/265 (0%)		0/52 (0%)		0/158 (0%)
- Myelitis		0/300 (0%)		0/17 (0%)		0/265 (0%)		0/52 (0%)		1/158 (1%)
- Nervous system disorders - Other, cerebellar signs and headache and fever		0/300 (0%)		0/17 (0%)		1/265 (0%)		0/52 (0%)		0/158 (0%)
- Nervous system disorders - Other, cerebellar signs and paraesthesia		0/300 (0%)		0/17 (0%)		1/265 (0%)		0/52 (0%)		0/158 (0%)
- Nervous system disorders - Other, cerebellar signs and radiculitis		1/300 (0%)		0/17 (0%)		0/265 (0%)		0/52 (0%)		0/158 (0%)
- Nervous system disorders - Other,		1/300 (0%)		0/17 (0%)		1/265 (0%)		0/52 (0%)		0/158 (0%)

	Important medical event which may jeopardize the patient and/or require intervention (N=300)		Persistent or significant disability/ incapacity (N=17)		Prolong hospitalization (N=265)		Life threatening event (N=52)		Caused death (N=158)	
**Type of adverse event**	**n**	**Summary statistic**	**n**	**Summary statistic**	**n**	**Summary statistic**	**n**	**Summary statistic**	**N**	**Summary statistic**
depressed consciousness and hydrocephalus										
- Nervous system disorders - Other, depressed consciousness and focal neurological sign		0/300 (0%)		0/17 (0%)		0/265 (0%)		1/52 (2%)		0/158 (0%)
- Nervous system disorders - Other, depressed consciousness and seizure		0/300 (0%)		0/17 (0%)		0/265 (0%)		1/52 (2%)		0/158 (0%)
- Nervous system disorders - Other, spinal tuberculosis		1/300 (0%)		0/17 (0%)		1/265 (0%)		0/52 (0%)		0/158 (0%)
- Nervous system disorders - Other, focal neurological sign and headache		0/300 (0%)		0/17 (0%)		1/265 (0%)		0/52 (0%)		0/158 (0%)
- Nervous system disorders - Other, dizziness and vomiting		0/300 (0%)		0/17 (0%)		1/265 (0%)		0/52 (0%)		0/158 (0%)
- Nervous system disorders - Other, focal neurological sign		0/300 (0%)		1/17 (6%)		4/265 (2%)		0/52 (0%)		0/158 (0%)
- Nervous system disorders - Other, focal neurological sign and cerebral oedema		1/300 (0%)		0/17 (0%)		1/265 (0%)		0/52 (0%)		0/158 (0%)
- Nervous system disorders - Other, focal neurological sign and headache		0/300 (0%)		0/17 (0%)		1/265 (0%)		0/52 (0%)		0/158 (0%)

	Important medical event which may jeopardize the patient and/or require intervention (N=300)		Persistent or significant disability/ incapacity (N=17)		Prolong hospitalization (N=265)		Life threatening event (N=52)		Caused death (N=158)	
**Type of adverse event**	**n**	**Summary statistic**	**n**	**Summary statistic**	**n**	**Summary statistic**	**n**	**Summary statistic**	**N**	**Summary statistic**
- Nervous system disorders - Other, focal neurological sign, cranial nerve palsy		0/300 (0%)		0/17 (0%)		1/265 (0%)		0/52 (0%)		0/158 (0%)
- Nervous system disorders - Other, headache and hypertension		0/300 (0%)		0/17 (0%)		1/265 (0%)		0/52 (0%)		0/158 (0%)
- Nervous system disorders - Other, headache and vomiting		1/300 (0%)		0/17 (0%)		2/265 (1%)		0/52 (0%)		0/158 (0%)
- Nervous system disorders - Other, hydrocephalus and tuberculoma		2/300 (1%)		0/17 (0%)		1/265 (0%)		0/52 (0%)		0/158 (0%)
- Nervous system disorders - Other, subdural hematoma		0/300 (0%)		0/17 (0%)		2/265 (1%)		0/52 (0%)		0/158 (0%)
- Nervous system disorders - Other, tuberculoma		0/300 (0%)		0/17 (0%)		1/265 (0%)		0/52 (0%)		0/158 (0%)
- Nervous system disorders - Other, worsening tuberculous meningitis		4/300 (1%)		0/17 (0%)		6/265 (2%)		0/52 (0%)		0/158 (0%)
- Nervous system disorders - Other, blurred vision		1/300 (0%)		0/17 (0%)		0/265 (0%)		0/52 (0%)		0/158 (0%)
- Nervous system disorders - Other, headache and confusion		0/300 (0%)		0/17 (0%)		1/265 (0%)		0/52 (0%)		0/158 (0%)

	**Important medical event which may jeopardize the patient and/or require intervention (N=300)**		**Persistent or significant disability/ incapacity (N=17)**		**Prolong hospitalization (N=265)**		**Life threatening event (N=52)**		**Caused death (N=158)**	
**Type of adverse event**	**n**	**Summary statistic**	**n**	**Summary statistic**	**n**	**Summary statistic**	**n**	**Summary statistic**	**N**	**Summary statistic**
- Nervous system disorders - Other, paraesthesia and blurred vision		0/300 (0%)		0/17 (0%)		1/265 (0%)		0/52 (0%)		0/158 (0%)
- Nervous system disorders - Other, vision decreased		0/300 (0%)		0/17 (0%)		1/265 (0%)		0/52 (0%)		0/158 (0%)
- Oedema		0/300 (0%)		0/17 (0%)		1/265 (1%)		0/52 (0%)		0/158 (0%)
- Oedema cerebral		1/300 (0%)		0/17 (0%)		2/265 (1%)		1/52 (2%)		0/158 (0%)
- Oedema limbs		0/300 (0%)		0/17 (0%)		1/265 (0%)		0/52 (0%)		0/158 (0%)
- Oesophageal cancer		0/300 (0%)		0/17 (0%)		0/265 (0%)		0/52 (0%)		1/158 (1%)
- Oesophageal fistula		0/300 (0%)		0/17 (0%)		1/265 (0%)		0/52 (0%)		0/158 (0%)
- Paraesthesia		0/300 (0%)		0/17 (0%)		2/265 (1%)		0/52 (0%)		0/158 (0%)
- Pleural effusion		1/300 (0%)		0/17 (0%)		1/265 (0%)		0/52 (0%)		1/158 (1%)
- Respiratory failure		2/300 (1%)		0/17 (0%)		2/265 (1%)		7/52 (13%)		11/158 (7%)
- Respiratory, thoracic and mediastinal disorders - Other, asthma		1/300 (0%)		0/17 (0%)		1/265 (0%)		0/52 (0%)		0/158 (0%)
- Respiratory, thoracic and mediastinal disorders - Other, chronic obstructive pulmonary disease		0/300 (0%)		0/17 (0%)		1/265 (0%)		0/52 (0%)		0/158 (0%)
- Respiratory, thoracic and mediastinal disorders - Other, throat malignancy		0/300 (0%)		0/17 (0%)		1/265 (0%)		0/52 (0%)		0/158 (0%)
- Seizure		1/300 (0%)		0/17 (0%)		4/265 (2%)		1/52 (2%)		2/158 (1%)
- Sepsis		8/300 (3%)		0/17 (0%)		1/265 (0%)		5/52 (10%)		8/158 (5%)

	**Important medical event which may jeopardize the patient and/or require intervention (N=300)**		**Persistent or significant disability/ incapacity (N=17)**		**Prolong hospitalization (N=265)**		**Life threatening event (N=52)**		**Caused death (N=158)**	
**Type of adverse event**	**n**	**Summary statistic**	**n**	**Summary statistic**	**n**	**Summary statistic**	**n**	**Summary statistic**	**N**	**Summary statistic**
- Shingles		1/300 (0%)		0/17 (0%)		2/265 (0%)		0/52 (0%)		0/158 (0%)
- Skin and subcutaneous tissue disorders - Other, rash of unknown aetiology		7/300 (2%)		0/17 (0%)		4/265 (2%)		0/52 (0%)		0/158 (0%)
- Skin and subcutaneous tissue disorders - Other, TB drug rash		6/300 (2%)		0/17 (0%)		7/265 (3%)		0/52 (0%)		0/158 (0%)
- Skin infection		1/300 (0%)		0/17 (0%)		0/265 (0%)		0/52 (0%)		0/158 (0%)
- Skin ulceration		0/300 (0%)		0/17 (0%)		3/265 (1%)		0/52 (0%)		0/158 (0%)
- Soft tissue infection		0/300 (0%)		0/17 (0%)		1/265 (0%)		0/52 (0%)		0/158 (0%)
- Stroke		0/300 (0%)		1/17 (6%)		3/265 (1%)		0/52 (0%)		0/158 (0%)
- Supraventricular tachycardia		0/300 (0%)		0/17 (0%)		1/265 (0%)		0/52 (0%)		0/158 (0%)
- Thromboembolic event		0/300 (0%)		0/17 (0%)		0/265 (0%)		1/52 (2%)		1/158 (1%)
- Upper gastrointestinal haemorrhage		8/300 (3%)		0/17 (0%)		3/265 (0%)		3/52 (6%)		4/158 (3%)
- Urinary tract infection		37/300 (12%)		1/17 (6%)		18/265 (7%)		1/52 (2%)		3/158 (2%)
- Viraemia		0/300 (0%)		0/17 (0%)		1/265 (0%)		0/52 (0%)		0/158 (0%)
- Vision decreased		0/300 (0%)		0/17 (0%)		1/265 (0%)		0/52 (0%)		0/158 (0%)
- Vomiting		1/300 (0%)		0/17 (0%)		6/265 (2%)		0/52 (0%)		0/158 (0%)
- White blood cell decreased		0/300 (0%)		0/17 (0%)		1/265 (0%)		0/52 (0%)		0/158 (0%)

Table S48. Summary of serious adverse events possibly, probably, or definitely related to the study drug in CC genotype participants

	**Dexamethasone (N=146)**		**Placebo (N=145)**	
**Type of adverse event**	**n episode**	**n participants**	**n episode**	**n participants**
CC genotype: Any serious adverse event	21	13/146 (9%)	21	18/145 (12%)
Hypokalaemia	6	5/146 (3%)	7	6/145 (4%)
Upper gastrointestinal haemorrhage	4	3/146 (2%)	3	3/145 (2%)
Urinary tract infection	5	3/146 (2%)	1	1/145 (1%)
Lung infection	1	1/146 (1%)	2	2/145 (1%)
Fall in GCS ≥ 2 points, for ≥ 48hrs	0	0/146 (0%)	2	2/145 (1%)
Elevation of blood transaminases ≥ 5 times the upper limit of normal or a rise in serum bilirubin >2.0mg/dL (>34 mol/L) without symptoms	1	1/146 (1%)	0	0/145 (0%)
Gastrointestinal disorders - Other, greater than 5mls of fresh or changed blood aspirated from nasogastric tube	1	1/146 (1%)	0	0/145 (0%)
Skin and subcutaneous tissue disorders - Other, rash of unknown aetiology	1	1/146 (1%)	0	0/145 (0%)
Gastritis	1	1/146 (1%)	0	0/145 (0%)
Oedema cerebral	1	1/146 (1%)	0	0/145 (0%)
Nervous system disorders - Other, tuberculoma	0	0/146 (0%)	1	1/145 (1%)
New focal neurological sign lasting >24 hours	0	0/146 (0%)	1	1/145 (1%)
Myelitis	0	0/146 (0%)	1	1/145 (1%)
Adrenal insufficiency	0	0/146 (0%)	1	1/145 (1%)
Hyponatraemia requiring intervention or medically important	0	0/146 (0%)	1	1/145 (1%)
Hydrocephalus	0	0/146 (0%)	1	1/145 (1%)

Table S49. Summary of serious adverse events possibly, probably, or definitely related to the study drug in CT-genotype participants

	Dexamethasone (N=159)		Placebo (N=163)	
**Type of adverse event**	**n episode**	**n participants**	**n episode**	**n participants**
CT genotype: Any serious adverse event	19	15/159 (9%)	28	13/163 (8%)
Urinary tract infection	3	3/159 (2%)	5	4/163 (2%)
Hypokalaemia	3	3/159 (3%)	5	4/163 (2%)
Hyponatraemia requiring intervention or medically important	2	2/159 (1%)	4	3/163 (2%)
Lung infection	0	0/159 (0%)	5	3/163 (1%)
Anaemia	1	1/159 (1%)	3	3/163 (2%)
Upper gastrointestinal haemorrhage	2	2/159 (1%)	1	1/163 (1%)
Sepsis	1	1/159 (1%)	2	2/163 (1%)
New focal neurological sign lasting >24 hours	1	1/159 (1%)	1	1/163 (1%)
Nervous system disorders - Other, tuberculoma	1	1/159 (1%)	0	0/163 (0%)
Skin and subcutaneous tissue disorders - Other, TB drug rash	1	1/159 (1%)	0	0/163 (0%)
Nervous system disorders - Other, tuberculoma and hydrocephalus	1	1/159 (1%)	0	0/163 (0%)
Gastrointestinal disorders - Other, greater than 5mls of fresh or changed blood aspirated from nasogastric tube	1	1/159 (1%)	0	0/163 (0%)
Ileus	1	1/159 (1%)	0	0/163 (0%)
Hydrocephalus	1	1/159 (1%)	0	0/163 (0%)
Cardiac disorders – Other, tachycardia	0	0/159 (0%)	1	1/163 (1%)
Adrenal insufficiency	0	0/159 (0%)	1	1/163 (1%)

Table S50. Summary of serious adverse events possibly, probably, or definitely related to the study drug in TT-genotype participants (N=89; all treated with dexamethasone)

Type of adverse event	n episode	n participants
TT genotype: Any adverse event leading to TB treatment interruption	16	12/89 (13%)
Hypokalaemia	6	6/89 (7%)
Upper gastrointestinal haemorrhage	2	2/89 (2%)
Nervous system disorders – Other, worsening tuberculous meningitis	1	1/89 (1%)
Urinary tract infection	1	1/89 (1%)
Gastrointestinal disorders - Other, greater than 5mls of fresh or changed blood aspirated from nasogastric tube	1	1/89 (1%)
Respiratory. Thoracic and mediastinal disorders – Other, rash of unknown etiology	1	1/89 (1%)
Hypertension	1	1/89 (1%)
New focal neurological sign lasting >24 hours	1	1/89 (1%)
Anaemia	1	1/89 (1%)

Table S51. Summary of Grade 3&4 laboratory abnormalities in CC- and CT- and TT-genotype participants

	Dexamethasone (N = 394)		Placebo (N = 308)	
**Type of adverse event**	**n episode**	**n participants**	**n episode**	**n participants**
**CC genotype: Any grade 3&4 laboratory abnormality**	597	137/146 (94%)	669	133/145 (92%)
- Grade 3	555	136/146 (93%)	612	133/145 (92%)
- Grade 4	42	27/146 (18%)	57	22/145 (15%)
Creatinine increased	322	131/146 (90%)	343	127/145 (88%)
Hyponatraemia	136	51/146 (35%)	176	64/145 (44%)
Hypokalaemia	102	60/146 (41%)	100	44/145 (30%)
ALT increased	21	15/146 (10%)	30	18/145 (12%)
Anaemia	16	6/146 (4%)	20	13/145 (9%)
**CT genotype: Any grade 3&4 laboratory abnormality**	612	148/159 (93%)	796	150/163 (92%)
- Grade 3	571	148/159 (93%)	744	150/163 (92%)
- Grade 4	41	26/159 (16%)	52	27/163 (17%)
Creatinine increased	334	143/159 (90%)	405	147/163 (90%)
Hyponatraemia	135	51/159 (32%)	195	67/163 (41%)
Hypokalaemia	96	47/159 (30%)	135	50/163 (31%)
Anaemia	22	10/159 (6%)	39	10/163 (6%)
ALT increased	24	16/159 (10%)	22	18/163 (11%)
Hypoglycaemia	1	1/159 (1%)	0	0/163 (0%)
**TT genotype: Any grade 3&4 laboratory abnormality**	322	81/89 (91%)		
- Grade 3	297	81/89 (91%)		
- Grade 4	25	15/89 (17%)		
Creatinine increased	178	80/89 (90%)		
Hypokalaemia	70	36/89 (40%)		
Hyponatraemia	56	28/89 (31%)		
ALT increased	9	7/89 (8%)		

	Dexamethasone (N = 394)		Placebo (N = 308)	
**Type of adverse event**	**n episode**	**n participants**	**n episode**	**n participants**
Anaemia	9	6/89 (7%)		

ALT=alanine transaminase

Table S52. Exploratory analysis of associations of day 30 CSF cytokines (a) and CSF inflammatory pathway expression (b), with the primary outcome, with and without adjustment for LTA4H genotype, dexamethasone/placebo allocation, and baseline cytokines

a)

CSF cytokines/pathways	Primary outcome = No N = 287		Primary outcome = Yes N = 58		Comparison with adjustment[1]		Comparison without adjusment[2]	
	N	Summary statistic	N	Summary statistic	HR (95% CI)	Adjusted P	HR (95% CI)	Adjusted P
TNF	287	-0.07 (-1.78, 2.25)	58	2.18 (-1.34, 3.81)	1.17 (1, 1.36)	0.131	1.2 (1.08, 1.33)	0.004
IL-1β	287	0.32 (-1.66, 3.08)	58	1.79 (-0.59, 4.64)	1.14 (1.02, 1.27)	0.091	1.13 (1.03, 1.23)	0.024
IL-6	287	1.67 (-0.88, 5.12)	58	4.14 (0.17, 6.27)	1.11 (1, 1.22)	0.131	1.12 (1.03, 1.21)	0.024
IL-12β	287	0.87 (-1.61, 1.91)	58	1.34 (-1.19, 2.76)	1.07 (0.91, 1.25)	0.44	1.12 (0.99, 1.25)	0.064
IFN-γ	287	2.4 (-0.1, 7.6)	58	6.3 (1.5, 8.8)	1.12 (1.01, 1.25)	0.131	1.12 (1.04, 1.19)	0.008
IL-10	287	0.4 (-2.7, 3.0)	58	2.2 (-2.0, 4.2)	1.17 (1.05, 1.31)	0.032	1.11 (1.03, 1.2)	0.024

b)

CSF pathways	Primary outcome = No N = 287		Primary outcome = Yes N = 58		Comparison with adjustment[1]		Comparison without adjusment[2]	
	N	Summary statistic	N	Summary statistic	HR (95% CI)	Adjusted P	HR (95% CI)	Adjusted P
IFN Signaling	287	-1.75 (-2.74, -0.01)	58	-0.64 (-1.86, 1.13)	1.31 (1.11, 1.54)	0.006	1.29 (1.14, 1.46)	2.99E-04
Neutrophil degranulation	287	-2.4 (-4.6, 0.7)	58	0.0 (-2.6, 2.6)	1.2 (1.08, 1.33)	0.006	1.17 (1.08, 1.27)	3.90E-04
Neutrophil activation	287	-2.12 (-4.25, 0.79)	58	0.15 (-2.40, 2.34)	1.21 (1.08, 1.36)	0.006	1.18 (1.08, 1.29)	3.90E-04
Eicosanoid Metabolic Process	287	-0.86 (-1.51, 0.00)	58	-0.18 (-1.04, 0.66)	1.47 (1.14, 1.89)	0.008	1.51 (1.23, 1.85)	3.04E-04
TNF Signaling	287	-2.15 (-3.69, 0.48)	58	-0.02 (-2.27, 1.80)	1.18 (1.04, 1.34)	0.009	1.19 (1.09, 1.31)	3.90E-04
Cytokine Signaling	287	-3.9 (-7.3, 1.0)	58	-0.2 (-4.5, 4.0)	1.1 (1.03, 1.17)	0.008	1.09 (1.05, 1.14)	3.04E-04

The primary outcome was death or new neurological event by 12 months from randomization. Panel (a): CSF cytokine levels (NPX-Log2 normalized protein expression) are presented as median (interquartile range). Mean difference was calculated as the mean in the group with the primary outcome event minus the mean in the group without the primary outcome, and is presented with corresponding 95% confidence intervals. [1] Cox regression was used to assess associations between CSF cytokines at day 30 and the primary outcome. Only patients who survived to day 30 and had available data were included (n = 345), with outcomes analyzed from that point onward. Models were adjusted for genotype, treatment arm, and baseline cytokine levels. P-values were corrected for multiple comparisons using the Benjamini–Hochberg method. [2] Cox regression was used to assess associations between CSF cytokines at day 30 and the primary outcome. Only patients who survived to day 30 and had available data were included (n = 345), with outcomes analyzed from that point onward. Models were unadjusted for confounding, and p-values were corrected using the Benjamini–Hochberg method.

Panel (b): CSF inflammatory pathways (enrichment score) are presented as median (interquartile range). Per-sample pathway enrichment scores were calculated using the GSVA z-score method, in which cytokine expression values (NPX) were standardized (mean = 0, SD = 1) across samples and then averaged within each pathway, with the average rescaled by the square root of the number of genes in the pathway. Mean difference was calculated as the mean in the group with the primary outcome event minus the mean in the group without the primary outcome, and is presented with corresponding 95% confidence intervals. [1] Cox regression was used to assess associations between CSF inflammatory pathways at day 30 and the primary outcome. Only patients who survived to day 30 and had available data were included (n = 345), with outcomes analyzed from that point onward. Models were adjusted for genotype, treatment arm, and baseline cytokine levels. P-values were corrected for multiple comparisons using the Benjamini–Hochberg method. [2] Cox regression was used to assess associations between CSF inflammatory pathways at day 30 and the primary outcome. Only patients who survived to day 30 and had available data were included (n = 345), with outcomes analyzed from that point onward. Models were unadjusted for confounding, and p-values were corrected using the Benjamini–Hochberg method.

CI=confidence interval. CSF=cerebrospinal fluid. HR=hazard ratio. IFN=interferon. IL=interleukin. IQR=interquartile range. LTA4H=leukotriene A4 hydrolase. N=number of participants. TNF=tumour necrosis factor.

Table S53. Baseline characteristics of the two trials included in the individual participant data meta-analysis

Characteristic	N	LAST ACT, N = 702	Thwaites 2004,[5] N = 447
Age (years)	1,149	46.5 (33.2, 58.9)	39.0 (27.0, 57.0)
Treatment arm	1,149		
Placebo		308 (43.9%)	217 (48.5%)
Dexamethasone		394 (56.1%)	230 (51.5%)
TBM diagnosis	1,149		
Definite		312 (44.4%)	97 (21.7%)
Probable or possible		390 (55.6%)	346 (77.4%)
Confirmed non-TBM		0	4 (0.9%)
MRC Grade	1,149		
Grade I		298 (42.5%)	151 (33.8%)
Grade II		339 (48.7%)	201 (45.0%)
Grade III		61 (8.8%)	95 (21.3%)
Gender	1,149		
Female		267 (38.0%)	203 (45.4%)
Male		435 (62.0%)	244 (54.6%)
Anti-tuberculosis drug resistance	1,148		
Multi-drug resistant or rifampin mono-resistant		6 (0.9%)	5 (1.1%)
Isoniazid resistant without rifampin resistance		39 (5.6%)	24 (5.4%)
No or other resistance		130 (18.5%)	89 (19.9%)
Mtb not isolated or missing result		526 (74.9%)	329 (73.6%)

Mtb=Mycobacterium tuberculosis. TBM=tuberculous meningitis

Table S54. Survival over the first 9 months after randomization, by study and arm

Endpoint	Dexamethasone (n=624)	Placebo (n=525)	Comparison	
	events/n (risk [%])	events/n (risk [%])	RMTL difference (95%CI); p-value	HR (95%CI); p-value
**Death within 9 months after randomization**	135/624 (21.6)	138/525 (26.3)	-0.42 (-0.79, -0.04); p=0.028	0.78 (0.62, 0.99); p=0.044

Table S55. Diagnostic criteria for tuberculous meningitis[6]

		Diagnostic score
**Clinical criteria**	(Maximum category score=6)	
	Symptom duration of >5 days	4
	Systemic symptoms suggestive of tuberculosis (one or more of the following): weight loss (or poor weight gain in children), night sweats, or persistent cough for >2 weeks	2
	History of recent (within past year) close contact with an individual with pulmonary tuberculosis or a positive TST or IGRA (only in children <10 years of age)	2
	Focal neurological deficit (excluding cranial nerve palsies)	1
	Cranial nerve palsy	1
	Altered consciousness	1
**CSF criteria**	(Maximum category score=4)	
	Clear appearance	1
	Cells: 10-500 per µl	1
	Lymphocytic predominance (>50%)	1
	Protein concentration >1 g/L	1
	CSF to plasma glucose ratio of less than 50% or an absolute CSF glucose concentration less than 2.2mmol/L	1
**Cerebral imaging criteria**	(Maximum category score=6)	
	Hydrocephalus	1
	Basal meningeal enhancement	2
	Tuberculoma	2
	Infarct	1
	Pre-contrast basal hyperdensity	2
**Evidence of tuberculosis elsewhere**	(Maximum category score=4)	

	Chest radiograph suggestive of active tuberculosis: signs of tuberculosis=2; miliary tuberculosis=4	2 or 4
	CT/ MRI/ ultrasound evidence for tuberculosis outside the CNS	2
	AFB identified or *Mycobacterium tuberculosis* cultured from another source-i.e., sputum, lymph node, gastric washing, urine, blood culture	4
	Positive commercial *M. tuberculosis* NAAT from extra-neural specimen	4

Diagnostic criteria based on total score:

Definite TBM: acid-fast bacilli seen in CSF or *M. tuberculosis* cultured or detected by commercial NAAT in CSF

Probable TBM: score >9 (if no brain imaging) or >11 (if brain imaging)

Possible TBM: score 6-9 (if no brain imaging) or 6-11 (if brain imaging)

Not TBM: score <6 and a confirmed alternative diagnosis identified, or if the participant recovered without TB drugs. Individuals who do not meet these criteria for 'Not TBM' are classified as 'possible TBM'.

AFB=acid-fast bacilli. CNS=central nervous system. CSF=cerebrospinal fluid. CT=computed tomography. MRI=magnetic resonance imaging. NAAT=nucleic acid amplification test. TBM=tuberculous meningitis.

Table S56. The Modified Rankin Scale

Score	Description
0	No symptoms
1	Minor symptoms not interfering with lifestyle
2	Symptoms that lead to some restriction in lifestyle, but do not interfere with the patient's ability to look after themselves
3	Symptoms that restrict lifestyle and prevent totally independent living
4	Symptoms that clearly prevent independent living, although the patient does not need constant care and attention.
5	Totally dependent, requiring constant help day and night.
6	Death

References

1. Lee, E., Chuang, H.Y., Kim, J.W., Ideker, T. & Lee, D. Inferring pathway activity toward precise disease classification. *PLoS Comput Biol* **4**, e1000217 (2008).
2. Dodd, P.J.*, et al.* The global burden of tuberculous meningitis in adults: A modelling study. *PLOS Glob Public Health* **1**, e0000069 (2021).
3. du Preez, K.*, et al.* Global burden of tuberculous meningitis in children aged 0-14 years in 2019: a mathematical modelling study. *Lancet Glob Health* **13**, e59-e68 (2025).
4. Huynh, J.*, et al.* Tuberculous meningitis: progress and remaining questions. *Lancet Neurol* **21**, 450-464 (2022).
5. Thwaites, G.E.*, et al.* Dexamethasone for the treatment of tuberculous meningitis in adolescents and adults. *N Engl J Med* **351**, 1741-1751 (2004).
6. Marais, S.*, et al.* Tuberculous meningitis: a uniform case definition for use in clinical research. *Lancet Infect Dis* **10**, 803-812 (2010).
