# Supplementary material for: Genotype-stratified adjunctive dexamethasone for tuberculous meningitis in HIV-negative adults: a randomized controlled phase 3 trial
Source: Nat Med. 2026 Jan 15;32(3):849–58. doi: 10.1038/s41591-025-04138-z (PMC13004695; doi:10.1038/s41591-025-04138-z)
Supplement: Supplementary file 2 — Reporting Summary [file 41591_2025_4138_MOESM2_ESM.pdf]

nature portfolio

Corresponding author(s): Joseph Donovan

Last updated by author(s): Jun 5, 2025

Reporting Summary

Nature Portfolio wishes to improve the reproducibility of the work that we publish. This form provides structure for consistency and transparency in reporting. For further information on Nature Portfolio policies, see our Editorial Policies and the Editorial Policy Checklist.

Statistics

For all statistical analyses, confirm that the following items are present in the figure legend, table legend, main text, or Methods section.

n/a	Confirmed	
☐	☒	The exact sample size (*n*) for each experimental group/condition, given as a discrete number and unit of measurement
☒	☐	A statement on whether measurements were taken from distinct samples or whether the same sample was measured repeatedly
☐	☒	The statistical test(s) used AND whether they are one- or two-sided<br>*Only common tests should be described solely by name; describe more complex techniques in the Methods section.*
☐	☒	A description of all covariates tested
☐	☒	A description of any assumptions or corrections, such as tests of normality and adjustment for multiple comparisons
☐	☒	A full description of the statistical parameters including central tendency (e.g. means) or other basic estimates (e.g. regression coefficient) AND variation (e.g. standard deviation) or associated estimates of uncertainty (e.g. confidence intervals)
☐	☒	For null hypothesis testing, the test statistic (e.g. *F*, *t*, *r*) with confidence intervals, effect sizes, degrees of freedom and *P* value noted<br>*Give P values as exact values whenever suitable.*
☒	☐	For Bayesian analysis, information on the choice of priors and Markov chain Monte Carlo settings
☐	☒	For hierarchical and complex designs, identification of the appropriate level for tests and full reporting of outcomes
☒	☐	Estimates of effect sizes (e.g. Cohen's *d*, Pearson's *r*), indicating how they were calculated

Our web collection on statistics for biologists contains articles on many of the points above.

Software and code

Policy information about availability of computer code

Data collection	Data not collected using code
Data analysis	Data were analysed using the program R (version 4.4.2; R Core Team, 2024). R code is provided in GitHub with a link provided in the manuscript. GitHub - oucru-biostats/LAST-ACT: Analysis code for LAST ACT trial

For manuscripts utilizing custom algorithms or software that are central to the research but not yet described in published literature, software must be made available to editors and reviewers. We strongly encourage code deposition in a community repository (e.g. GitHub). See the Nature Portfolio guidelines for submitting code & software for further information.

Data

Policy information about availability of data

All manuscripts must include a data availability statement. This statement should provide the following information, where applicable:

- Accession codes, unique identifiers, or web links for publicly available datasets
- A description of any restrictions on data availability
- For clinical datasets or third party data, please ensure that the statement adheres to our policy

De-identified trial participant data (including data dictionaries) will be shared on request to the Oxford University Clinical Research Unit data access committee (via emailing the senior author, GT, or corresponding author, JD). A response will be provided within four weeks of receiving the request. The data shared will allow replication of the primary analysis contained within the current manuscript. The study protocol and statistical analysis plans have been published and are available

under open access. Proteomic and transcriptomic data used for this analysis have been deposited into a public repository and can be found at https://doi.org/10.5061/dryad.f1vhhmh7v.

Research involving human participants, their data, or biological material

Policy information about studies with human participants or human data. See also policy information about sex, gender (identity/presentation), and sexual orientation and race, ethnicity and racism.

Reporting on sex and gender	Sex and gender were not considered in study design.<br>Sex- and gender-based analyses were not pre-planned.<br>Sex was determined based on self reporting. Gender data were not also collected.<br>Baseline data are shown by participant sex.<br>Whilst males are more commonly affected, tuberculous meningitis pathophysiology is not known to vary by sex, therefore analyses by sex were not performed.
Reporting on race, ethnicity, or other socially relevant groupings	Tuberculous meningitis occurs in all ethnic groups.<br>These data do not report on groups by race, ethnicity, nor social groupings.
Population characteristics	These are described in full in Table 1 (Baseline characteristics in the intention-to-treat population, including TT-genotype).
Recruitment	We recruited participants from the Hospital for Tropical Diseases, and Pham Ngoc Thach Hospital for Tuberculosis and Lung Disease, in Ho Chi Minh City, Vietnam. Written informed consent to enter the trial was obtained from all participants or a relative if they were incapacitated. If capacity returned, consent from the participant was obtained. Participants were ≥18 years old, HIV-negative, with a clinical diagnosis of tuberculous meningitis (≥5 days of meningitis symptoms and consistent cerebrospinal fluid abnormalities) with anti-tuberculosis chemotherapy either planned or started by the treating clinician. Participants were subsequently classified as having definite, probable, or possible tuberculous meningitis, following published diagnostic criteria (Table S63). Patients were ineligible if another brain infection was confirmed or suspected, if >6 consecutive days of anti-tuberculosis chemotherapy or systemic corticosteroids were received immediately before enrolment, or if systemic corticosteroids were considered mandatory or contraindicated for any reason.
Ethics oversight	Ethical approvals for the LAST ACT trial (with approval numbers) were as follows:<br>1. The Oxford Tropical Research Ethics Committee: 52-16<br>2. The ethical committee of the Hospital for Tropical Diseases: 37/HDDD<br>3. The ethical committee of Pham Ngoc Thach Hospital for Tuberculosis and Lung Disease: 1034/HDDD-PNT<br>4. The Vietnam Ministry of Health: 151/CN-BDGDD

Note that full information on the approval of the study protocol must also be provided in the manuscript.

Field-specific reporting

Please select the one below that is the best fit for your research. If you are not sure, read the appropriate sections before making your selection.

☒ Life sciences ☐ Behavioural & social sciences ☐ Ecological, evolutionary & environmental sciences

For a reference copy of the document with all sections, see nature.com/documents/nr-reporting-summary-flat.pdf

Life sciences study design

All studies must disclose on these points even when the disclosure is negative.

Sample size	We adopted a hybrid trial-design approach that aimed to prove non-inferiority of placebo first, but also superiority of placebo should dexamethasone prove harmful. The trial had two primary populations: the combined CC/CT-genotype population (designated 2% of the one-sided type I error of 2.5%) and the CC-genotype population (designated 0.86% of the error).26 We set the non-inferiority margin in favour of dexamethasone at a hazard ratio of 0.75 and assumed a true hazard ratio of 1.15 in the CC/CT-genotype population. To obtain 80% power at the one-sided 2% significance level, 184 events in the CC/CT-genotype population would be required. Assuming a 12-month risk of the primary endpoint in the dexamethasone arm of 35%, a hazard ratio of 1.15 corresponds to a risk of 31.2% of placebo, and the non-inferiority margin implies that we can exclude an absolute risk increase of placebo of (at worst) +8.7%. Assuming an overall event risk of ≥32%, and 11% sample increase to compensate for loss-to-follow-up, we aimed to randomize 640 CC/CT-genotype participants. Anticipating 10% being LTA4H TT-genotype, we planned to enrol 720 participants in total.
Data exclusions	Data provided in Figure 1 Screening, enrollment and randomization). 1030 patients assessed for eligibility. 310 patients excluded (reasons in Figure 1), and 720 enrolled. 18 participants then excluded before randomization (reasons listed in Figure 1).
Replication	N/A
Randomization	Randomization occurred once LTA4H genotyping results were available, usually within 24 hours. LTA4H CC/CT-genotype participants were randomized to two parallel groups in a 1:1 ratio: dexamethasone or placebo for 6-8-weeks. TT-genotype participants received open-label dexamethasone for 6-8 weeks. Randomization was stratified by participating hospital, LTA4H genotype, and modified MRC disease severity grade assessed at enrolment. Participants in grade I had a Glasgow Coma Score of 15 (possible range, 3 to 15, with higher scores indicating better status) with no focal neurologic signs; grade II participants had a score of either 11 to 14, or had focal neurological signs; and grade III participants had a score of 10 or less. The randomization list was computer-generated based on random permuted blocks with block size 4

	and 6 (probability 0.75 and 0.25). Participant randomization was performed by trained clinical staff using a web-based software, with 24-hour availability.
Blinding	Blinded fully made-up and labelled study treatment packs contained either dexamethasone or identical placebo. All participants and investigators were blinded to study drug allocation

Reporting for specific materials, systems and methods

We require information from authors about some types of materials, experimental systems and methods used in many studies. Here, indicate whether each material, system or method listed is relevant to your study. If you are not sure if a list item applies to your research, read the appropriate section before selecting a response.

Materials & experimental systems

n/a	Involved in the study
☒	☐ Antibodies
☒	☐ Eukaryotic cell lines
☒	☐ Palaeontology and archaeology
☒	☐ Animals and other organisms
☐	☒ Clinical data
☒	☐ Dual use research of concern
☒	☐ Plants

Methods

n/a	Involved in the study
☒	☐ ChIP-seq
☒	☐ Flow cytometry
☐	☒ MRI-based neuroimaging

Clinical data

Policy information about clinical studies

All manuscripts should comply with the ICMJE guidelines for publication of clinical research and a completed CONSORT checklist must be included with all submissions.

Clinical trial registration	ClinicalTrials.gov registration NCT03100786
Study protocol	https://pmc.ncbi.nlm.nih.gov/articles/PMC6182672/
Data collection	From February 12, 2018, to March 9, 2023, 720 adults were enrolled. We recruited participants from the Hospital for Tropical Diseases, and Pham Ngoc Thach Hospital for Tuberculosis and Lung Disease, in Ho Chi Minh City, Vietnam.
Outcomes	The primary endpoint was all-cause mortality or new neurological event during 12 months from randomization. A new neurological event was defined as a Glasgow coma score reduction by ≥2 points for ≥2 days from the highest previous score, or the new onset of cerebellar symptoms, focal neurological signs, or seizures.<br>Secondary endpoints, assessed over 12 months from randomization, were overall mortality, first new neurological event, neurological disability (modified Rankin score 3-5 [Table S64), modified Rankin score as an ordinal scale, use of open-label corticosteroid treatment for any reason, serious or severe adverse events, and resolution of CSF and blood inflammation. The Rankin scale assesses dependence. A score of 0 indicated no symptoms; 1 indicated minor symptoms not interfering with lifestyle; 2 indicated symptoms that might restrict lifestyle, but patients could look after themselves; 3 indicated symptoms that restricted lifestyle and prevented independent living; 4 indicated symptoms that prevented independent living, although constant care and attention were not required; and 5 indicated total dependence on others, requiring help day and night.

Plants

Seed stocks	N/A
Novel plant genotypes	N/A
Authentication	N/A

Magnetic resonance imaging

Experimental design

Design type	Brain MRI was performed for a sub-group of participants of the LAST ACT trial. Details and results are not reported in this manuscript.
Design specifications	N/A. See above
Behavioral performance measures	N/A. See above

Acquisition

Imaging type(s)	N/A. See above
Field strength	N/A. See above
Sequence & imaging parameters	N/A. See above
Area of acquisition	N/A. See above

Diffusion MRI ☐ Used ☐ Not used

Preprocessing

Preprocessing software	N/A. See above
Normalization	N/A. See above
Normalization template	N/A. See above
Noise and artifact removal	N/A. See above
Volume censoring	N/A. See above

Statistical modeling & inference

Model type and settings	N/A. See above
Effect(s) tested	N/A. See above

Specify type of analysis: ☐ Whole brain ☐ ROI-based ☐ Both

Statistic type for inference (See Eklund et al. 2016)	N/A. See above
Correction	N/A. See above

Models & analysis

n/a	Involved in the study
☐	☐ Functional and/or effective connectivity
☐	☐ Graph analysis
☐	☐ Multivariate modeling or predictive analysis

Functional and/or effective connectivity	N/A. See above
Graph analysis	N/A. See above
Multivariate modeling and predictive analysis	N/A. See above
